# Bile Microbiota Profiling in Obese and Non-Obese Patients: A Comparison of Shotgun Metagenomics and 16S rRNA Amplicon Sequencing

**DOI:** 10.3390/life16091474

**Published:** 2026-09-03

**Authors:** Claudia Carissimi, Francesco De Angelis, Ilaria Laudadio, Valerio Fulci, Laura Stronati, Sara Manella, Domenico Alvaro, Giorgio D’Andrea, Gianfranco Silecchia, Vincenzo Cardinale

**Affiliations:** 1 Department of Molecular Medicine, Sapienza University of Rome, 00161 Rome, Italy; ilaria.laudadio@uniroma1.it (I.L.); valerio.fulci@uniroma1.it (V.F.); laura.stronati@uniroma1.it (L.S.); giorgio.dandrea@uniroma1.it (G.D.); 2 Department of Medico-Surgical Sciences and Biotechnologies, General Surgery Unit, ICOT Hospital Bariatric, 004100 Latina, Italy; fra.deangelis@uniroma1.it (F.D.A.); gianfranco.silecchia@uniroma1.it (G.S.); 3 Department of Medical and Surgical Sciences and Translational Medicine, Division of General Surgery, St. Andrea University Hospital, Sapienza University of Rome, 00185 Rome, Italy; sara.manella@uniroma1.it; 4 Department of Translational and Precision Medicine, Sapienza University of Rome, 00185 Rome, Italy; domenico.alvaro@uniroma1.it (D.A.); vincenzo.cardinale@uniroma1.it (V.C.); 5 Department of Internal Medicine, Division of Gastroenterology, Saint Louis University School of Medicine-SSM SLUCare, SLU Hospital, 1008 S. Spring, St. Louis, MO 63110, USA; 6 Department of Medicine, Harvard Medical School, 99 Brookline Ave, Boston, MA 02215, USA

**Keywords:** microbiome, bile, gallbladder, obesity, metagenomics, shotgun metagenomic sequencing and 16S rRNA gene amplicon sequencing

## Abstract

Recent advances in metagenomics have expanded our ability to detect low-abundance microbial communities. While the gut remains the most densely populated microbial habitat, emerging evidence has proposed that microorganisms might also inhabit anatomical sites once considered sterile, such as the biliary system. We apply next-generation DNA sequencing to characterize the bacterial community of bile in obese and non-obese patients with symptomatic gallstones. Bile samples were collected from 64 patients (32 obese, 32 non-obese) undergoing elective cholecystectomy. We incorporated negative (sterile tubes) and positive (mock microbial community standard) controls to evaluate contamination risks. We applied both 16S rRNA gene amplicon and shotgun metagenomic sequencing. Both sequencing methods detected extremely low bacterial biomass in bile. Specifically, shotgun metagenomic sequencing identified bacterial DNA traces in only eight samples, displaying minimal community similarity. In the positive controls, our measurements confirmed the expected microbial community composition, and in the negative controls, no bacterial DNA was detected. In contrast, 16S rRNA gene sequencing showed bacterial DNA in all bile samples as well as in negative controls, suggesting a higher susceptibility to contamination. Our findings suggest that bile may not be consistently colonized by bacterial communities in uncomplicated gallstone disease.

## 1. Introduction

Microorganisms, including bacteria, fungi, and viruses, that reside on and within the human body are collectively known as the human microbiome.

The comprehension of the relationship between the microbiome and the host in health and disease has been greatly improved in recent years. Indeed, the advent of metagenomics circumvented the uncultivability of microorganisms and filled the gap in adequate methodologies to assess microbial ecosystems with low bacterial load, allowing the identification of a broader range of microbes compared to traditional microbiological tests [1,2].

Dysbiosis, or disruption in the microbiome, has been implicated in several disease processes, including autoimmune and immune-mediated diseases [3,4], neurodegenerative disorders [5,6] and cancer [7,8].

Although the largest concentration of the human microbiome is found in the gut, the human microbiome is found in various other anatomical body sites such as the skin, the respiratory tract, and the urogenital tract [9]. Recently, the presence of microorganisms has been detected in organs or tissues traditionally considered as ‘sterile’ under healthy conditions [10]. The characterization of other human microbial niches, beyond the gut microbial environment, and the conception of ‘sterile sites’ within the human body has varied; however, to date, evidence for a structured living microbiome in most of these sites is limited or absent [11].

For example, some evidence suggests that a variety of internal biological fluids possess a native microbiota, among which human milk and blood have received particular attention [12,13,14].

The biliary tract has traditionally been considered a sterile environment under physiological conditions maintained by multiple host defense mechanisms, including bile flow, bile salt toxicity, and mucosal immunity [15,16]. However, this long-held dogma has been progressively challenged by advances in next-generation sequencing. Several studies have recently analyzed the microbiota of the biliary tract and the gallbladder and its association with bile-related diseases [17,18,19,20,21,22]. Despite this growing body of evidence, whether the human biliary environment harbors stable microbial communities remains a subject of ongoing debate, largely due to persistent methodological concerns: sampling route and post-sphincterotomy contamination, antibiotic prophylaxis, low microbial biomass, and heterogeneous analytical pipelines represent significant confounders that have hampered firm conclusions [23,24]. Obesity has recently emerged as one of the most important global health challenges [25] and represents one of the strongest and most well-established risk factors for cholesterol gallstone disease, primarily driven by hepatic oversecretion of cholesterol leading to bile supersaturation [26,27,28,29]. Obesity is one of the strongest modifiable risk factors for cholesterol gallstone disease, with a dose-dependent relationship (OR up to 3.38 for BMI > 30), driven by hepatic cholesterol hypersecretion, bile supersaturation, impaired gallbladder motility, and insulin resistance [28].

Beyond its metabolic effects, obesity is associated with profound alterations in gut microbiota composition and bile acid metabolism [30,31,32], and there is emerging evidence that such alterations may contribute to obesity-related metabolic dysfunction [33]. Given that obesity profoundly alters both the systemic metabolic milieu and the gut–biliary axis, it was biologically plausible that obesity-specific microbial signatures might exist within the biliary compartment itself.

In this context, the potential impact of obesity on the biliary microbiota represents a largely unexplored yet clinically relevant question. The aim of our study was to characterize the bacterial community in obese and non-obese patients with uncomplicated cholelithiasis using both shotgun metagenomic sequencing and 16S rRNA gene amplicon sequencing applied to 64 bile samples. To minimize the risk of contamination—a major limitation of previous studies—bile was collected aseptically from intact gallbladders during surgery, thereby avoiding ERCP-related contamination, and rigorous negative and positive controls were included throughout.

## 2. Materials and Methods

### 2.1. Patient Recruitment and Inclusion/Exclusion Criteria

Human bile samples were obtained from the gallbladders of patients who underwent a cholecystectomy between January 2021 and June 2022 at the Division of General Surgery of S. Andrea University Hospital, Rome, and ICOT Hospital Latina, Italy. Patients were divided into two groups: a group of 32 obese patients (BMI between 30 and 50) and a group of 32 non-obese patients, all affected by symptomatic uncomplicated gallstones and thus candidates for elective cholecystectomy [34,35]. The patients did not present any criteria suggestive of acute lithiasis cholecystitis [34,35]. None of the enrolled patients underwent ERCP or papillotomy prior to cholecystectomy.

Following current guidelines, all patients received a standard prophylactic dose of 2 g of cefazolin intravenously before anesthesia. No additional antibiotic doses were administered perioperatively, and no patients received antibiotic treatment in the 3 months prior to surgery, which was an exclusion criterion for our study (Supplementary Table S1).

The criteria for inclusion and exclusion from the study are given in Supplementary Table S1.

All anthropometric and clinical data of the patients, summarized in Supplementary Table S2, were collected, cataloged, and maintained in an electronic database in full privacy.

All patients enrolled in the study signed a specific informed consent. The study was approved by the Ethical Committee of the Sapienza University of Rome (CET Lazio 1-Prot. 0364/2026-29/04/2026).

### 2.2. Sample Collection and Storage

Bile samples were collected following a strict protocol to ensure aseptic conditions and to avoid possible microbial contamination from the environment.

Sixty-four eligible patients underwent laparoscopic cholecystectomy surgery according to standard technique [36].

Bile samples were collected at the time of surgery from the intact gallbladder in an endobag device and then on a sterile field. Bile (5–10 mL) was aspirated from the gallbladder with a sterile 20 mL syringe. Samples were stored in sterile tubes in −80° freezers until DNA isolation. No patients had complications or postoperative mortality.

### 2.3. DNA Isolation

The bacterial DNA was isolated from 250 uL of bile samples utilizing the ZymoBIOMICS DNA Miniprep Kit (Zymo Research, Irvine, CA, USA) according to the manufacturer’s instructions.

To address laboratory and sequence-based artifacts that can occur with reagents and kits, DNA was also extracted from a commercially available mock microbial community standard (ZymoBIOMICS™) (positive controls) and from no-sample-added sterile tubes (negative controls).

After the isolation, DNA was quantified. The DNA isolation workflow was carried out under a laminar flow hood in a clean and sterile environment. The research team changed gloves and pipette tips between samples to minimize cross-contamination.

### 2.4. Shotgun Metagenomic Sequencing and 16S rRNA Gene Amplicon Sequencing

Library preparation and sequencing were performed at Istituto di Genomica Applicata (IGA-Udine, Udine, Italy). For shotgun metagenomics analysis, microbial DNA was sequenced by Paired End (150 bp) sequencing at a depth of at least 10 M reads per sample using Illumina technology.

For 16S analysis, the primer pair sequences for the 16S V3 and V4 regions were used; PNA oligos were used as a sequence-specific PCR blocker. The same amount of DNA, as assessed by Qubit measurement, was processed for each sample. 16S Metagenomic Sequencing Library Preparation protocol by Illumina was followed. Amplicons were sequenced on NovaSeq 6000 to generate 250-bp reads.

The data can be accessed at the following link: https://ega-archive.org/studies/EGAS50000002005 (accessed on 5 August 2026).

### 2.5. Bioinformatic Analysis

Raw fastq files were quality checked using FastQC v 0.12.1 (https://www.bioinformatics.babraham.ac.uk/projects/fastqc/ (accessed on 5 August 2026)).

For the 16S rRNA amplicons, Kraken2 v. 2.1.3 [37] was run on the fastq data, using the SILVA rRNA database [38] as reference.

For the shotgun metagenomics data, MetaPhlAn v. 4.0.6 [39] was used.

Human DNA contaminant sequences were assessed using Kneaddata v 0.12.0 (https://github.com/biobakery/kneaddata (accessed on 5 August 2026)).

Kraken2 output was further analyzed using phyloseq [40] and vegan (https://cran.r-project.org/web/packages/vegan/vegan.pdf (accessed on 5 August 2026)) R Packages. These tools were used to compute alpha diversity according to Chao1 [41] and Shannon indices, to plot the heatmap (according to Non-metric MultiDimensional Scaling (NMDS) ordination [42], with Bray–Curtis distance [43]) and to plot sample ordination according to Principal Coordinate Analysis (PCoA) [44] and NMDS ordination using Bray–Curtis distance. Linear discriminant analysis Effect Size (LEfSe) [39] was computed using the microbiomeMarker R package [45].

As reported in the results section, less abundant taxa were filtered according to relative abundance. To account for the different sequencing depth of the samples, rather than setting a cutoff on the raw counts of each sample, we filtered the dataset with commonly used criteria. In particular, only taxa occurring in at least 20% of the samples at a frequency higher than 0.1% were retained.

## 3. Results

### 3.1. Clinical Characteristics of Study Subjects

Sixty-four patients, divided into two groups, 32 obese (BMI > 30) and 32 non-obese (BMI < 30), all affected by symptomatic uncomplicated gallstones (no cholecystitis or cholangitis), underwent elective laparoscopic cholecystectomy. All the procedures were carried out by the senior surgeon.

Both patient cohorts were homogeneous in age, gender, and comorbidities (Supplementary Table S2). No mortality or post-operative complications were reported after surgery.

### 3.2. Microbiome Composition Analysis by 16S rRNA Gene Amplicon Sequencing

To explore and compare the microbial profile of bile samples taken from obese and non-obese patients affected by uncomplicated gallstones, we performed 16S rRNA amplicon sequencing of total DNA isolated from 20 bile samples (10 obese and 10 non-obese patients). Two positive and three negative controls were also analyzed. Positive controls consisted of a commercially available mock microbial community standard (ZymoBIOMICS™), including eight bacterial strains and two yeasts. Negative controls consisted of sterile tubes with no sample added. Bile samples, negative and positive controls, were processed in parallel.

**Figure 1:**
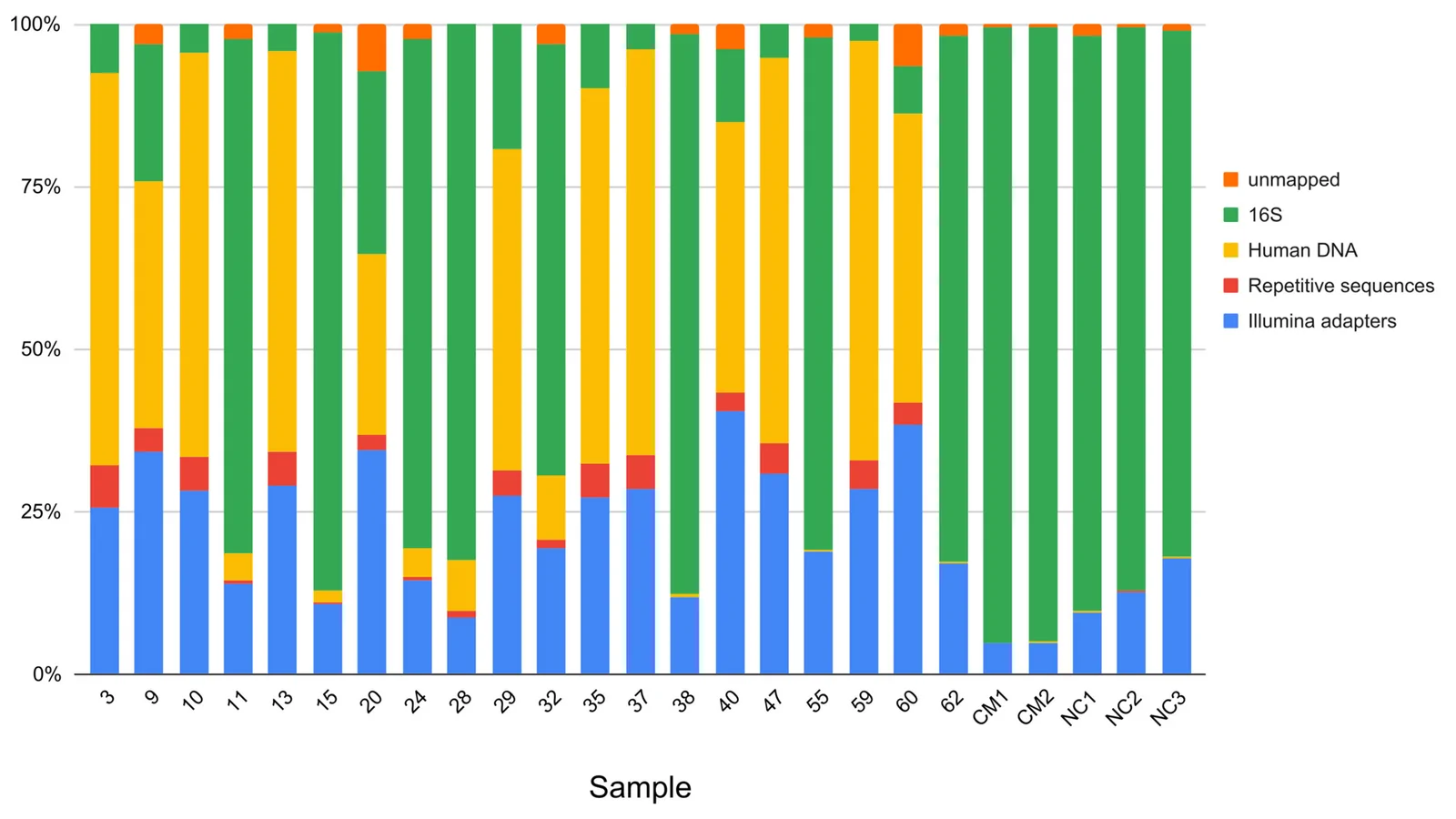
Classification of DNA reads obtained from 16S amplicon sequencing for each sample. The histogram displays the percentage of reads that: match Illumina adapters (blue); repetitive sequences (red); map to the human genome (yellow); map to the SILVA 16S database (green). Reads not matching any of these categories are reported in orange. NC: negative control; CM: microbial community standard.

Our analysis showed a large fluctuation in the relative abundance of bacteria in bile samples, with bacterial read percentages ranging from 2.5% to 86.1% (Table 1 and Figure 1). Additionally, we detected human reads that varied between 0.04% and 64.7%. In contrast, bacterial reads in positive controls accounted for a median of 94.6%, while human DNA accounted for less than 0.05% of the total sequenced reads (Table 1 and Figure 1).

To determine whether the variability in the proportions of human and bacterial DNA in the final amplification product was linked to the concentration of DNA used as input for the PCR reactions, a correlation analysis was performed between the input DNA concentration and the fraction of human DNA in the amplified samples. As shown in Supplementary Figure S2, no direct correlation was observed between these two parameters. This finding indicates that the presence of high levels of human DNA and reduced amounts of bacterial DNA in some samples is not due to variations in input DNA concentration.

Unexpectedly, bacterial DNA was also detected in negative controls. In these samples, reads aligning against the SILVA 16S database reference accounted for a median of 84.6% of the reads per sample, whereas human DNA comprised 0.1% of the total sequenced reads (Table 1 and Figure 1).

Taxonomic annotation of the three negative controls identified a total of 92, 91, and 73 different genera with an abundance > 0.1%, respectively; among them, the most abundant are Candidatus Symbiobacter and Burkholderia-Caballeronia-Paraburkholderia (Supplementary Table S3). Furthermore, the comparison between the measured composition and the theoretical composition of the microbial community standard samples (positive controls) revealed the presence of foreign taxa in our taxonomic analysis (Supplementary Table S4). Considering a cut-off of abundance > 0.1%, we detected a further 25 and 23 genera, most of which (22 genera) are present in both the positive controls (Supplementary Figure S1).

**Figure 2:**
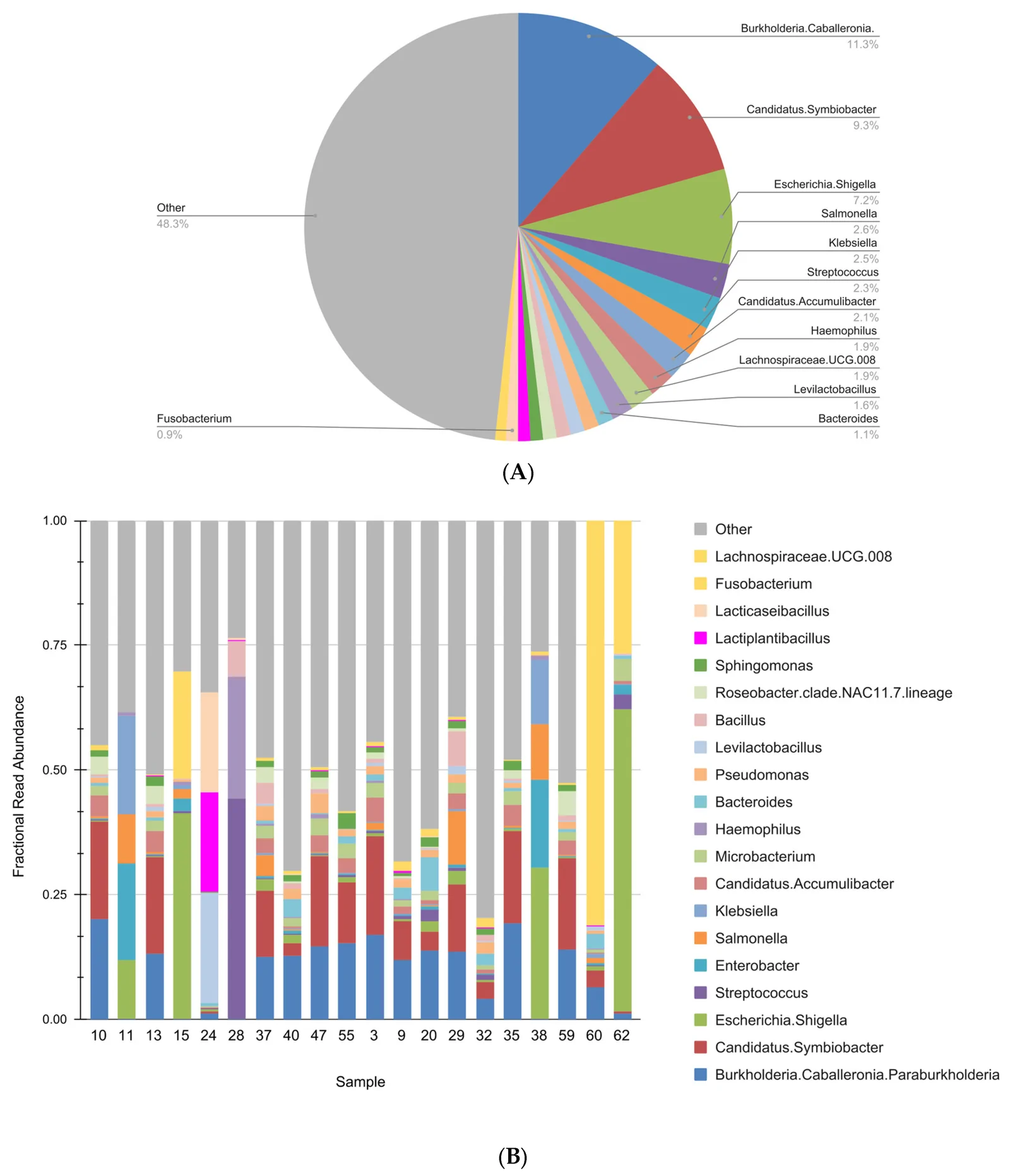
Metagenome composition of bile samples analyzed by 16S rRNA amplicon sequencing. (**A**) Pie chart showing the cumulative fractional abundance of the top 20 most abundant taxa in the overall dataset. (**B**) The top 20 most abundant taxa in the overall dataset are reported according to the different study groups (sample 10–55 non-obese, sample 3–62 obese). Taxonomy is reported at the genus level.

In bile samples, a total of 262 genera were identified using a cut-off of abundance > 0.1% in at least one sample. The mean number of genera per sample was 33.4, STDdev: 31.26 (range: 6–134), with notable inter-sample variability in abundance (Supplementary Table S5). As illustrated in Figure 2A,B, the microbial community was dominated by Burkholderia-Caballeronia-Paraburkholderia (11.3%), Candidatus Symbiobacter (8.3%), and Escherichia-Shigella (7.2%), which collectively accounted for the most abundant taxa across the dataset. Notably, the two most prevalent genera in bile samples were identical to those detected in negative controls.

An LEfSe analysis was performed to identify any significantly abundant bacterial taxa between the obese and non-obese groups. With an LDA score threshold of 2 (*p*-value < 0.05), the analysis did not identify any differentially abundant features between the analyzed groups.

**Figure 3:**
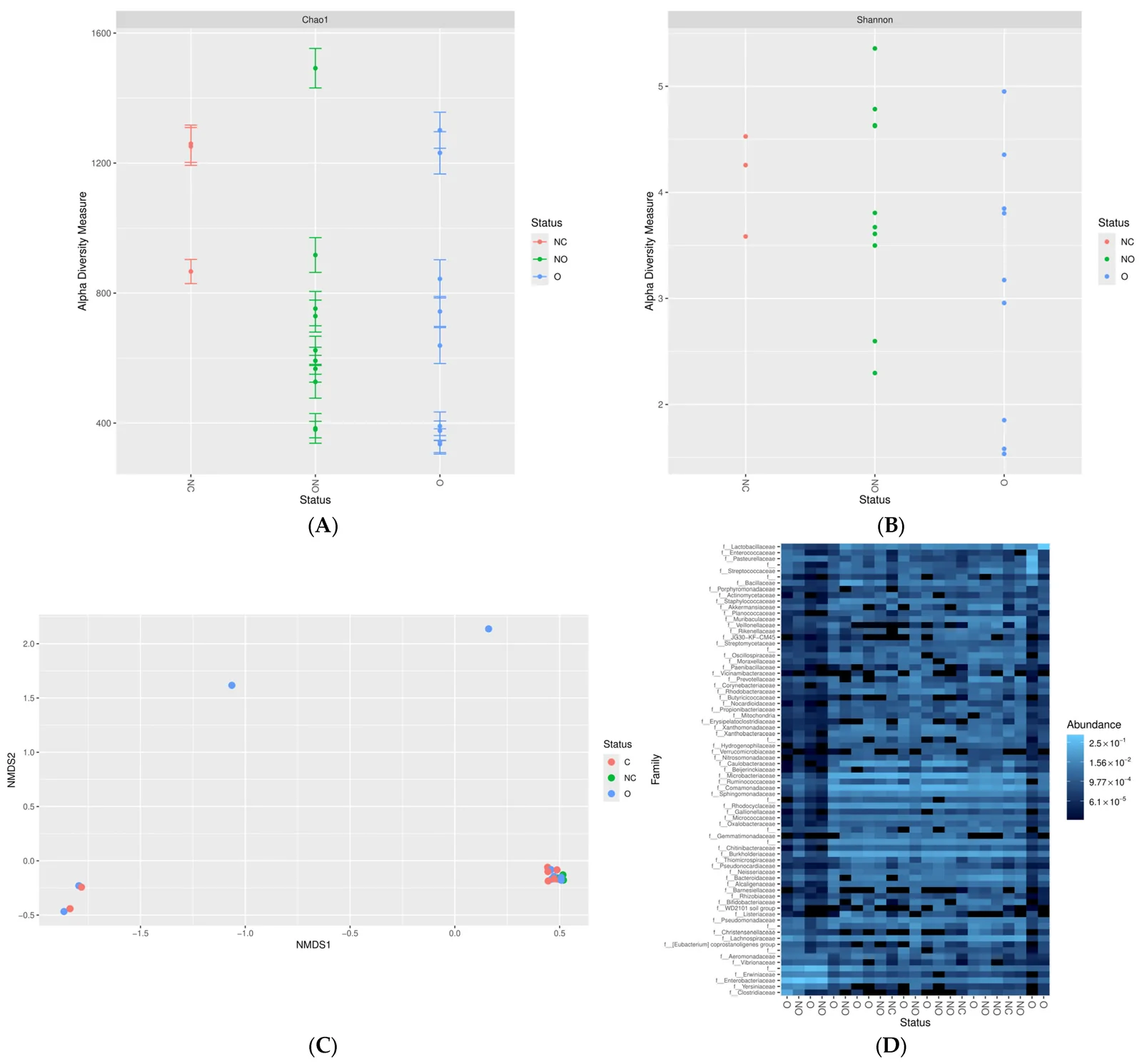
Comparison of microbial diversity and composition between study groups. (**A**,**B**) Alpha diversity analysis using Chao1 and Shannon indexes showed no significant differences in bacterial richness and evenness between negative controls, non-obese and obese patients. (**C**) Beta diversity analysis using NMDS showed no significant difference between negative controls, non-obese and obese patients. (**D**) Heatmap representing the abundance of bacterial families (>0.1% of total identified bacteria in at least 20% of samples) across all samples. No distinct clustering patterns were observed among the three groups, indicating that the overall microbial community composition cannot differentiate between negative controls, non-obese, and obese patients. Blue, red, and green colors represent obese, non-obese, and negative control samples, respectively.

We also analyzed the microbial community composition in negative controls, obese and non-obese patients. We compared the bacterial richness and evenness between these groups by Chao 1 and Shannon index analysis. As shown in Figure 3A,B, no significant differences were detected in alpha diversity measures between non-obese and obese patients nor between non-obese or obese patients and negative controls. Similarly, the beta-diversity by *NMDS* analysis failed to distinguish between negative controls, obese, and non-obese patient groups (Figure 3C).

Moreover, we compared the taxonomic composition of negative controls, obese and non-obese patients. We analyzed the abundance of families representing >0.1% of the total identified bacteria in at least 20% of the samples. No clustering of samples into groups with similar microbial patterns was observed, suggesting that overall bacterial composition does not allow distinguishing between negative controls, non-obese, and obese patients (Figure 3D).

Overall, our analysis failed to show any significant differences between bile samples and negative controls, thereby preventing the identification of a core microbiota in the human bile.

### 3.3. Microbiome Profiling by Shotgun Sequencing Does Not Support the Existence of a Core Bile Microbiota

To investigate more deeply the bile microbiota, we increased the sample size, and we took advantage of the shotgun metagenomic sequencing approach. This method is widely recognized as providing a more accurate characterization of microbiome complexity compared to 16S rRNA amplicon sequencing [46,47].

Overall, shotgun metagenomic sequencing was performed on 64 DNA samples isolated from the bile of 32 obese patients and 32 non-obese patients, three negative controls, and two positive controls. Twenty out of the 64 bile DNAs, as well as DNA from negative and positive controls, were the same as those previously analyzed by 16S rRNA sequencing.

**Figure 4:**
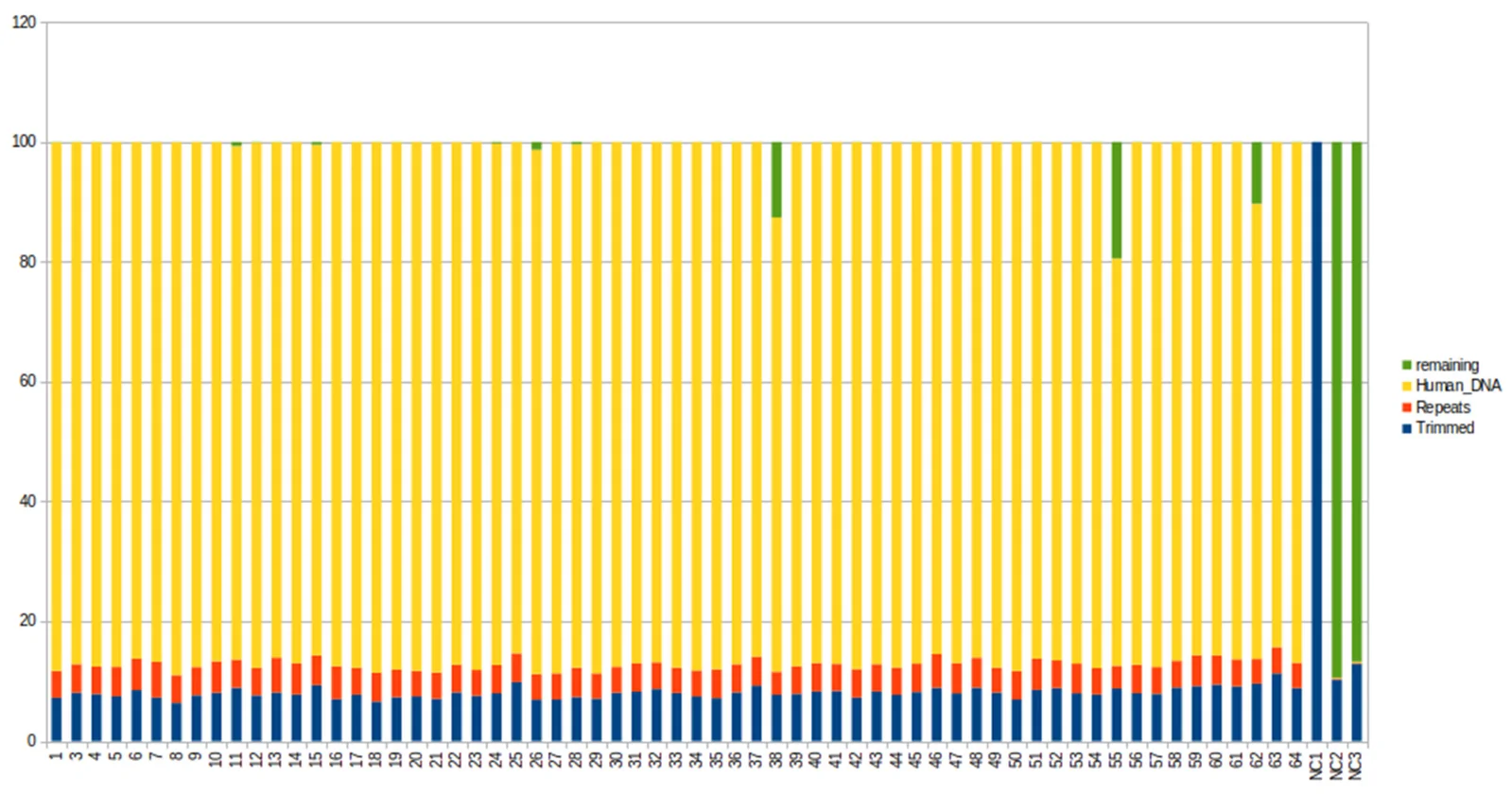
Percentage mapping of reads obtained from shotgun metagenomic analysis. The histogram displays the percentage of reads that: match Illumina adapters (blue); consist of low complexity repeats (orange); map to the human genome (yellow); map to the metagenome (green).

We obtained an average of 12.6 million PE reads per sample (range: 8.9–34.1). After adapter trimming, reads were filtered to remove host components and then classified using Metaphlan 4.0.6. As shown in Figure 4, the contamination by human DNA represents 85.12% of total sequenced reads, and only 0.7% of reads could be used for alignment to the bacterial database.

We found reads aligning to the bacterial database in only 8 out of 64 samples (specifically, samples 11, 15, 24, 26, 28, 38, 55, and 62). Taxonomic profiles for every single sample are reported in Table 2.

We found minimal similarity among bacterial communities among these samples, with a large proportion of bacteria unique to each sample. In particular, we observed that in 5 out of 8 samples more than 96% of reads arose from a single species, which is different for each sample, namely Enterobacter hormaechei, Escherichia coli, Lacticaseibacillus|S, Bifidobacterium animalis, and Enterococcus faecium. In the other three samples, we identified 3, 6, and 12 species, respectively, with a limited overlap. In the other 56 patient samples, no presence of bacterial DNA was detected.

In the three negative controls, no bacterial DNA was detected, except for a single read pair mapped to a bacterial genome identified as Cutibacterium. Since Cutibacterium is a common skin commensal, it was considered a probable contaminant originating from laboratory reagents or handling.

**Figure 5:**
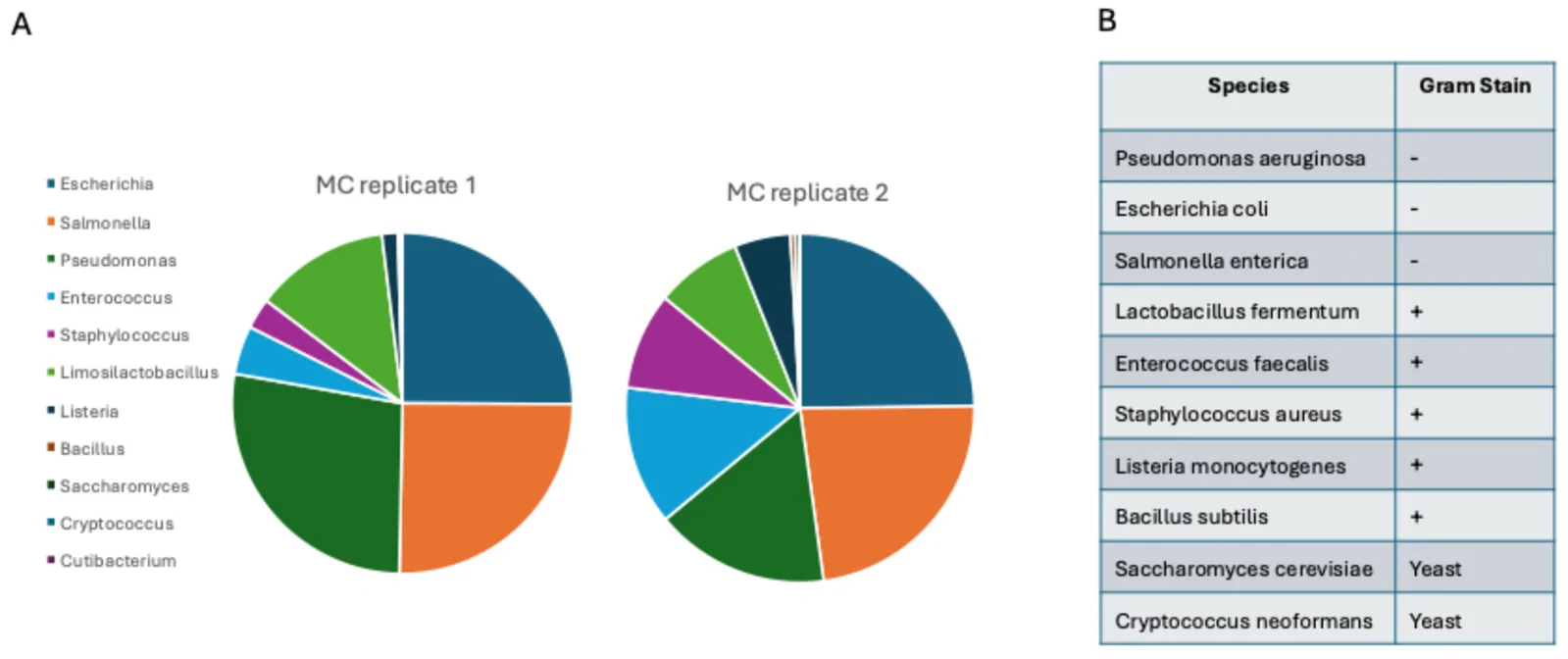
Taxonomic annotation of positive control samples analyzed by Shotgun metagenomic sequencing. Community composition of positive controls (**A**) and expected composition of ZymoBIOMICS^TM^ Microbial Community Standard (Zymo) (**B**).

Notably, in the positive controls, our measurements confirmed the expected microbial community composition, consisting of eight bacterial strains and two yeasts, with no detection of foreign species. The only exception was one of the positive controls, which contained DNA from Cutibacterium, which was also amplified in one negative control sample. This result supports the accuracy of our DNA isolation workflow (Figure 5).

Overall, our findings do not support the existence of resident bacterial communities in bile, either in non-obese or in obese patients.

## 4. Discussion

To characterize the bacterial community of gallbladder bile in obese and non-obese patients, we analyzed bile samples collected from a total of 64 patients, including 32 obese and 32 non-obese patients, all diagnosed with uncomplicated gallstones. The study also included three negative controls (no-sample added sterile tubes) and two positive controls (mock microbial community standard).

Samples were analyzed using deep metagenomic sequencing of total DNA. Additionally, a subset of 20 samples, along with positive and negative controls, was analyzed using both shotgun metagenomic sequencing and 16S rRNA gene amplicon sequencing.

In our study, both sequencing techniques detected extremely low bacterial biomass in DNA extracted from bile samples. In shotgun metagenomic sequencing, nearly all the sequenced reads were identified as human DNA, while in 16S sequencing, human reads varied between 0.04% and 64.7%, suggesting either a negligible microbial presence or a complete absence of microorganisms in bile. Notably, in both approaches, the positive controls confirmed the expected microbial community composition, validating our lysis methods.

In 16S rRNA amplicon sequencing, although microbial communities were detected in all samples, the considerable variation in bacterial relative abundance across the analyzed samples, the presence of bacterial species in negative extraction controls, and the detection of foreign taxa in positive control samples underscore a significant contamination risk.

Indeed, we found that the two most abundant taxa were identical in both negative controls and bile samples from multiple subjects enrolled: Burkholderia-Caballeronia-Paraburkholderia and Candidatus Symbiobacter. Furthermore, no distinct separation was observed in the overall composition of the bile microbiota between the negative controls, obese patients, and non-obese patients.

To improve detection and characterization of the bile microbial communities in our samples, we expanded the sample set and applied shotgun metagenomic sequencing. This technique is widely recognized as providing a more precise characterization of microbiome complexity compared to 16S rRNA amplicon sequencing. However, shotgun metagenomic sequencing also failed to detect a bile microbiome in most of our samples. Bacterial DNA traces were detected in only 8 out of 64 bile samples. Remarkably, the bacterial taxa identified in each of these samples differed in identity. The sole signal consistently detected was the expected microbial community in the positive control, validating the experimental workflow and minimizing the likelihood of technical artifacts influencing the analysis. These results provide no consistent evidence of bacterial communities residing in the bile.

It is important to note that 20 out of the 64 bile DNA samples, as well as DNA from both negative and positive controls, were the same samples previously analyzed using 16S rRNA sequencing. Overall, the comparison of the results obtained by 16S rRNA gene amplicon and shotgun metagenomics sequencing suggests that signals detected by 16S rRNA gene amplicon sequencing may represent false-positive results. In microbiota studies utilizing 16S rRNA gene amplicon sequencing of low-biomass samples, several publications have highlighted the risk for erroneously interpreting background DNA as originating from bacteria within the sample [48,49,50]. This can be attributed to the experimental biases of 16S rRNA gene amplicon sequencing of low- or no- biomass samples; background DNA can outcompete low-copy-number- or no-DNA from the sample itself, resulting in over-amplification during the PCR process, and leading to spurious sequencing results [35,36]. In contrast, shotgun metagenomics studies do not suffer from this bias since they do not involve a targeted PCR amplification step.

Overall, these results suggest that the detected microbial signals obtained by 16S rRNA gene amplicon sequencing are likely the result of contamination during sample collection or during DNA extraction or sequencing.

It is important to note that our study was designed to investigate bile samples from obese versus non-obese subjects. To our knowledge, this is the first study to report stringent criteria aimed at avoiding subjects with ongoing biliary infection due to cholecystitis. These considerations are relevant to interpreting these results within the context of the existing literature. Indeed, our findings appear to contrast with previous studies that, based on 16S rRNA gene amplicon sequencing, have suggested that the bile is colonized by a diverse population of bacteria commonly referred to as the ‘bile microbiome’. It is important to point out that none of the previous studies report the sequencing of 64 human bile samples by shotgun metagenomics, nor have they compared the results of shotgun metagenomic sequencing with 16S rRNA gene amplicon sequencing on the same set of bile samples, including both negative and positive controls. It is worth mentioning that our samples were collected under sterile conditions to minimize contamination, with bile aspirated aseptically during surgery from intact gallbladders placed in a sterile field. In contrast, the majority of the previous studies collected the bile samples via endoscopic retrograde cholangiopancreatography (ERCP), a procedure recently shown to carry a high risk of translocating microbes from the oral cavity and intestines into the biliary system [51].

Additionally, to assess how much background bacterial DNA was present in the samples and the accuracy of our workflow, we sequenced DNA isolated from sterile tubes without added samples (negative controls) and DNA from a commercially available mock microbial community standard (positive controls). In the literature, few studies on the microbiome have included controls to assess the impact of DNA contamination [51,52,53,54]. Moreover, we employed two different approaches that do not suffer from the same limitations.

It is noteworthy that the biliary barrier shares several immune defense mechanisms with the intestine, including secretory immunoglobulin A (IgA), defensins, and Toll-like receptor (TLR)-mediated immune activation [17,55]. Moreover, the intrinsic toxicity of bile further limits bacterial colonization; however, Gram-negative bacteria such as Salmonella spp., Escherichia coli, and Helicobacter species exhibit greater bile resistance compared to Gram-positive species [17].

We are aware that our study has limitations. It did not include two independent DNA isolations for each sample, the use of different extraction methods, or sequencing of negative controls from various stages of the process, all of which could have enabled the recognition and curation of the full spectrum of potential contaminants. Microscopy or bacterial culture were not incorporated to visualize and verify the viability of bacteria in bile. These factors may have limited the results.

In conclusion, in a study of 64 bile samples carefully collected and analyzed by both shotgun metagenomic sequencing and 16S rRNA gene amplicon sequencing, our findings suggest that bile may not be consistently colonized by bacterial communities in uncomplicated gallstone disease, challenging the concept of a ‘bile microbiome’ as proposed in previous studies. This conclusion is supported by recent studies that question the existence of a core microbiome in low-biomass samples such as placenta and bile, emphasizing the importance of careful methodological considerations in microbiome research [11,56,57,58,59]. Further research, involving functional and quantitative evaluations of the bacterial communities present in the bile, is warranted; indeed, genomic techniques confirm only the presence of microorganisms and not their vitality. In medical and microbiological fields, it is crucial to differentiate between the terms “aseptic” and “DNA-free”, as they represent fundamentally distinct concepts with significant implications for research and clinical practice. While sterilization processes eliminate viable microorganisms, they do not necessarily remove all microbial genetic material. This distinction is particularly critical in microbiological contexts, where residual DNA can significantly influence experimental outcomes and interpretations.

## Figures and Tables

**Table 1:** Table reporting for each 16S library: the number of reads sequenced for each library, the number of reads containing only adapters (“trimmed”), the number of reads consisting of repetitive sequences, the number of reads mapping to the human genome, the number of reads mapped to the SILVA 16S database, and the number of unmapped reads.

Sample	Reads Sequenced	Illumina Adapters	Repetitive Sequences	Human DNA	16S	Unmapped	Median Phred Quality	Average Phred Quality
3	426,296	108,711	28,151	257,200	32,046	188	37	35.04
9	148,690	50,841	5490	56,369	31,178	4812	37	33.16
10	336,418	95,104	17,288	209,232	14,726	68	37	34.5
11	537,072	75,199	2153	22,955	423,982	12,783	37	32.17
13	285,158	82,947	14,363	175,700	12,120	28	37	34.45
15	646,362	69,430	1466	12,320	554,785	8361	37	31.85
20	47,112	16,223	1165	13,029	13,272	3423	37	27.8
24	290,154	41,840	1530	12,956	226,928	6900	37	33.73
28	632,392	55,635	5216	49,855	521,207	479	37	35.93
29	408,932	111,939	16,366	201,600	78,657	370	37	34.67
32	295,592	57,530	3444	29,228	196,332	9058	37	34.26
35	370,062	100,167	19,139	214,322	36,202	232	37	34.73
37	521,096	148,716	26,505	325,126	20,576	173	37	34.49
38	315,000	37,068	481	1252	271,216	4983	37	30.86
40	172,160	69,521	5146	71,491	19,426	6576	37	28.45
47	272,216	83,727	13,129	161,494	13,685	181	37	34.49
55	395,794	74,358	560	179	312,363	8334	37	33.88
59	359,484	102,080	15,851	232,564	8927	62	37	34.36
60	262,110	100,569	8490	116,947	19,011	17,093	37	32.51
62	215,214	36,394	464	329	173,889	4138	37	29.99
CM1	1,102,162	53,081	630	331	1,043,559	4561	37	36.14
CM2	897,526	43,423	518	616	849,167	3802	37	36.16
NC1	504,278	47,191	572	641	447,180	8694	37	36.17
NC2	391,478	49,141	1110	316	338,467	2444	37	36.2
NC3	638,856	113,438	754	639	516,770	7255	37	36.23

**Table 2:** Taxonomic profiles of samples analyzed by Shotgun metagenomic sequencing. The numerical values indicate the percentage of reads mapping to each indicated genome.

Clade Name	Sample 11	Sample 15	Sample 24	Sample 26	Sample 28	Sample 38	Sample 55	Sample 62
Enterobacter_hormaechei	100.00	0.00	0.00	0.00	0.00	0.00	0.00	0.00
Escherichia_coli	0.00	67.48	0.00	0.00	0.00	28.22	45.46	99.22
Fusobacterium_nucleatum	0.00	24.88	0.00	0.00	0.00	0.00	18.81	0.00
Clostridium_perfringens	0.00	7.64	0.00	0.00	0.00	0.00	0.00	0.53
Lacticaseibacillus|s__S	0.00	0.00	100.00	0.00	0.00	0.00	0.00	0.00
Bifidobacterium_animalis	0.00	0.00	0.00	100.00	0.00	0.00	0.00	0.00
Enterococcus_faecium	0.00	0.00	0.00	0.00	96.21	0.00	0.00	0.00
Haemophilus_parainfluenzae	0.00	0.00	0.00	0.00	3.79	0.00	0.00	0.00
Klebsiella_michiganensis	0.00	0.00	0.00	0.00	0.00	63.69	0.00	0.00
Klebsiella_pneumoniae	0.00	0.00	0.00	0.00	0.00	3.32	0.00	0.00
Citrobacter_freundii	0.00	0.00	0.00	0.00	0.00	3.24	0.00	0.00
Aeromonas_caviae	0.00	0.00	0.00	0.00	0.00	1.46	0.00	0.00
Enterobacter_roggenkampii	0.00	0.00	0.00	0.00	0.00	0.08	0.00	0.00
Enterobacter_kobei	0.00	0.00	0.00	0.00	0.00	0.00	14.00	0.00
Streptococcus_anginosus	0.00	0.00	0.00	0.00	0.00	0.00	12.53	0.00
Veillonella_parvula	0.00	0.00	0.00	0.00	0.00	0.00	7.04	0.00
Enterococcus_faecalis	0.00	0.00	0.00	0.00	0.00	0.00	0.75	0.25
Eikenella_corrodens	0.00	0.00	0.00	0.00	0.00	0.00	0.50	0.00
Actinomyces_radicidentis	0.00	0.00	0.00	0.00	0.00	0.00	0.42	0.00
Listeria_monocytogenes	0.00	0.00	0.00	0.00	0.00	0.00	0.27	0.00
Prevotella_buccae	0.00	0.00	0.00	0.00	0.00	0.00	0.17	0.00
Campylobacter_SGB19298	0.00	0.00	0.00	0.00	0.00	0.00	0.03	0.00
Acidipropionibacterium_acidipropionici	0.00	0.00	0.00	0.00	0.00	0.00	0.03	0.00

## Data Availability

The data can be accessed at the following link: https://ega-archive.org/studies/EGAS50000002005 (accessed on 5 August 2026).

## Supplementary Materials

The following supporting information can be downloaded at: https://www.mdpi.com/article/10.3390/life16091474/s1, Figure S1: Representation of foreign genera detected in Microbial Community Standard samples; Figure S2: Human DNA; Table S1: Study inclusion criteria; Table S2: Demographics; Table S3: Taxonomic annotation of negative control samples analyzed by 16S rRNA amplicon sequencing; Table S4: Taxonomic annotation of positive control samples (Microbial Community Standard) analyzed by 16S rRNA amplicon sequencing; Table S5: 16S_summary.
