# Supplementary material for: Bile Microbiota Profiling in Obese and Non-Obese Patients: A Comparison of Shotgun Metagenomics and 16S rRNA Amplicon Sequencing
Source: Life (Basel). 2026 Sep 3;16(9):1474. doi: 10.3390/life16091474 (PMC13608661; doi:10.3390/life16091474)

Supplementary Figure S1 **Representation of foreign genera detected in Microbial Community Standard samples. The Venn diagram compares measured versus theoretical composition, showing foreign genera (abundance > 0.1%) present in positive controls MC1 (n=25) and MC2 (n=23) with 22 of these genera present in both controls.**

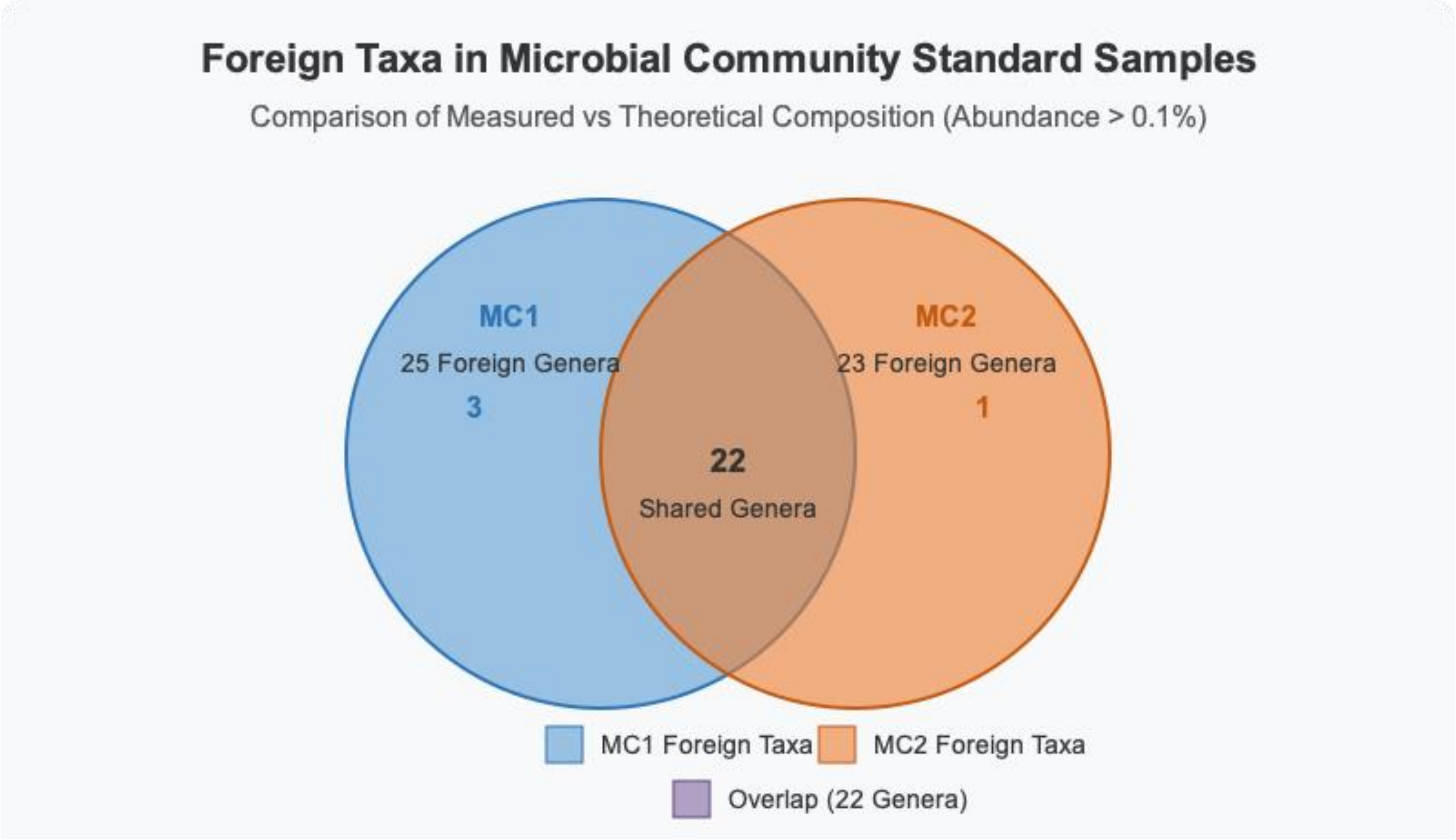

Supplement: Supplementary file 1 [file life-16-01474-s001.zip › Suppl Figure S1.pdf]
