# Supplementary material for: Bile Microbiota Profiling in Obese and Non-Obese Patients: A Comparison of Shotgun Metagenomics and 16S rRNA Amplicon Sequencing
Source: Life (Basel). 2026 Sep 3;16(9):1474. doi: 10.3390/life16091474 (PMC13608661; doi:10.3390/life16091474)
Supplement: Supplementary file 1 [file life-16-01474-s001.zip › Suppl Table S4.pdf]

Supplementary Table S4. Taxonomic annotation of positive control samples (Microbial Community Standard) analyzed by 16S rRNA amplicon sequencing

Sample	SampleCM1	SampleCM2
Salmonella	0,213561208	0,212394336
Bacillus	0,207108008	0,205158576
Listeria	0,117011408	0,114047472
Staphylococcus	0,088786104	0,083914992
Levilactobacillus	0,079404144	0,085550472
Escherichia-Shigella	0,075780424	0,074756304
Limosilactobacillus	0,03087608	0,029963976
Pseudomonas	0,026170208	0,026990376
Lachnospiraceae UCG-008	0,02045168	0,019824
Streptococcus	0,013611288	0,015799728
Lactiplantibacillus	0,01087116	0,010417512
Enterobacter	0,008607576	0,008900976
Enterococcus	0,008180672	0,007652064
Anoxybacillus	0,007833192	0,00763224
Lactobacillus	0,007783552	0,008098104
Klebsiella	0,006790752	0,0071862
Virgibacillus	0,00431868	0,00436128
Geobacillus	0,0042194	0,004252248
Lysinibacillus	0,004159832	0,004440576
Ligilactobacillus	0,004100264	0,00406392
Latilactobacillus	0,00377264	0,003201576
Saliterribacillus	0,003762712	0,004341456
Citrobacter	0,0027302	0,002616768
Pseudogracilibacillus	0,002402576	0,002101344
Serratia	0,002392648	0,002289672
Pantoea	0,002204016	0,00218064
Pediococcus	0,002164304	0,00213108
Gracilibacillus	0,001419704	0,000981288
Kosakonia	0,001280712	0,00109032
Tenuibacillus	0,00119136	0,001100232
Lacticaseibacillus	0,001171504	0,001348032
Vibrio	0,001111936	0,000961464
Jeotgalibaca	0,001012656	0,000901992
Yersinia	0,000883592	0,00104076
