# Supplementary material for: Bile Microbiota Profiling in Obese and Non-Obese Patients: A Comparison of Shotgun Metagenomics and 16S rRNA Amplicon Sequencing
Source: Life (Basel). 2026 Sep 3;16(9):1474. doi: 10.3390/life16091474 (PMC13608661; doi:10.3390/life16091474)
Supplement: Supplementary file 1 [file life-16-01474-s001.zip › SUPPLEMENTARY DATA_legends.pdf]

SUPPLEMENTARY DATA

Supplementary Figure S1. Representation of foreign genera detected in Microbial Community Standard samples. The Venn diagram compares measured versus theoretical composition, showing foreign genera (abundance > 0.1%) present in positive controls MC1 (n=25) and MC2 (n=23) with 22 of these genera present in both controls.

supplementary Table S1. The criteria for inclusion and exclusion to the study
supplementary Table S2. Anthropometric and clinical data of the patients
Supplementary Table S3. Taxonomic annotation of negative control samples analyzed by 16S rRNA amplicon sequencing
Supplementary Table S4. Taxonomic annotation of positive control samples (Microbial Community Standard) analyzed by 16S rRNA amplicon sequencing
supplementary Table S5. Bacterial genera identified in bile samples analyzed by 16S rRNA amplicon sequencing, using an abundance threshold of >0.1% in at least one sample.
