# Supplementary material for: Bile Microbiota Profiling in Obese and Non-Obese Patients: A Comparison of Shotgun Metagenomics and 16S rRNA Amplicon Sequencing
Source: Life (Basel). 2026 Sep 3;16(9):1474. doi: 10.3390/life16091474 (PMC13608661; doi:10.3390/life16091474)
Supplement: Supplementary file 1 [file life-16-01474-s001.zip › Supplementary Table S1 Study inclusion criteria .pdf]

Supplementary Table S1. Study inclusion criteria

Exclusion Criteria

	Details
Antibiotic Therapy	Within 3 months prior to surgery
Corticosteroid/NSAID Therapy	For at least 4 weeks within the last 3 months
Acute Cholecystitis	
Cholecysto-choledocholithiasis	
Intraoperative Gallbladder Rupture	
History of Cancer	Within the last 3 years
Inflammatory Bowel Disease (IBD)	

Inclusion Criteria

Age	> 18 and < 70 years old
Obesity	BMI > 30 and < 50
Type of Surgery	Elective surgery
Indication for Surgery	Any indication for cholecystectomy
