# Supplementary material for: Bile Microbiota Profiling in Obese and Non-Obese Patients: A Comparison of Shotgun Metagenomics and 16S rRNA Amplicon Sequencing
Source: Life (Basel). 2026 Sep 3;16(9):1474. doi: 10.3390/life16091474 (PMC13608661; doi:10.3390/life16091474)
Supplement: Supplementary file 1 [file life-16-01474-s001.zip › Supplementary Table S2 Demographics .pdf]

Supplementary Table S2. Demographics

	Study Group (n 32)	Control Group (n32)	p value
Mean age	56 (33-70)	53,2 (19-70)	
Sex	7 M-25 F	12 M -20 F	0.453
Mean BMI	35.4	23,4	<0.05
DMT2	10(26.3%)	3(7.89%)	0.364
Hypertension	21(55.2%)	17(44.7%)	0.499
Dyslipidemia	11(28.9%)	6 (15.7%)	0.407
Smoking	6 (15.7%)	8 (21%)	0.398
