# Supplementary material for: Bile Microbiota Profiling in Obese and Non-Obese Patients: A Comparison of Shotgun Metagenomics and 16S rRNA Amplicon Sequencing
Source: Life (Basel). 2026 Sep 3;16(9):1474. doi: 10.3390/life16091474 (PMC13608661; doi:10.3390/life16091474)
Supplement: Supplementary file 1 [file life-16-01474-s001.zip › Supplementary Table S3.pdf]

Supplementary Table S3. Taxonomic annotation of negative control samples analyzed by 16S rRNA amplicon sequencing

Sample	SampleB1	SampleB2	SampleB65
Candidatus Symbiobacter	0,1282	0,1309	0,2069
Burkholderia-Caballeronia-Paraburkholderia	0,1245	0,1007	0,1523
Candidatus Accumulibacter	0,0275	0,0279	0,0435
Microbacterium	0,0269	0,0227	0,0316
Sphingomonas	0,0193	0,0124	0,0128
Methanothermobacter	0,0162	0,0003	0,0000
Bacteroides	0,0152	0,0145	0,0000
Leifsonia	0,0149	0,0088	0,0224
Allorhizobium-Neorhizobium-Pararhizobium-Rhizobium	0,0146	0,0049	0,0031
Occallatibacter	0,0124	0,0007	0,0000
Alistipes	0,0120	0,0041	0,0000
Hyphomicrobium	0,0107	0,0038	0,0007
Family XIII AD3011 group	0,0107	0,0001	0,0000
SWB02	0,0104	0,0002	0,0000
Bauldia	0,0098	0,0114	0,0000
Brevundimonas	0,0095	0,0248	0,0123
Caulobacter	0,0082	0,0050	0,0081
Escherichia-Shigella	0,0079	0,0056	0,0029
Lactococcus	0,0078	0,0001	0,0000
Pseudomonas	0,0069	0,0147	0,0055
Thiomonas	0,0066	0,0002	0,0051
Methanobrevibacter	0,0065	0,0001	0,0000
Faecalibacterium	0,0063	0,0001	0,0064
Variovorax	0,0060	0,0004	0,0002
Levilactobacillus	0,0059	0,0089	0,0022
Parafrigoribacterium	0,0057	0,0045	0,0076
Nocardioides	0,0057	0,0057	0,0005
Agromyces	0,0056	0,0027	0,0053
Bifidobacterium	0,0055	0,0061	0,0000
Acidibacter	0,0054	0,0001	0,0000
Sinomonas	0,0053	0,0001	0,0000
Vicinamibacter	0,0051	0,0017	0,0010
Bacillus	0,0051	0,0005	0,0002
Phenylobacterium	0,0051	0,0030	0,0032
Lachnospiraceae UCG-008	0,0049	0,0072	0,0031
SH3-11	0,0048	0,0001	0,0000
Glutamicibacter	0,0048	0,0036	0,0055
Neochlamydia	0,0047	0,0001	0,0000
Anaerococcus	0,0046	0,0001	0,0000
YC-ZSS-LKJ147	0,0043	0,0001	0,0000
Uliginosibacterium	0,0043	0,0001	0,0000
Neisseria	0,0042	0,0027	0,0026
Sva0081 sediment group	0,0042	0,0001	0,0000
MND1	0,0041	0,0002	0,0003
Cutibacterium	0,0041	0,0016	0,0009
Candidatus Solibacter	0,0040	0,0001	0,0000
Ralstonia	0,0034	0,0033	0,0127
Opitutus	0,0032	0,0034	0,0000
Acinetobacter	0,0032	0,0025	0,0014
Bradyrhizobium	0,0032	0,0090	0,0087
Azonexus	0,0031	0,0037	0,0047
Galbitalea	0,0031	0,0021	0,0026
Mycetocola	0,0029	0,0019	0,0034
Leucobacter	0,0029	0,0019	0,0031

Promicromonospora	0,0029	0,0001	0,0001
Cnuibacter	0,0028	0,0035	0,0064
Lacunisphaera	0,0026	0,0001	0,0000
Achromobacter	0,0025	0,0028	0,0033
Staphylococcus	0,0024	0,0004	0,0006
Polynucleobacter	0,0023	0,0003	0,0005
Micromonospora	0,0023	0,0001	0,0001
Delftia	0,0022	0,0025	0,0033
Rothia	0,0021	0,0005	0,0023
Streptomyces	0,0020	0,0010	0,0007
Terrimicrobium	0,0020	0,0000	0,0000
Blautia	0,0019	0,0016	0,0003
Cryobacterium	0,0019	0,0012	0,0016
Limosilactobacillus	0,0018	0,0163	0,0082
Candidatus Limnoluna	0,0018	0,0009	0,0016
Agrococcus	0,0018	0,0010	0,0025
Diaminobutyricimonas	0,0017	0,0003	0,0022
Rivibacter	0,0016	0,0014	0,0026
Roseiarcus	0,0016	0,0004	0,0001
Corynebacterium	0,0016	0,0010	0,0010
Candidatus Planktoluna	0,0016	0,0008	0,0019
Enterobacter	0,0015	0,0014	0,0020
Dolosigranulum	0,0015	0,0000	0,0000
TM7a	0,0014	0,0000	0,0000
Salmonella	0,0014	0,0008	0,0001
Massilia	0,0014	0,0010	0,0014
Jatrophihabitans	0,0014	0,0034	0,0001
Akkermansia	0,0013	0,0000	0,0100
Lactiplantibacillus	0,0013	0,0033	0,0011
Lysinibacillus	0,0012	0,0007	0,0000
Rubrivivax	0,0012	0,0015	0,0015
Cupriavidus	0,0012	0,0010	0,0015
Proteiniclasticum	0,0012	0,0000	0,0000
Thiobacillus	0,0012	0,0013	0,0013
Conyzicola	0,0012	0,0005	0,0020
Subdoligranulum	0,0011	0,0000	0,0000
Craurococcus-Caldovatus	0,0011	0,0000	0,0000
Actinoplanes	0,0010	0,0000	0,0000
Limnobacter	0,0010	0,0011	0,0013
Okibacterium	0,0010	0,0106	0,0014
Pseudoclavibacter	0,0010	0,0008	0,0012
Sphingobium	0,0009	0,0010	0,0012
Latilactobacillus	0,0009	0,0095	0,0001
Curtobacterium	0,0008	0,0007	0,0012
Chryseoglobus	0,0008	0,0007	0,0023
Polaromonas	0,0008	0,0018	0,0013
Altererythrobacter	0,0008	0,0009	0,0011
[Ruminococcus] torques group	0,0008	0,0005	0,0073
Ochrobactrum	0,0008	0,0017	0,0006
Ellin6067	0,0008	0,0009	0,0028
Rathayibacter	0,0007	0,0006	0,0014
Ligilactobacillus	0,0007	0,0017	0,0001
Lachnospiraceae NK4A136 group	0,0006	0,0233	0,0204
Devosia	0,0005	0,0132	0,0002
Ensifer	0,0005	0,0010	0,0000

Lachnoclostridium	0,0005	0,0018	0,0002
Stenotrophomonas	0,0005	0,0059	0,0002
Lactobacillus	0,0004	0,0107	0,0006
Mesorhizobium	0,0004	0,0017	0,0001
Erythrobacter	0,0004	0,0015	0,0005
Cryocola	0,0004	0,0003	0,0016
Crossiella	0,0003	0,0024	0,0003
Hoeflea	0,0003	0,0013	0,0002
Lysobacter	0,0003	0,0013	0,0004
Methylobacterium-Methylorubrum	0,0002	0,0053	0,0038
Ruminococcus	0,0002	0,0049	0,0001
Hephaestia	0,0002	0,0001	0,0015
Pseudolabrys	0,0002	0,0013	0,0009
Paracoccus	0,0001	0,0004	0,0048
Actinomyces	0,0001	0,0001	0,0067
Rhodococcus	0,0001	0,0000	0,0075
Oscillibacter	0,0001	0,0047	0,0000
Garicola	0,0001	0,0018	0,0000
Monoglobus	0,0001	0,0135	0,0000
Herbaspirillum	0,0001	0,0000	0,0031
Gemmata	0,0000	0,0044	0,0000
Arenimonas	0,0000	0,0058	0,0001
Butyricicoccus	0,0000	0,0000	0,0015
Luteimonas	0,0000	0,0000	0,0018
Muribaculum	0,0000	0,0357	0,0000
Rhizobacter	0,0000	0,0101	0,0000
Butyricimonas	0,0000	0,0000	0,0082
Rhodoblastus	0,0000	0,0122	0,0000
Parabacteroides	0,0000	0,0028	0,0001
Conexibacter	0,0000	0,0000	0,0069
Paenibacillus	0,0000	0,0044	0,0000
Luteitalea	0,0000	0,0021	0,0021
Subgroup 10	0,0000	0,0074	0,0000
Terrabacter	0,0000	0,0063	0,0000
Candidatus Renichlamydia	0,0000	0,0038	0,0000
Tepidisphaera	0,0000	0,0032	0,0000
Ohtaekwangia	0,0000	0,0029	0,0000
Bounagaea	0,0000	0,0029	0,0000
Defluviimonas	0,0000	0,0020	0,0000
Planococcus	0,0000	0,0016	0,0000
Planomicrobium	0,0000	0,0011	0,0000
Flavisolibacter	0,0000	0,0000	0,0013
Planctomicrobium	0,0000	0,0011	0,0000
