# Supplementary material for: Bile Microbiota Profiling in Obese and Non-Obese Patients: A Comparison of Shotgun Metagenomics and 16S rRNA Amplicon Sequencing
Source: Life (Basel). 2026 Sep 3;16(9):1474. doi: 10.3390/life16091474 (PMC13608661; doi:10.3390/life16091474)
Supplement: Supplementary file 1 [file life-16-01474-s001.zip › Supplementary Table S5.pdf]

Sample	sample11	Sample37	Sample47	sample15
Escherichia-Shigella	0,10281794	0,001214148	0,00015266	0,3907737
Salmonella	0,08519077	0,002306664	0,00013621	0,0177186
Bacillus	0,000060634	0,002285487	0,00045797	0,0020812
Burkholderia-Caballeronia-Paraburkholderi	0,000424438	0,006805419	0,00961206	0,000246
Candidatus Symbiobacter	0,000268522	0,00718932	0,01195718	6,622E-05
Listeria	0	0,000261183	0,00005593	6,622E-05
Streptococcus	0,000069296	0,000143352	0,00012831	0,0041151
Levilactobacillus	0,000017324	0,000181362	6,4484E-05	5,676E-05
Enterobacter	0,16747977	0,00024435	9,6068E-05	0,0234986
Staphylococcus	0,000034648	0,000855768	0,00052179	0,0001987
Klebsiella	0,171221754	0,000177018	4,8034E-05	0,0085235
Haemophilus	0,0047641	0,000139008	0,00048955	0,0050895
Lachnospiraceae UCG-008	0,00017324	0,000408879	0,00034545	0,0531084
Pseudomonas	0,000164578	0,001572528	0,00257804	0,0031218
Lactiplantibacillus	0,000008662	0,000033666	3,2242E-05	0,0001514
Candidatus Accumulibacter	0,000077958	0,00162357	0,00219246	5,676E-05
Limosilactobacillus	0	0,000084165	0,00011252	0,0001797
Lactobacillus	0,00008662	0,000029322	3,2242E-05	0,000823
Microbacterium	0,000051972	0,001404198	0,00219246	5,676E-05
Lacticaseibacillus	0,000077958	0	0,00005593	7,568E-05
Fusobacterium	0,000025986	0	0	0,1506883
Enterococcus	0,000008662	0,000062988	8,8172E-05	0,005203
Bacteroides	0,000684298	0,000425712	0,00021714	0,0005865
Faecalibacterium	0,000831552	0,000021177	1,5792E-05	0,0005487
Clostridium sensu stricto 1	0,00004331	0,000004344	0	0,1265086
Sphingomonas	0,000121268	0,000594585	0,00094752	7,568E-05
Pantoea	0,038831746	0,000097197	4,0138E-05	0,0160158
Serratia	0,012525252	0	0	0,0188632
Leifsonia	0,000112606	0,000569064	0,00056193	2,838E-05
Citrobacter	0,021854226	0,000016833	3,2242E-05	0,0067734
Candidatus Stammerula	0,0809897	0	0	0
Brevundimonas	0	0,000974142	0,00110807	0
Lachnospiraceae NK4A136 group	0,000277184	0,000004344	1,5792E-05	0,0012393
Bifidobacterium	0,000121268	0,000413223	7,896E-06	5,676E-05
Akkermansia	0,000060634	0,000155841	0	0,0006244
Erwinia	0,055419476	0,000008688	0	0,0006527
Blautia	0,000164578	0,000033666	0,00007238	0,0036137
Ralstonia	0,000017324	0,000278559	0,00017634	3,784E-05
Yersinia	0,010515668	0,000016833	0	0,0105101
Aeromonas	0,008722634	0,000822102	0,00048166	0,006849
Bradyrhizobium	0,000017324	0,000177018	0,00025728	9,46E-06
Ruminococcus	0,000103944	0,000004344	0	0,0002554
Caulobacter	0,000017324	0,000590241	0,00231287	9,46E-06
Latilactobacillus	0,000008662	0,000071676	0,00016055	0,0004919
Muribaculum	0	0	0	0
Allorhizobium-Neorhizobium-Pararhizobium	0,000017324	0,000413223	8,0276E-05	1,892E-05
Bryobacter	0,000008662	0	0	1,892E-05
Alistipes	0,000095282	0,000029322	0,00053824	6,622E-05
Kluyvera	0,01762717	0,000008688	0	0,0001324
Ligilactobacillus	0	0,000021177	0	0,0001419
Parafrigoribacterium	0,000034648	0,000240549	0,00082711	9,46E-06
Catenibacterium	0	0	0,00053824	2,838E-05
Achromobacter	0,000017324	0,000236205	0,00020859	0
Delftia	0,000025986	0,000210684	0,00016845	0,0000473

Anoxybacillus	0	0	0,00007238	0,0002176
Cronobacter	0,00809897	0,000025521	2,4346E-05	0,0039732
Subdoligranulum	0,000181902	0,000008688	0	0,0000946
Neisseria	0,00004331	0,000105342	0,0001849	0,0001987
Glutamicibacter	0,000034648	0,000236205	0,00025728	9,46E-06
Dialister	0,000095282	0	0	9,46E-06
Roseobacter clade NAC11-7 lineage	0,00008662	0,001800588	0,0015259	6,622E-05
Azonexus	0,000017324	0,000286704	0,00039348	2,838E-05
Methylobacterium-Methylorubrum	0,000008662	0	1,5792E-05	0
Geobacillus	0	0	0	3,784E-05
Stenotrophomonas	0,000051972	0,000860112	0,00077907	0,0000473
Kosakonia	0,012984338	0,000029322	0	0,0006811
Acinetobacter	0,000034648	0,000345891	0,00033755	0,0002081
Nocardioides	0	0,000097197	0,00005593	0
Corynebacterium	0	0,000046155	0,00023293	0,0000946
Vibrio	0,004010506	0,000088509	0,00017634	0,0019677
[Ruminococcus] torques group	0,000051972	0	0	0,0001324
Enterobacillus	0,00368135	0	0	3,784E-05
Prevotella_9	0,000181902	0,000231861	0,00056983	7,568E-05
Agromyces	0,000017324	0,000097197	0,00039348	9,46E-06
Cutibacterium	0,000008662	0,000046155	8,8172E-05	8,514E-05
Paracoccus	0,000008662	0,000008688	0	0,0000473
Franconibacter	0,006098048	0,000004344	0	0,0029799
Lachnoclostridium	0,000095282	0,000265527	0	0,0016366
Lysinibacillus	0	0,000105342	0,00007238	8,514E-05
Pediococcus	0	0	0	3,784E-05
Proteus	0,00077958	0	0	0,0043611
Lactococcus	0	0,000004344	0	0,0003216
Agathobacter	0,000103944	0	0	0,0036232
Mycetocola	0	0,000046155	9,6068E-05	0
Bauldia	0	0	0	0
Truepera	0	0	0	0
Roseburia	0,000051972	0	1,5792E-05	0,0014568
Devosia	0	0	0	0
Phenylobacterium	0	0,000400734	0,00044941	0
Aquabacterium	0,000034648	0,000033666	4,8034E-05	0,0000473
Prevotella	0,000155916	0	2,4346E-05	0,000246
Virgibacillus	0	0	0	8,514E-05
Phascolarctobacterium	0,000051972	0	0	0,0000946
Monoglobus	0	0,000004344	0	0,0028758
Streptomyces	0,000034648	0,00003801	3,2242E-05	5,676E-05
Lentilactobacillus	0	0	0	0,0001135
Hyphomicrobium	0	0,000189507	1,5792E-05	0
Jeotgalibaca	0	0,000004344	0	0,0012771
Clade Ia	0	0,000008688	6,4484E-05	0
Cnuibacter	0	0	0	0
UCG-005	0,000164578	0,000004344	0	8,514E-05
Morganella	0,01312293	0	0	0,0004825
Christensenellaceae R-7 group	0,000311832	0,000004344	0	0,0017785
Oscillibacter	0,000017324	0	0	0,0001419
Musa	0	0,001260846	0,00100411	0
Eucalyptus	0,00004331	0,00096111	0,00113242	9,46E-06
RB41	0	0	0	0
Methanothermobacter	0	0	0	0
Veillonella	0,000034648	0	0	3,784E-05

Rhodococcus	0	0,000054843	7,896E-06	3,784E-05
Vicinamibacter	0	0	0	0
Ochrobactrum	0	0	0	0
Leucobacter	0	0,000139008	8,8172E-05	0
Massilia	0,000017324	0,000080364	0,00010462	9,46E-06
Galbitalea	0	0	0	0
Sodalis	0,006816994	0,000004344	7,896E-06	0,0008892
Porphyromonas	0,000051972	0,000016833	3,2242E-05	1,892E-05
[Eubacterium] hallii group	0,00004331	0	0	0,0000473
NK4A214 group	0,00004331	0,000400734	0	0,0002649
Saliterribacillus	0	0	0	0
Rhizobacter	0	0	0	0
Ellin6067	0,000008662	0,000050499	1,5792E-05	0
Rubrivivax	0,000008662	0,000067332	0,00016055	0
Okibacterium	0	0	0	0
Weissella	0	0,000012489	2,4346E-05	9,46E-06
Butyricimonas	0,000025986	0	0	9,46E-06
Agrococcus	0,000008662	0,00007602	0,00020069	0
Family XIII AD3011 group	0	0	0	0
Thiobacillus	0,000008662	0,000046155	0,00012041	0
Occallatibacter	0	0	0	0
Butyricicoccus	0,000034648	0,000004344	7,896E-06	6,622E-05
Spiroplasma	0	0	0	0
Thiomonas	0	0	0	0
Mycobacterium	0	0	7,896E-06	5,676E-05
Carnobacterium	0	0	0	0
Candidatus Nitrososphaera	0	0	0	0
Incertae Sedis	0	0	0	0,0010784
uncultured	0,000060634	0,000004344	7,896E-06	0
SWB02	0	0	0	0
Rhodoblastus	0	0	0	0
Parabacteroides	0,000051972	0	0	0,0000473
[Ruminococcus] gauvreauii group	0,000025986	0	0	0,0063571
Rivibacter	0	0,00020634	0,00014476	0
Conexibacter	0	0	0	0
Rothia	0	0,000025521	0,00010462	9,46E-06
Prevotella_7	0,000051972	0	4,0138E-05	9,46E-06
Pedobacter	0	0	0	0
Cupriavidus	0	0,000193851	2,4346E-05	0
Lachnospira	0,000008662	0	0	1,892E-05
Gemmata	0,000008662	0	0	0
Barnesiella	0,000077958	0	0	0
Raoultella	0,003300222	0	1,5792E-05	0,0007
Actinomyces	0	0,000021177	7,896E-06	0
Flavobacterium	0,000017324	0	3,2242E-05	0,0001041
Rhodoferax	0	0,000062988	6,4484E-05	0,0004068
CAG-873	0,00021655	0	0	0
Alloprevotella	0,000112606	0	0	0
Altererythrobacter	0	0	4,0138E-05	0
Variovorax	0	0,000042354	4,8034E-05	0
Polaromonas	0	0,000084165	0,00013621	0
Holdemanella	0,000008662	0	0	0,0001041
Mesorhizobium	0	0	0	0
Cryobacterium	0	0,000126519	0	0
Enhydrobacter	0,000017324	0,000016833	8,8172E-05	1,892E-05

Pseudoalteromonas	0,001281976	0,000021177	4,8034E-05	0,00035
Curtobacterium	0	0,000046155	0,00005593	0
Granulicatella	0	0,000004344	0	0,0003784
Buttiauxella	0,000069296	0	0	9,46E-06
Sphingobium	0	0,000117831	0,00015266	0
Paenibacillus	0	0	0,00020859	5,676E-05
Methanobrevibacter	0,000008662	0,000021177	7,896E-06	0,0000473
Paucilactobacillus	0	0	0	0
Limnobacter	0,000025986	0,000050499	8,8172E-05	0
Dorea	0,000025986	0,000021177	0	9,46E-06
Ensifer	0	0	0	0
Arthrobacter	0,000008662	0,000029322	9,6068E-05	0
Leptotrichia	0	0	0	9,46E-06
Comamonas	0	0,000067332	3,2242E-05	9,46E-06
Candidatus Planktoluna	0	0,000029322	0,00005593	0
Diaphorobacter	0,000008662	0,000012489	6,4484E-05	1,892E-05
Luteitalea	0	0	0	0
Candidatus Udaeobacter	0	0	0	9,46E-06
Brochothrix	0	0	0	0
Subgroup 23	0	0	0	0
Subgroup 10	0	0	0	5,676E-05
Porphyrobacter	0	0	4,8034E-05	0
Erysipelotrichaceae UCG-003	0,000025986	0	0,000329	9,46E-06
UCG-002	0,000051972	0,000008688	0	9,46E-06
Sulfuritalea	0,000008662	0,000004344	7,896E-06	2,838E-05
Skermanella	0	0	0	0
Alcaligenes	0	0,00003801	4,8034E-05	5,676E-05
Novosphingobium	0	0,00003801	9,6068E-05	0
Candidatus Solibacter	0	0,000016833	0	9,46E-06
Jatrophihabitans	0	0	7,896E-06	0
Opitutus	0	0	0	0
Pseudonocardia	0,000008662	0,000198195	0,00011252	2,838E-05
CAG-352	0	0	0	0
Pseudogracilibacillus	0	0,000016833	0	0
Desulfitibacter	0	0	0	0
Anaerostipes	0,000025986	0,000021177	0	0,0000946
Gaiella	0	0	0	0
Imtechella	0	0	0	0
Pseudolabrys	0	0,000008688	0,00007238	0
Chryseoglobus	0	0	0,00030531	0
Anaerococcus	0,000008662	0	0	2,838E-05
Sideroxydans	0,000017324	0,000025521	0,00007238	0
Conyzicola	0	0,000134664	0,00012831	0
Pseudoclavibacter	0	0	0,00012041	0
Rubrobacter	0	0,000008688	0	0
Lysobacter	0	0,000021177	2,4346E-05	2,838E-05
Candidatus Limnoluna	0	0	0	0
Providencia	0,003819942	0	0	0,0004446
Arenimonas	0	0,000004344	7,896E-06	0
Coprococcus	0,00004331	0,000016833	0,00005593	8,514E-05
Lautropia	0	0,000029322	8,0276E-05	1,892E-05
Actinobacillus	0,000675636	0	0	0,0000946
Terrabacter	0	0,000025521	0	0
Gilliamella	0,000051972	0	0	3,784E-05
Rathayibacter	0	0	0	0

Polynucleobacter	0	0,000029322	4,0138E-05	0
Lachnospiraceae NK3A20 group	0,000034648	0,000004344	7,896E-06	0,0002554
MND1	0	0,000016833	7,896E-06	0
Acidibacter	0	0	0	0
Succinivibrio	0,000485072	0	0	0,0001135
Apilactobacillus	0	0	0	0
Diaminobutyricimonas	0	0	0	0
Zhihengliuella	0	0,000016833	1,5792E-05	0
Erythrobacter	0	0	0	0
Tenuibacillus	0	0	0	0
Fusicatenibacter	0,000008662	0,000008688	0	0,0000473
Selenomonas	0,000017324	0	0	9,46E-06
Leuconostoc	0,000008662	0	0	9,46E-06
Neochlamydia	0	0	0	0
Microvirga	0	0	0	0
Sinomonas	0	0	0	0
Pedomicrobium	0	0	0	0
Gracilibacillus	0	0	0	0
Sarcina	0	0	0	0,0049192
Pirellula	0	0	0	0
Turicibacter	0	0	0,00042573	9,46E-06
Crossiella	0	0,000021177	0	0
Zoogloea	0	0	0	1,892E-05
Kingella	0	0,000012489	1,5792E-05	0
Gallionella	0	0,000012489	4,8034E-05	0
Granulicella	0	0	0	0
Hoeflea	0	0	0	0
Candidatus Endoecteinascidia	0	0	0	0,0017406
SH3-11	0	0	0	0
Micrococcus	0	0,000004344	7,896E-06	0
Nitratireductor	0	0	0	0
Rahnella1	0,000744932	0,00015204	0	0,0004635
Planifilum	0	0	0	0
Silvimonas	0	0,000016833	4,8034E-05	0
Companilactobacillus	0	0	0	0
Shewanella	0,000814228	0,000029322	7,896E-06	0,0003784
Thiomicrorhabdus	0,000017324	0,000295392	0,00028097	3,784E-05
Marinobacter	0	0,000071676	0,00015266	0,0000473
Cibicidoides	0,000025986	0,000291048	0,00015266	9,46E-06
Haliangium	0	0	0	0
Lactovum	0	0,000004344	0	0,0002554
Promicromonospora	0	0	7,896E-06	0
Alteromonas	0,000615002	0,000008688	0	0,0006149
Rikenellaceae RC9 gut group	0,000077958	0	0,00024872	1,892E-05
YC-ZSS-LKJ147	0	0,000004344	0	0
Lachnospiraceae UCG-001	0,000008662	0	2,4346E-05	0,0004446
Silanimonas	0	0,000016833	0,00073104	0
Uliginosibacterium	0	0	0	0
Plantibacter	0	0	0	9,46E-06
Gemmatimonas	0	0,000054843	0	0
Sva0081 sediment group	0	0	0	0
Colidextribacter	0,00004331	0,000004344	0	9,46E-06
Oceanobacillus	0	0,000042354	0	0
Parasutterella	0	0	0	1,892E-05
Solibacillus	0	0,000004344	4,8034E-05	2,838E-05

Sphingopyxis	0	0,000084165	4,8034E-05	0
Noviherbaspirillum	0	0,000008688	0,00005593	0
TM7a	0	0	0	1,892E-05
Herbaspirillum	0	0	0	9,46E-06
Humibacter	0	0	0	9,46E-06
Dokdonella	0	0	0	0
Defluviicoccus	0	0	0	0
[Ruminococcus] gnavus group	0,000025986	0,000008688	2,4346E-05	3,784E-05
Romboutsia	0	0	0	0,0002365
Extensimonas	0,000008662	0,000008688	7,896E-06	2,838E-05
Afipia	0	0	0	0
Collinsella	0,000008662	0	0	1,892E-05
Helicobacter	0	0	0	0
Candidatus Renichlamydia	0	0	0	0
Psychrobacter	0	0,000004344	0	0,0003122
Roseiarcus	0	0	7,896E-06	0
Prevotellaceae NK3B31 group	0,000017324	0	0	0,0001703
Prevotellaceae UCG-001	0,000008662	0	0	1,892E-05
Sphingosinicella	0	0	0	0
Gemella	0	0	0	1,892E-05
Zymomonas	0,000008662	0,000004344	7,896E-06	9,46E-06
Hydrogenophaga	0	0,000016833	0	2,838E-05
Bordetella	0	0	0	0
Bartonella	0	0	0	0
Moraxella	0	0	0	7,568E-05
Alsobacter	0	0	0	0
Lachnospiraceae UCG-004	0,000008662	0	0	0
Iamia	0	0	0	0
Janthinobacterium	0	0,000004344	0	0
Tyzzerella	0,000008662	0	2,4346E-05	2,838E-05
Tepidisphaera	0	0	7,896E-06	0
Rhodobacter	0	0	0	0
Fimbriiglobus	0	0	0	0
Micromonospora	0,000008662	0,000122175	0	0
Rhodopseudomonas	0	0	8,8172E-05	0
Pandoraea	0	0,000029322	8,8172E-05	0
Hephaestia	0	0	0	0
Izimaplasma	0	0	0	9,46E-06
Pseudacidovorax	0,000008662	0,000029322	2,4346E-05	0
Synechococcus CC9902	0	0,000046155	1,5792E-05	0,0000473
Acidovorax	0,000008662	0	1,5792E-05	1,892E-05
Nitrospira	0	0	0	0
Ohtaekwangia	0	0	0	0
Bounagaea	0	0	0	0
Caminicella	0	0	0	3,784E-05
Aminobacter	0	0	0	9,46E-06
Undibacterium	0	0	0	0
Propionivibrio	0	0,000004344	0	0
Andreprevotia	0,000008662	0	0	0
Enterorhabdus	0	0	0	9,46E-06
Ellin6055	0	0,000021177	0	0
Lacunisphaera	0	0	0	0
Umezawaea	0	0	0	0
Brevibacillus	0	0	0	0,0000473
Pygmaiobacter	0	0	0	0,0022515

Pajaroellobacter	0	0	0	0
Snodgrassella	0	0	0	0
Fructilactobacillus	0	0	0	0
Loigolactobacillus	0	0	0	2,838E-05
Amylolactobacillus	0	0	0	0
Ideonella	0	0,000025521	0	0
Halobacillus	0	0,000067332	0	0
Candidatus Methanomethylophilus	0,000077958	0	0	7,568E-05
Janibacter	0	0,000012489	7,896E-06	0
Dolosigranulum	0	0	0	1,892E-05
Chitinilyticum	0	0,000042354	0,00005593	0
Bagnisiella	0	0	0	0
alphaI cluster	0	0,000008688	1,5792E-05	0
Nitrosospira	0	0	0	0
Bergeyella	0	0	0	0
Rhodoplanes	0	0,000016833	0	0
Plesiomonas	0,000528382	0	0	0,0003122
Pir4 lineage	0	0,000004344	0	0
Cryocola	0	0	0	0
Puia	0	0	0	0
Actinomadura	0	0	0	0
Secundilactobacillus	0	0	0	0
Roseivivax	0	0,000016833	0,00010462	0
Pseudaminobacter	0	0	0	0
Cladosporium	0	0	0	0
Brassica	0,000008662	0,000168873	0,00012041	0
ML602J-51	0	0	0	0
Photobacterium	0,000415776	0	7,896E-06	0,0002554
[Eubacterium] siraeum group	0	0	0	0
Aurantimicrobium	0	0	8,0276E-05	0
Cellulomonas	0	0,000012489	7,896E-06	0
Brevibacterium	0	0	1,5792E-05	0
Luteimonas	0	0,000029322	0	0
[Eubacterium] eligens group	0,000008662	0	0	0
Clavibacter	0	0,000059187	0	0
Cerasibacillus	0	0	0	0
Pseudochrobactrum	0	0,000008688	0	0
Methanolobus	0	0	0	0
Garicola	0	0,000004344	7,896E-06	0
Halomonas	0,000008662	0	0	0,000123
Phyllobacterium	0	0	0	0
hgcI clade	0	0	0	0
Falsirhodobacter	0	0	0	0
Lysinimonas	0	0	0	0
UBA1819	0,000034648	0	0	0,0004541
Defluviimonas	0	0,000025521	0	0
Salinivibrio	0,001377258	0	0	8,514E-05
Lachnospiraceae UCG-006	0	0	0	0,0000473
Trichococcus	0	0	0	1,892E-05
Luteolibacter	0	0	0,00024083	9,46E-06
Prochlorococcus MIT9313	0	0	0	0
Candidatus Alysiosphaera	0	0	0	0
Microvirgula	0	0,000008688	1,5792E-05	0
Frankia	0	0,000008688	0	0
Falsochrobactrum	0	0	0	0

Candidatus Saccharimonas	0,000025986	0	0	0
OLB15	0	0	0	0
Rubellimicrobium	0	0,000071676	0	0
Methylibium	0	0	0	0
Shimwellia	0,00004331	0	0	0,000123
Planococcus	0	0	4,0138E-05	0
Terrimicrobium	0	0	0	0
Acetobacter	0	0	0	0
Piscicoccus	0	0,000008688	4,0138E-05	0
Actinomycetospora	0	0	0	0
UCG-003	0	0	0	0
Azoarcus	0	0	0	0
Candidatus Aquiluna	0	0,00007602	0	0
Psychromonas	0,000424438	0,000004344	0	0,0002081
Acetitomaculum	0,000008662	0	0	2,838E-05
Pectobacterium	0,00030317	0	0	0,0003027
Pseudarthrobacter	0	0	7,896E-06	0
Wolbachia	0	0	0	0
Lelliottia	0,000935496	0	0	6,622E-05
Dechloromonas	0	0,000004344	0	0
Acidothermus	0	0	0	0
Robbsia	0	0,000008688	1,5792E-05	0
Coxiella	0	0,000029322	7,896E-06	3,784E-05
Ipomoea	0	0,000274215	0	0
Scytonema UTEX 2349	0,000008662	0	0	0
Salinibacter	0	0	0	9,46E-06
DSSD61	0	0	0	0
Cryptomonas	0	0,000012489	6,4484E-05	9,46E-06
Limnochorda	0	0	0	0
Erysipelatoclostridium	0	0	0	2,838E-05
Alcanivorax	0	0	0	0,0002933
Peptoniphilus	0	0	0	9,46E-06
Acidaminococcus	0	0	0	0,0006054
Cm1-21	0	0	7,896E-06	0
Epibacterium	0	0,000286704	0,00015266	0
Lysinimicrobium	0	0	0	0
Rubritepida	0	0	0	0
Lachnospiraceae NC2004 group	0,000008662	0	0	0,0001514
Otariodibacter	0,00047641	0	0	0,0001135
Clostridium sensu stricto 12	0	0	0	0,0013149
Salinibacterium	0	0	0	0
Modestobacter	0	0,000004344	8,0276E-05	0
PMMR1	0	0,000046155	0	0
Pseudarcicella	0	0	0	0
Roseateles	0	0	0	0
Brucella	0	0	0	0
[Eubacterium] oxidoreducens group	0	0	0	1,892E-05
Ruminiclostridium	0	0	0	6,622E-05
Oribacterium	0,000008662	0	0	5,676E-05
Hevea	0	0,000198195	0,00011252	0
Planomicrobium	0	0	4,0138E-05	1,892E-05
OM60(NOR5) clade	0	0	0	0,0000473
Endozoicomonas	0,000025986	0,000004344	0	0,0004446
Syncephalastrum	0	0	0	0
Marvinbryantia	0,000017324	0	0	9,46E-06

Candidatus Phytoplasma	0	0	0	9,46E-06
Nautella	0	0	0	0
Anaerobacillus	0	0,00003801	0	0
Desulfovibrio	0,000008662	0	0	0,0001419
Sphingorhabdus	0,000008662	0	0	0
Candidatus Zinderia	0	0,000008688	7,896E-06	0
EUB33-2	0	0	0	1,892E-05
Ellin517	0	0	0	0
BD1-7 clade	0	0	4,8034E-05	0,0006527
JGI 0001001-H03	0	0	0	0
SM1A02	0	0	0	0
Desulfuromonas	0	0	0	0
Nordella	0	0	0	0
Ornithinibacillus	0	0	0	0
Lachnospiraceae FCS020 group	0	0	0	0,0005771
Thermacetogenium	0	0	0	0
Methyloversatilis	0	0,000008688	0	0
Azospira	0	0,000004344	1,5792E-05	0
Vitellibacter	0	0	0	0
Flavisolibacter	0	0	0	0
Azotobacter	0	0,000008688	4,8034E-05	0
Timonella	0	0,000008688	1,5792E-05	0
Chthoniobacter	0	0,000004344	8,8172E-05	0
Asanoa	0	0	0	0
Mitsuokella	0	0	0	0
[Eubacterium] ventriosum group	0,000017324	0,000004344	0	0
Catenovulum	0,00134261	0	0	0
Candidatus Haloredivivus	0	0	0	0
Lachnospiraceae XPB1014 group	0	0	0	9,46E-06
Megasphaera	0,000034648	0	0	9,46E-06
Sanguibacter-Flavimobilis	0	0,000012489	2,4346E-05	0
Odoribacter	0,000008662	0,000004344	0	0
Abiotrophia	0	0	0	0,0001608
Peptoclostridium	0	0	0	0,000596
Arachis	0	0,000097197	0,00028886	0
Clade Ib	0	0	0	0
Albimonas	0	0	0	0
Ferribacterium	0	0,000008688	4,0138E-05	0
Oleiphilus	0,000008662	0	0	0,0003406
Brachymonas	0	0,000008688	1,5792E-05	1,892E-05
Rhodanobacter	0	0,000004344	0	0
Caproiciproducens	0	0	0	0
Gemmobacter	0	0	0	0
Exiguobacterium	0	0	2,4346E-05	0
UCG-009	0	0	0	0,0003406
Oceanisphaera	0,00017324	0	0	0,0001892
Parablastomonas	0	0	0	0
Edaphobaculum	0	0	0	0
Shinella	0	0	0	0
GCA-900066575	0	0	0	6,622E-05
Proteiniclasticum	0	0	0	2,838E-05
OM27 clade	0	0	0	0
Candidatus Entotheonella	0	0	0	0
Craurococcus-Caldovatus	0	0	0	0
Phaseolibacter	0,000242536	0	0	0,0000946

Pilimelia	0	0	0	0
Aeromicrobium	0	0	0	0
Ferrovum	0,000008662	0,000004344	1,5792E-05	0
Pseudooceanicola	0	0	0	0
Planctomicrobium	0	0	0	0
Actinoplanes	0	0	0	0
Lachnospiraceae AC2044 group	0,000025986	0	0	0,0001703
Lachnospiraceae ND3007 group	0,000017324	0	0	1,892E-05
Arsenophonus	0,000242536	0	0	0,000123
Aureimonas	0	0	0	0
Pseudorhodoplanes	0	0	0	0
Capnocytophaga	0	0	0	0
Atopostipes	0	0	2,4346E-05	0
Z-35	0	0,000004344	0	0
Haloquadratum	0	0	0	0
Mycosphaerella	0	0,000025521	0	0
Turicella	0	0	0	0
Methylomonas	0	0	0	0,0003689
Roseomonas	0	0	0	0
Cellulosimicrobium	0	0	7,896E-06	0
Acanthamoeba	0	0,00003801	2,4346E-05	0
Steroidobacter	0	0	0	0
Dothidea	0	0	0	0
YC-ZSS-LKJ63	0	0	0	0
Parasphingopyxis	0	0,000008688	1,5792E-05	0
Planktomarina	0	0	0	0
Curvibacter	0,000034648	0	0	0
Pelagibacterium	0	0,000008688	0	0
Sandarakinorhabdus	0	0	0,00106793	0
Furfurilactobacillus	0	0	0	0
Dermabacter	0	0	0	0
Butyrivibrio	0,000017324	0	0	9,46E-06
Pseudobutyrivibrio	0	0	0	0,0002838
Scleromitrula	0	0	0	0
Lawsonella	0	0,000004344	0	1,892E-05
Acidiphilium	0	0	0	0
Sulfurovum	0	0	0	1,892E-05
Microlunatus	0	0	0	9,46E-06
[Eubacterium] xylanophilum group	0	0	0	9,46E-06
Dickeya	0,000155916	0	0	0,0001041
Kurthia	0	0,000067332	8,8172E-05	0
Thauera	0	0	7,896E-06	0,000123
Kocuria	0	0	7,896E-06	9,46E-06
Planctopirus	0	0	0	0
Salinicoccus	0	0	0	0
Nitrosomonas	0	0	0	0
Sulfurimonas	0	0	0	9,46E-06
Oxalobacter	0	0,000004344	7,896E-06	0
Oscillatoria SAG 1459-8	0	0	0	0
Ezakiella	0	0	0	9,46E-06
Sutterella	0,000008662	0,000004344	7,896E-06	0
Gibbsiella	0,000025986	0	0	0,0001324
Solanum	0	0,000139008	0,00024872	0
Clostridium sensu stricto 13	0	0	0	0,0005014
Xanthobacter	0	0	0	0

Succinivibrionaceae UCG-001	0,000008662	0	0	0
Stappia	0	0	0	0
Nitrobacter	0	0	0	0
Izhakiella	0,000008662	0	0	0,0001892
ANME-3	0	0,000021177	4,8034E-05	0
Denitratisoma	0	0	0	0
Edwardsiella	0,000181902	0	0	7,568E-05
Campylobacter	0	0	0	0
Immundisolibacter	0	0	0	3,784E-05
Candidatus Stoquefichus	0	0	0	0
CENA359	0	0	0	0
Roseimicrobium	0	0,000059187	2,4346E-05	9,46E-06
Treponema	0,000034648	0	0	1,892E-05
Deinococcus	0	0	0	9,46E-06
Brachybacterium	0	0,000004344	0	0
Fournierella	0	0	0	9,46E-06
Rickettsia	0	0,00007602	0	0
Fictibacillus	0	0	0	0
SZB85	0	0	0	0
Family XIII UCG-001	0	0	0	0,0007
RS62 marine group	0	0	1,5792E-05	0
Klenkia	0	0	0	0
Buchnera	0,000363804	0	0	5,676E-05
Shuttleworthia	0	0	0	0,0001324
Colwellia	0,000025986	0	0	1,892E-05
Desulfotignum	0	0	0	0
Sulfitobacter	0	0	0	0
Bombilactobacillus	0	0	0	0
Hafnia-Obesumbacterium	0,000207888	0	0	7,568E-05
Anaerocolumna	0	0	0	0
Sulfuriferula	0,000034648	0	0	0
NS4 marine group	0	0,000004344	7,896E-06	0
Candidatus Tremblaya	0	0,000008688	0	0
Pseudocitrobacter	0	0	0	0
Catenulispora	0	0	0	0
Ruminobacter	0,000095282	0	0	7,568E-05
Commensalibacter	0	0	0	0
Labrenzia	0	0,000021177	0	0
Asticcacaulis	0	0	0	0
Schumannella	0	0	0	0
Methylorosula	0,000008662	0	0	5,676E-05
Cedecea	0,000450424	0	0	1,892E-05
Sphingobacterium	0	0,000004344	0	0
Erythranthe	0	0,000185706	0	0
Kaistia	0	0	0	0
Pusillimonas	0	0	0	0
Desulfohalotomaculum	0	0	0	9,46E-06
Clostridium sensu stricto 14	0	0	0	0,0006527
Methylocystis	0	0	0	0
Nocardia	0	0	7,896E-06	0
Solirubrobacter	0	0	0	0
Sporosarcina	0	0	0,00010462	0
Anaerosporobacter	0	0	2,4346E-05	0
Prevotellaceae UCG-003	0,000008662	0	0	9,46E-06
Dendrosporobacter	0	0,000004344	0	0

Gallicola	0	0	0	9,46E-06
Rhodopirellula	0	0	7,896E-06	0
Paenarthrobacter	0	0	0	0
mle1-7	0	0	0	0
Nocardiopsis	0	0	0	0
Bacillicladium	0	0	0	0
p-1088-a5 gut group	0	0	0	0
Gossypium	0	0	0,00057838	0
Mameliella	0	0	0	0
Ottowia	0	0	0	0
Petrotoga	0	0	0	0
A2	0	0	0	0,0002933
Reyranella	0	0,000004344	1,5792E-05	0
Blastococcus	0	0	0	0
C39	0	0,000004344	1,5792E-05	0
Bosea	0	0	0	0
Alkalibacterium	0	0	0,00056983	0
Tetrasphaera	0	0	0	0
Saccharofermentans	0	0	0	2,838E-05
Salimicrobium	0	0	0	6,622E-05
Ramlibacter	0	0	0	0
Pluralibacter	0,000051972	0	0	9,46E-06
Thermoactinomyces	0	0	0	0
Halolamina	0	0,000008688	1,5792E-05	0
Parviterribacter	0	0	0	0
endosymbionts	0,00017324	0	0	0,0000946
Roseovarius	0	0,000016833	3,2242E-05	0
Fusarium	0	0	0	0
Marinobacterium	0	0,000004344	0	5,676E-05
Lachnospiraceae UCG-010	0	0	0	0
Thalassobius	0	0	0	0
Quadrisphaera	0	0,000004344	2,4346E-05	0
Trametes	0	0	0	0
Allisonella	0	0	0	3,784E-05
Yoonia-Loktanella	0	0	0	0
Tenacibaculum	0	0	0	0
Finegoldia	0	0	0	0
Lutispora	0	0	0	0,0004257
Clostridium sensu stricto 11	0	0	0	0,0004825
UCG-008	0	0	0	0
Polycyclovorans	0	0	0	0
Pyrenochaeta	0	0	0	0
Peredibacter	0	0	0	0
Stereum	0	0	0	0
Chroococcidiopsis PCC 7203	0	0	0	0
Dyadobacter	0	0	0	0
Methylotenera	0	0,000004344	0	0
Ascidiaceihabitans	0	0	0	0
Mucilaginibacter	0	0	0	0
Domibacillus	0	0	0	1,892E-05
Aerococcus	0	0	0	9,46E-06
Bhargavaea	0	0	0	0
Edaphobacter	0	0	0	0
Flavihumibacter	0	0	0	0
Nitrospirillum	0	0	0	0

Candidatus Cryptoprodotis	0	0	1,5792E-05	0
Candidatus Soleaferrea	0	0	0	0,0000473
Gryllotalpicola	0	0	0	0
Faecalibaculum	0	0	0	9,46E-06
Natronomonas	0	0	0	0
Demequina	0	0	0	0
Talaromyces	0,000008662	0	0	0
Tuzzerella	0	0	0	9,46E-06
Kitasatospora	0	0	7,896E-06	0
Craterocolla	0	0	0	0
Shimia	0	0	0	0
Vulcanibacillus	0	0	0	0
Isoptericola	0	0	0	0
IS-44	0	0	0	0
Olsenella	0	0	0	0
Hordeum	0	0,000025521	0	0
Flaviaesturariibacter	0	0	0	0
FukuN57	0	0	0	0
Thiohalocapsa	0,000008662	0	0	3,784E-05
Limnohabitans	0	0	0	0
Cyanobium PCC-6307	0	0	0	0
Nostoc SAG 60.79	0	0	0	0
Boseongicola	0	0	0	0
Hyphococcus	0	0	0	0
Methyloferula	0	0	0	0
Globicatella	0	0,000008688	0	0
Methanosphaera	0	0	0	0
Intestinibacter	0	0	0	0,0001608
Fastidiosipila	0	0	0	0,0001419
Thaumasiovibrio	0,000372466	0	0	0
Maritimibacter	0	0	0	0
Aquaspirillum	0	0	0	0
Psychrobacillus	0	0	0	9,46E-06
Clostridioides	0	0	7,896E-06	7,568E-05
Quatrionicoccus	0	0,000012489	0	0
Tannerella	0	0	0	0
GCA-900066755	0	0	0,00041783	0
Fusibacter	0	0	0	5,676E-05
Acetanaerobacterium	0	0	0	0,0003689
Halochromatium	0	0,000004344	0	9,46E-06
Photorhabdus	0,000138592	0	0	5,676E-05
Gordonia	0	0	0	0
Elusimicrobium	0	0	0	9,46E-06
Sellimonas	0	0	0	0
Woeseia	0	0,000008688	3,2242E-05	0
HT002	0	0	0	0
Ruegeria	0	0,000029322	0	0
Desulfofarcimen	0	0	0	0
MN 122.2a	0	0	0	0
Rhizorhapis	0	0	0	0
Roseibacterium	0	0	0	0
Duganella	0	0	0	0
Erysipelothrix	0	0	0	9,46E-06
Oscillospira	0	0	0	0
1174-901-12	0	0	0	0

Azorhizobium	0	0	0	0
TM7x	0	0	0	1,892E-05
Schleiferilactobacillus	0	0	0	0
Yokenella	0,000138592	0	0	2,838E-05
Thermovirga	0	0	0	0
Xanthomonas	0	0	0	3,784E-05
Ammoniphilus	0	0,000362724	0	0
Xiangella	0	0	0	0
Kineococcus	0	0	7,896E-06	0
Fonticella	0	0	0	0,0002743
DTU089	0	0	0	0
Punica	0	0,000147696	0	0
Epulopiscium	0	0	0	3,784E-05
Moryella	0	0	0	9,46E-06
Acidipila-Silvibacterium	0	0	0	0
Ancylobacter	0	0	0	0
Nodosilinea PCC-7104	0	0	0	0
Kabatiella	0	0	0	0
Neorhizobium	0	0	0	0
[Eubacterium] ruminantium group	0,000017324	0	0	0
CL500-29 marine group	0	0	0	0
Spirosoma	0	0	0	0
Scytonema PCC-7110	0	0	0	0
Pseudoxanthomonas	0	0,000004344	0	0
Planktotalea	0	0	0	0
Methylocella	0	0	0	0
Papillibacter	0	0	0	0
Alkaliphilus	0	0	0	0,0002554
V9D2013 group	0	0	0	0
Sedimenticola	0	0	0	3,784E-05
Anaerofilum	0	0	0	0
Moorella	0	0,000021177	3,2242E-05	0
Fibrobacter	0	0	0	9,46E-06
Caenimonas	0	0	0	0
Idiomarina	0,00004331	0	0	5,676E-05
Rheinheimera	0,000017324	0	0	9,46E-06
Pilibacter	0	0	0	5,676E-05
Rosenbergiella	0,00012993	0,000004344	0	2,838E-05
Terrisporobacter	0	0	0	7,568E-05
Perlucidibaca	0	0	0	0
Ercella	0	0	0	0,0002743
Teleaulax	0	0,000088509	3,2242E-05	0
Desemzia	0	0	0	9,46E-06
Victivallis	0	0	0	0
Chelatococcus	0	0	0	0
Paraglaciecola	0,000017324	0	0	1,892E-05
Nioella	0	0	0	0
Clostridium sensu stricto 3	0	0	0	0,000246
Liquorilactobacillus	0	0	0	0
Vishniacozyma	0	0	0	0
Sediminimonas	0	0	0	0
Ferruginibacter	0	0	0	0
Paraclostridium	0	0	0	0
Sporanaerobacter	0	0	0	0,0001514
Prunus	0	0,000029322	6,4484E-05	0

Faecalitalea	0	0	0	0
Thiothrix	0	0	0	3,784E-05
MB11C04 marine group	0	0	0	0
Parvibaculum	0	0,000008688	0	0
Cognatishimia	0	0	0	0
Pleiochaeta	0	0	0	0
Howardella	0	0	0	9,46E-06
Amphibacillus	0	0	0	0
Legionella	0	0	0	1,892E-05
Xenorhabdus	0,000199226	0	0	9,46E-06
Pigmentiphaga	0	0	0	0
Stakelama	0	0,000025521	0	0
Aggregatibacter	0,000112606	0	0	9,46E-06
Proteiniphilum	0	0	0	0
Paraprevotella	0	0,000016833	0	0
Hymenobacter	0	0	8,0276E-05	0
Candidatus Vestibaculum	0	0	0	0
Cibicides	0,000008662	0,000025521	4,0138E-05	0
Phoma	0	0	0	0
ZOR0006	0	0	0	0
NS5 marine group	0	0	2,4346E-05	0
Candidatus Glomeribacter	0	0	0	0
Flavonifractor	0	0	0	1,892E-05
Candidatus Flaviluna	0	0	0	0
Desulfobulbus	0	0,000008688	1,5792E-05	0
Clostridium sensu stricto 17	0	0	0	0,0001797
Saccharomonospora	0	0,000004344	0	0
Moritella	0,00004331	0	0	2,838E-05
Pisciglobus	0	0	0	1,892E-05
Clostridium sensu stricto 16	0	0	0	0,0002554
Thermomonas	0	0	0	1,892E-05
Succiniclasticum	0	0	0	0
Candida	0	0	0	0
Pleomorphomonas	0	0	0	0
Conchiformibius	0	0	7,896E-06	0
Avibacterium	0,000034648	0	0	1,892E-05
Formosa	0	0,000046155	0	0
Segetibacter	0	0	0	0
Anaerobacter	0	0	0	0,0002365
Stomatobaculum	0	0	0	0
Catenibacillus	0	0	0	9,46E-06
Vagococcus	0	0	0	1,892E-05
Paucibacter	0	0	0	0
Vogesella	0	0	0	0
Sphingoaurantiacus	0	0	0	0
Bdellovibrio	0	0	0	0
Acremonium	0	0	0	0
Pedosphaera	0	0	0	0
Nitrolancea	0	0	0	0
Paenochrobactrum	0	0	0	0
Agaricicola	0	0	0	0
Thiovirga	0	0	0	0
Clostridium sensu stricto 18	0	0	0	0,0001987
Frigoribacterium	0	0	0	0
Candidatus Actinomarina	0	0,000004344	7,896E-06	0

Gluconobacter	0	0	0	0
Intestinimonas	0	0	0	1,892E-05
Psychrobium	0	0	0	0
Dinoroseobacter	0	0	0	0
Coccomyxa	0	0	0	0
Bilophila	0,000008662	0	0	0
Ahrensia	0	0	0	0
Thiocystis	0	0	0	9,46E-06
Prevotellaceae UCG-004	0	0	0	9,46E-06
GKS98 freshwater group	0	0	0	9,46E-06
Pannonibacter	0	0	2,4346E-05	0
Chryseobacterium	0	0	1,5792E-05	1,892E-05
Tianweitania	0	0	0	0
Leptothrix	0	0	0	0
Alloiococcus	0	0	0	0
Robinsoniella	0	0	0	0
Serpentinicella	0	0	0	9,46E-06
Georgenia	0	0	0	0
Salinispora	0	0	0	0
Dermacoccus	0	0,000008688	0	0
Dysgonomonas	0	0	7,896E-06	0
Niorma	0	0,000016833	0	0
Tsukamurella	0	0	0	0
DS001	0	0	0	0
Fenollaria	0	0	0	0,0001608
Dielma	0	0	0	9,46E-06
Aeriscardovia	0	0,000004344	0	0
Eisenbergiella	0	0	0	0
Mycoplasma	0	0	4,0138E-05	9,46E-06
Wangella	0	0,000080364	0	0
Pelomonas	0	0	0	3,784E-05
Halothiobacillus	0	0	7,896E-06	0
Huakuichenia	0	0	0	0
Methylophaga	0	0	0	5,676E-05
Brenneria	0,000164578	0	0	0
Pseudohongiella	0	0	0	0,0001041
Monomastix	0	0	0	0
Pacificibacter	0	0	0	0
Marinibacterium	0	0	0	0
Anaerofustis	0	0	0	0,0001135
Plasmodium	0	0,000016833	4,0138E-05	0
Clostridium sensu stricto 2	0	0	0	0,0001324
Nelumbo	0	0	0	0
Gordonibacter	0	0	0	0
Mucispirillum	0	0	0	3,784E-05
Flavitalea	0	0	0	0
Arcticibacter	0	0	0	0
Halodurantibacterium	0	0	0	0
Allobranchiibius	0	0	0	0
Fluviicola	0	0	0	0
Azospirillum	0	0	0	0
Filomicrobium	0	0	0	0
Zea	0	0	0	0
Azovibrio	0	0	0	0
Sumerlaea	0	0	7,896E-06	0

Lacimonas	0	0	0	0
Tremiscus	0	0	0	0
Verticiella	0	0	0	0
Gyrodinium	0	0	0	0
Hungatella	0	0	0	0
Triastrum	0	0	0	0
Hirschia	0	0	0	0
Marinicaulis	0	0	0	0
Candidimonas	0	0	0	0
Roseococcus	0	0,000088509	0	0
Rhodoglobus	0	0	0	0
SUP05 cluster	0	0	0,00007238	0
HN-HF0106	0	0	0	0
Oceanicaulis	0	0	0	0
Catellicoccus	0	0	0	0
Friedmanniella	0	0	0	0
Pseudorhodobacter	0	0	0	0
Candidatus Parabeggiatoa	0	0	0	0
Saccharothrix	0	0	0	0
Alexandrium	0	0	0	0
Aquamicrobium	0	0	0	0
MM2	0	0,000004344	0	0
Slackia	0	0	0	1,892E-05
Fermentimonas	0	0	0	0
Lutimaribacter	0	0	0	0
Azohydromonas	0	0	0	0
[Eubacterium] nodatum group	0	0	0	2,838E-05
Ochromonas	0	0	0	0
Streptomonospora	0	0	7,896E-06	0
Herbinix	0	0	0	0
Candidatus Rosenkranzia	0,000121268	0	0	0
Tropicimonas	0	0	0	0
Povalibacter	0	0	0	0
Halolactibacillus	0	0	0	0
Litorimonas	0	0	0	0
Alkanindiges	0	0	7,896E-06	0
Cohaesibacter	0	0	0	0
Clavulicium	0	0	0	0
Corallococcus	0	0	0	0
Medicago	0	0,00003801	0	0
Halorhodospira	0	0,000004344	0	0
Ensete	0	0	0	0
Paeniclostridium	0	0	0	0
Dietzia	0	0	0	0
Ellin516	0	0	0	0
Amylibacter	0	0	0	0
AR15	0	0,00015204	0	0
Cellvibrio	0,000017324	0	0	3,784E-05
Stentor	0	0	0	0
Citricoccus	0	0	0	0
Roseobacter	0	0	0	0
Actimicrobium	0	0,000004344	0	0
Ilumatobacter	0	0	0	0
Terrimonas	0	0	0	0
Methylohalobius	0	0	0	0

Prevotellaceae YAB2003 group	0	0	0	0
Succinimonas	0	0	0	0
Actinospica	0	0	0	0
Syntrophococcus	0	0	0	0
Neocatenulostroma	0	0	0	0
Arsenicicoccus	0	0	0	0
P3OB-51	0	0	0	0
Taeseokella	0	0	0	0
Pseudahrensia	0	0	0	0
Polymorphobacter	0	0	0	0
Leptolyngbya PCC-6306	0	0	0	0
Johnsonella	0	0	0	0
Anaeroplasma	0,000008662	0	0	1,892E-05
Erythropsidinium	0	0	0	0
SD04E11	0	0	0	0
Xylophilus	0	0	0	0
Pragia	0,000060634	0	0	0
C1-B045	0,000008662	0	0	1,892E-05
Citreicella	0	0	0	0
Lentibacter	0	0	0	0
Rhodobaculum	0	0	0	0
Sedimentibacter	0	0	0	8,514E-05
Frateuria	0	0	0	0
Candidatus Hepatoplasma	0	0	0	0
Leptococcus JA-3-3Ab	0	0	0	0
Mycoplana	0	0	0	0
Paeniglutamicibacter	0	0	0	0
Sphaerotilus	0	0	0	0
Marivita	0	0	0	0
Sporobacter	0	0	0	0
FS140-16B-02 marine group	0	0	0	0
[Eubacterium] brachy group	0	0	0	7,568E-05
Sediminibacterium	0	0	0	0
Negativibacillus	0,000008662	0	0	2,838E-05
Blastomonas	0	0	0	0
SH-PL14	0	0	0	0
Tabrizicola	0	0	0	0
Pelagostrobilidium	0	0	0	0
UCG-004	0	0	0	0
[Clostridium] innocuum group	0	0	0	0
RS25G	0	0	0	0
Lagerheimia	0	0	0	0
Fructobacillus	0	0	0	0
Pseudofulvimonas	0	0	0	0
Leptolyngbya EcFYyyy-00	0	0	0	0
Clostridium sensu stricto 5	0	0	0	0,0001135
Sagittula	0	0	0	0
Anaerostignum	0	0	0	0
Hellea	0	0	4,0138E-05	0
Eikenella	0	0	0	0
Temperatibacter	0	0	0	0
UCG-012	0	0	0	0
Pelosinus	0	0	0	0
Luedemannella	0	0	0	0
Frisingicoccus	0	0	0	0

Jannaschia	0	0	0	0
Prototheca	0	0	0	0
Sharpea	0	0	0	0
Mogibacterium	0	0	0	3,784E-05
Ursidibacter	0	0,000004344	0	0
Salinarimonas	0	0	0	0
Ignavigranum	0	0	0	0
Triticum	0	0,000008688	0	0
Beijerinckia	0	0	0	0
Castellaniella	0	0	0	0
Stenotrophobacter	0	0	0	0
Phaselicystis	0	0	0	0
Agreia	0	0	0	0
Verrucosispora	0	0	0	0
[Rhizobium] sphaerophysae group	0	0	0	0
Dichotomicrobium	0	0,000004344	7,896E-06	0
Candidatus Vidania	0	0	0	0
Lentimicrobium	0	0	0	0
OM43 clade	0	0	0	0
Pseudoruegeria	0	0	0	0
Catenisphaera	0	0	0	0
Paludicola	0	0	0	0
Roseibacillus	0	0	0	0
Alkalimarinus	0	0	0	1,892E-05
Meiothermus	0	0	0	1,892E-05
Aphanizomenon NIES81	0	0	0	0
Tessaracoccus	0	0	0	0
Profundimonas	0	0	0	9,46E-06
CAG-56	0	0	1,5792E-05	0
Aquibacter	0	0	0	0
UBA6140	0	0	0	0
UCG-001	0	0	0	0
Pseudokineococcus	0	0	0	0
Thiogranum	0	0	0	0
Trigonobalanus	0	0	0	0
[Eubacterium] fissicatena group	0	0	0	0
Methylophilus	0	0,000004344	0	0
Dolosicoccus	0	0	0	9,46E-06
Marinimicrobium	0	0	0	0
Anaerosalibacter	0	0	0	1,892E-05
Martelella	0	0	0	0
Inhella	0	0	0	0
Parapusillimonas	0	0	0	0
Clostridium sensu stricto 9	0	0	0	0,0000473
Phreatobacter	0	0	0	0
Candidatus Chlorothrix	0	0	0	3,784E-05
Pseudohaliea	0	0	0	2,838E-05
Megamonas	0,000095282	0	0	0
Mariprofundus	0	0	0	0
Pseudohydnum	0	0	0	0
Cavicella	0	0	0	0,0000473
Bavariicoccus	0	0	0	0
Pseudovibrio	0	0	0	0
Ferritrophicum	0	0	0	0
Tepidimonas	0	0	0	0

[Eubacterium] cellulosolvens group	0	0	0	0
Amphiplicatus	0	0	0	0
Limibaculum	0	0	0	0
Starkeya	0	0	0	0
Kineosporia	0	0	0	0
Candidatus Nitrotoga	0	0	0	0
Candidatus Blochmannia	0	0	0	9,46E-06
Kribbella	0	0	0	0
Peptostreptococcus	0	0	0	9,46E-06
Anaerovorax	0	0	0	0
Caldicoprobacter	0	0	0	5,676E-05
Schwartzia	0	0	0	0
Anoxynatronum	0	0	0	0
Cardiobacterium	0,000025986	0	0	9,46E-06
Glenodinium	0	0	0	0
Stagonosporopsis	0	0	0	0
Aestuariibacter	0	0	0	0
Adhaeribacter	0	0	0	0
Longivirga	0	0	0	0
Dethiobacter	0	0	0	0
Blastocatella	0,000025986	0	0	0
XBB1006	0	0	0	0
Dyella	0	0	0	0
Eggerthella	0	0	0	0
Oxobacter	0	0	0	9,46E-06
Gymnosporangium	0	0	0	0
Yueomyces	0	0	0	0
Lachnospiraceae FE2018 group	0	0	0	0
SAR92 clade	0	0,000008688	0	9,46E-06
Puccinia	0	0	0	0
Niastella	0	0	0	0
UTBCD1	0	0	0	0
Ranunculus	0	0	0	0
Marinococcus	0	0,000016833	7,896E-06	0
Defluviitaleaceae UCG-011	0	0	0	0
Lachnospiraceae UCG-002	0	0	0	0
SEEP-SRB1	0	0	0	0
Piscibacillus	0	0	0	0
Jeotgalibacillus	0	0	0	0
Lachnospiraceae UCG-009	0	0	0	0
Simplicispira	0	0	0	0
Facklamia	0	0	0	0
Erysipelotrichaceae UCG-009	0	0	0	0
Caldilinea	0	0	0	0
Rhodomicrobium	0	0	0	0
Debaryomyces	0	0	0	0
Dellaglioa	0	0	0	0
Phalansterium	0	0	0	0
Mageeibacillus	0	0	0	0
Tateyamaria	0	0	0	0
Blvii28 wastewater-sludge group	0	0	0	0
Plantactinospora	0	0	0	0
Nesterenkonia	0	0	0	0
Glaciecola	0	0	0	9,46E-06
Blastopirellula	0	0,000025521	2,4346E-05	0

Solicoccozyma	0	0	0	0
Aeribacillus	0	0	0	0
Acidobacterium	0	0	0	0
Prauserella	0	0	0	0
Caloramator	0	0	0	1,892E-05
Desmodesmus	0	0	0	0
Crocinitomix	0	0	0	0
Kerstersia	0	0	0	0
Candidatus Nitrocosmicus	0	0	0	0
Sphaerulina	0	0	0	0
Erysipelotrichaceae UCG-006	0	0	0	9,46E-06
Peridinium	0	0	0	0
Methylobacillus	0	0	0	1,892E-05
Holdemania	0	0	0	0
Cloacibacterium	0	0	0	0
Phalacroma	0	0	0	0
Chitinophaga	0	0	0	0
Salirhabdus	0	0	0	0
Gonyaulax	0	0	0	0
Oleispira	0	0	0	9,46E-06
probable genus 10	0	0	0	0
Lentimonas	0	0	0	0
Ammonia	0	0,000004344	0	0
Salisediminibacterium	0	0	0	0
JL-ETNP-F27	0	0	0	0
HIMB11	0	0	0	0
Myxococcus	0	0	0	0
Rickettsiella	0	0	0	0
Labrys	0	0	0	0
BIyi10	0	0	0	1,892E-05
Hassallia	0	0	0	0
Arsenicitalea	0	0	0	0
Oikopleura	0	0	0,00005593	0
Thiohalorhabdus	0	0	0	0
[Agitococcus] lubricus group	0	0	0	0
Desulfobacca	0	0	0	0
Lachnospiraceae UCG-007	0	0	0	0
Thioalkalispira-Sulfurivermis	0	0	0	9,46E-06
Biostraticola	0,000060634	0	0	0
Egicoccus	0	0	0	0
Metschnikowia	0	0	0	0
DSSF69	0	0	0	0
Advenella	0	0	0	0
Cognatiyoonia	0	0	0	0
Clostridium sensu stricto 8	0	0	0	0
Angustibacter	0	0	0	0
Amaricoccus	0	0	0	0
Dolichospermum NIES41	0	0	0	0
Periconia	0	0	0	0
Neo-b11	0	0	0	0
Vermamoeba	0	0	0	0
Candidatus Xiphinematobacter	0	0	0	0
SN8	0	0	0	0
Pyronema	0	0	0	0
Malikia	0	0	0	0

W5053	0	0	0	0
Candidatus Tammella	0	0	0	9,46E-06
Kordiimonas	0	0	0	0
Erysipelotrichaceae UCG-007	0	0	0	0
Acidithiobacillus	0	0	0	9,46E-06
RBG-16-49-21	0	0	0	5,676E-05
Parvimonas	0	0	0	9,46E-06
Ureibacillus	0	0	0	0
Senegalimassilia	0	0	0	0
Helcococcus	0	0	0	0
Phytohabitans	0	0	0	0
Balneola	0	0	0	0
Oropetium	0	0	0	0
Aliihoeflea	0	0	0	0
Wendlandiella	0	0	0	0
Psychroglaciecola	0	0	0	0
Rodentibacter	0	0	0	0
Macellibacteroides	0	0	0	0
Pseudopropionibacterium	0	0	0	0
Methyloparacoccus	0,000008662	0	0	0
Acidocella	0,000034648	0	0	9,46E-06
Candidatus Paraholospora	0	0	0	0
Denitrobacterium	0	0	0	0
Qipengyuania	0	0	0	0
Oceanimonas	0,00004331	0	0	9,46E-06
Kluyveromyces	0	0	0	0
Coleochaete	0	0	0	0
Neochlorosarcina	0	0	0	0
1959-1	0	0	0	9,46E-06
Aurantisolimonas	0	0	0	0
Desulfobacter	0	0,000004344	0	0
Candidatus Omnitrophus	0	0	0	0
Taphrina	0	0	0	0
Nostoc PCC-73102	0	0	0	0
Clostridium sensu stricto 7	0	0	0	2,838E-05
Salipaludibacillus	0	0	0	0
Lapidilactobacillus	0	0	0	0
Sva0996 marine group	0	0	0	0
GOUTA6	0	0	0	0
Ampullimonas	0	0	0	0
DEV008	0	0	0	0
Acetatifactor	0	0	0	9,46E-06
Holospora	0	0	0	0
Hansschlegelia	0	0	0	0
Myrothecium	0	0	0	0
Hespellia	0	0	0	0
Marinifilum	0	0	0	0
Keratinibaculum	0	0	0	0
Enorma	0	0	0	0
Caldimonas	0	0	0	0
Limimaricola	0	0	0	0
Candidatus Kinetoplastibacterium	0	0	0	0
Pseudoclostridium	0	0	0	0
Thalassiosira	0	0	0	0
Thlaspi	0	0,000029322	0	0

Anurofeca	0	0	0	0
Rimostrombidium	0	0	0	0
Caldicellulosiruptor	0	0	0	0
Aquisphaera	0	0	0	0
P3OB-42	0	0	0	0
Clostridiisalibacter	0	0	0	9,46E-06
Atopobium	0	0	0	0
Methylosinus	0	0	0	0
Krasilnikovia	0	0	0	0
Terracidiphilus	0	0	0	0
Candidatus Puniceispirillum	0	0	0	0
Microcyclospora	0	0	0	0
Erysipelotrichaceae UCG-002	0	0	0	0
Methylocapsa	0	0	0	0
Sulfuricella	0	0	0	0
Draconibacterium	0	0	0	0
Sinophysis	0	0	0	0
Lachnoanaerobaculum	0	0	0	0
Anaerobiospirillum	0,000034648	0	0	0
Mannheimia	0	0	0	0
Thermolongibacillus	0	0,000016833	0	0
CHKCI001	0	0	0	0
Halioglobus	0	0	0	0
Jhaorihella	0	0	0	0
Tumebacillus	0	0	0	0
Anaeroglobus	0	0	0	0
Lachnobacterium	0	0	0	0
JTB215	0	0	0	0
Chloronema	0	0	0	0
Tetratrichomonas	0	0	0	0
Ethanoligenens	0	0	0	0
Pseudotontonia	0	0	0	0
Colpodella	0	0	0	0
Geminigera	0	0	0	0
Chitinibacter	0	0	0	0
Halioxenophilus	0	0	0	9,46E-06
Taylorella	0	0	0	0
Limibacillus	0	0	0	0
Chaetomium	0	0	0	0
Niveispirillum	0	0	0	0
Stigmatodiscus	0	0	0	0
Phillipsia	0	0	0	0
Strombidium	0	0	0	0
Chitinimonas	0	0,000004344	0	0
Octadecabacter	0	0	0	0
Phaeosclera	0	0	0	0
Amnibacterium	0	0	0	0
Hungateiclostridium	0	0	0	0
Protoperidinium	0	0	0	0
Kistimonas	0	0	0	0
Pasteurella	0	0	0	0
Dapisostemonum CCIBt 3536	0	0	0	0
Auricoccus-Abyssicoccus	0	0	0	0
Paludibaculum	0	0	0	0
Subtercola	0	0	0	0

Pseudoglutamicibacter	0	0	0	0
Sporocytophaga	0	0	0	0
Tolumonas	0	0	0	0
Verminephrobacter	0	0	0	0
Desulfosporosinus	0	0	0	0
Segniliparus	0	0	0	0
Cetobacterium	0	0	0	2,838E-05
Alicyclobacillus	0	0	0	0
Lacimicrobium	0	0	0	9,46E-06
Paucisalibacillus	0	0	0	0
Candidatus Tenderia	0	0	0	0
Beggiatoa	0	0	0	0
Dactylosporangium	0	0	0	0
MIZ17	0	0	0	0
Anaerovibrio	0	0	0	0
Rhodovulum	0	0	0	0
Lonepinella	0	0	0	0
Tetragenococcus	0	0	0	0
Roseinatronobacter	0	0	0	0
ADurb.Bin118	0	0	0	0
Luminiphilus	0	0	0	0
Lacibacter	0	0	0	0
Syntrophaceticus	0	0	0	0
Niabella	0	0	0	0
Sandaracinus	0	0	0	0
Winogradskyella	0	0	0	0
Sediminispirochaeta	0	0	0	0
Spirochaeta	0	0	0	0
Pinus	0	0	0	0
Marinicella	0	0	0	0
CM1G08	0	0,000004344	7,896E-06	0
Lechevalieria	0	0	0	0
F0332	0	0	0	0
Hydrogenispora	0	0	0	0
Teratosphaeria	0	0	0	0
Neodesmus	0	0	0	0
Bulbochaete	0	0	0	0
Rhogostoma	0	0	0	0
Nodularia PCC-9350	0	0	0	0
Leucocryptos	0	0	0	0
Hydrogenoanaerobacterium	0	0	0	9,46E-06
Lutibacter	0	0	0	0
Tissierella	0	0	0	1,892E-05
Rhodomonas	0	0	0	0
Rikenella	0	0	7,896E-06	0
ASF356	0	0	0	0
Epistominella	0	0	1,5792E-05	0
Thorea	0	0,000004344	0	0
Pseudorhodoferax	0	0	0	0
Amycolatopsis	0	0	0	0
Ectothiorhodospira	0	0	0	9,46E-06
Coleofasciculus PCC-7420	0	0	0	0
Cellulosilyticum	0	0	0	0
DEV114	0	0	0	0
Chaetoceros	0	0	0	0

Ureaplasma	0	0	0	0
Gracilibacter	0	0	0	0
Agarivorans	0	0	0	0
Methanobacterium	0	0	0	0
Aureicoccus	0	0	0	0
Rudaea	0	0	0	0
Chloracidobacterium	0	0	0	0
Codosiga	0	0	0	0
Mayaca	0	0	0	0
Apostasia	0	0	0	0
Hibiscus	0	0	0	0
Pyropia	0	0	0	0
Chilodochona	0	0	0	0
Verticillium	0	0	0	0
Atlantibacter	0	0	0	0
Agarilytica	0	0	0	0
Xylomelasma	0	0	0	0
Alicycliphilus	0	0	0	0
Motilimonas	0	0	0	0
Donghicola	0	0	0	0
Patulibacter	0	0	0	0
Elizabethkingia	0	0	0	0
Parasegetibacter	0	0	0	0
Synechococcus MBIC10613	0	0	0	0
Exutisphaerella	0	0	0	0
Antarctobacter	0	0	0	0
Candidatus Curculioniphilus	0	0	0	0
Chromobacterium	0	0	0	9,46E-06
Candidatus Hamiltonella	0	0	0	9,46E-06
Desulfoplanes	0	0	0	9,46E-06
Angelakisella	0	0	0	0
Vitreoscilla	0	0	0	0
Thiorhodococcus	0	0	0	0
Taibaiella	0	0	0	0
Dinghuibacter	0	0	0	0
Puniceicoccus	0	0	0	0
SEEP-SRB4	0	0	0	0
Desulfomonile	0	0	0	0
Halanaerobium	0	0	0	0
Halothermothrix	0	0	0	0
wb1-A12	0	0	0	0
A07HB70	0	0	0	0
Salinigranum	0	0	0	0
Halonotius	0	0	0	0
Ochrolechia	0	0	0	0
Viridiraptor	0	0	0	0
Metopion	0	0	0	0
Strobilidium	0	0	0	0
Barranca	0	0	0	0
Crustomastix	0	0	0	0
Eubacterium	0	0	0	1,892E-05
Oblitimonas	0	0	0	0
Ahniella	0	0	0	0
Candidatus Bacilloplasma	0	0	0	1,892E-05
Asaia	0	0	0	0

LCP-80	0	0	0	0
Phytomonospora	0	0	0	0
Polaribacter	0	0	0	0
Diplosphaera	0	0	0	0
Caldalkalibacillus	0	0	0	0
Thermoclostridium	0	0	0	9,46E-06
Blastodinium	0	0	0	0
Haloactinopolyspora	0	0	0	0
Pseudochelatococcus	0	0	0	0
Acidicapsa	0	0	0	0
Ascochyta	0	0	0	0
FCPS473	0	0	0	1,892E-05
Blastochloris	0	0	0	0
Candidatus Desulforudis	0,000017324	0	0	0
Citrus	0	0	0	0
Abditibacterium	0	0	0	0
Jeotgalicoccus	0	0	0	0
Gallaecimonas	0	0	0	9,46E-06
Bodomorpha	0	0	0	0
Papiliocellulus	0	0	0	0
Aliterella	0	0	0	0
Urania-1B-19 marine sediment group	0	0	0	0
Roseobacter clade Marinomonas lineage	0	0	0	0
Desulforhopalus	0	0	0	0
HTCC5015	0	0	0	0
Ornithinimicrobium	0	0	0	0
Ferrimonas	0	0	0	9,46E-06
Oligotropha	0	0	0	0
Silicimonas	0	0	0	0
Phyllosticta	0	0,000025521	0	0
Candidatus Ancillula	0	0	0	0
Fontimonas	0	0	0	0
Aequorivita	0	0	0	0
Microbulbifer	0	0	0	0
Candidatus Competibacter	0	0	0	0
Acetivibrio	0	0	0	0
Amoebophrya	0	0	0	0
Aliivibrio	0	0	0	0
Paludibacter	0	0	0	0
Allofustis	0	0	0	0
Amoebogregarina	0	0	0	0
Libanicoccus	0	0	0	0
Thalassobaculum	0	0	0	0
Candidatus Baumannia	0	0	0	0
Caedibacter	0	0	0	0
Setosphaeria	0	0	0	0
Vigna	0	0	0	0
Cosmarium	0	0	0	0
Radiococcus	0	0	0	0
Cercomonas	0	0	0	0
Phytophthora	0	0	0	0
Maricaulis	0	0	0	0
Bombella	0	0	0	0
Shimazuella	0	0	0	0
AAP99	0	0	0	0

Smittium	0	0	0	0
Paramaledivibacter	0	0	0	0
KCM-B-112	0	0	0	0
Chryseolinea	0	0	0	0
Guggenheimella	0	0	0	9,46E-06
Pedococcus-Phycicoccus	0	0	0	0
Desulfofundulus	0	0	0	0
Calothrix PMC232.04	0	0	0	0
Plectosphaerella	0	0	0	0
Coprobacillus	0	0	0	0
Quinella	0	0	0	0
Succinivibrionaceae UCG-002	0	0	0	0
Candidatus Planktophila	0	0	0	0
Chlorobium	0	0	0	0
Psychroflexus	0	0	7,896E-06	0
Peptococcus	0	0	0	0
Cryptosporidium	0	0	0	0
Parvularcula	0	0	0	0
Desulfonispora	0	0	0	0
Floricoccus	0	0	0	0
Jiangella	0	0	0	0
Schleiferia	0	0	0	0
Neopseudocercosporella	0	0	0	0
Paraphaeosphaeria	0	0	0	0
Chaetosphaeridium	0	0,000021177	0	0
Sporacetigenium	0	0	0	0
Tepidibacter	0	0	0	0
Anaeromicrobium	0	0	0	0
Salinarchaeum	0	0	0	0
Exophiala	0	0	0	0
Aquimonas	0	0	0	0
Hydrolithon	0	0	7,896E-06	0
[Renibacterium] salmoninarum group	0	0	0	0
Enterovibrio	0	0	0	0
Geomicrobium	0	0	0	0
Marine Methylotrophic Group 3	0	0	0	0
Cryptococcus	0	0	0	0
Haloimpatiens	0	0	0	9,46E-06
Acetobacterium	0	0	0	9,46E-06
Thermoflexus	0	0	0	0
Helianthus	0	0	0	0
Phanerochaete	0	0	0	0
Thalassospira	0	0	0	0
Budvicia	0	0	0	0
Eionea	0	0	0	0
Prochlorothrix PCC-9006	0	0	0	0
Thismia	0	0	0	0
Theileria	0	0	0	0
Chrysochromulina	0	0	0	0
OLI16029	0	0	0	0
Simiduia	0	0	0	0
Nitrincola	0	0	0	0
Trabulsiella	0	0	0	9,46E-06
Sulfurospirillum	0	0	0	9,46E-06
Haloplasma	0	0	0	0

Proteocatella	0	0	0	9,46E-06
Clostridium sensu stricto 6	0	0	0	1,892E-05
BSV13	0	0	0	0
Pithites	0	0	0	0
Dongia	0	0	0	0
Marinomonas	0	0	0	9,46E-06
Aquicella	0	0	0	0
Magnetococcus	0	0	0	0
Litorimicrobium	0	0	0	0
Lachnotalea	0	0	0	0
[Bacteroides] pectinophilus group	0	0	0	0
Terriglobus	0	0	0	0
Candidatus Koribacter	0	0	0	0
Chlamydia	0	0	0	0
Desulfatibacillum	0	0	0	0
Nostoc PCC-7524	0	0	0	0
Asteroma	0	0	0	0
Bicosta	0	0	0	0
Macrococcus	0	0	0	0
Ereboglobus	0	0	0	0
Cephaloticoccus	0	0	0	0
Dubosiella	0	0	0	0
Grimontia	0,000008662	0	0	0
Thioalkalivibrio	0	0	0	0
Gallibacterium	0	0	0	0
Kouleothrix	0	0	0	0
Thiohalophilus	0	0	0	0
Chthonobacter	0	0	0	0
Alloscardovia	0	0	0	0
Actinoallomurus	0	0	0	0
Fischerella PCC-9339	0	0	0	0
Cohnella	0	0	7,896E-06	0
Agaribacterium	0	0	0	0
Litoricola	0	0	0	0
Desulfobacterium	0	0	0	9,46E-06
Candidatus Portiera	0	0	0	0
Soehngenia	0	0	0	9,46E-06
Lampropedia	0	0	0	0
Hydrogenophilus	0	0	0	0
Thermopetrobacter	0	0	0	0
Nostoc PCC-7107	0	0	0	0
Guyparkeria	0	0	0	0
Paucimonas	0	0	0	0
Glycomyces	0	0	0	0
Tatumella	0,000017324	0	0	0
Flavimaricola	0	0	0	0
Phocoenobacter	0	0	0	0
Pectinodesmus	0	0	0	0
Bibersteinia	0	0	0	0
Methylovirgula	0	0	0	0
Paenisporosarcina	0	0	0	0
Salegentibacter	0	0	0	0
Formivibrio	0	0	0	0
Rhodopila	0	0	0	0
Rhodoligotrophos	0	0	0	0

Gloeophyllum	0	0	0	0
Pseudoflavonifractor	0	0	0	0
Candidatus Ovatusbacter	0	0	0	0
Methylobacter	0	0	0	0
Dehalobacterium	0	0	0	0
Desulfonema	0	0	0	0
Picea	0	0	0	0
Hyphomonas	0	0	0	9,46E-06
Anderseniella	0	0	0	0
Longispora	0	0	0	0
Tolypothrix	0	0	0	0
Cloacibacillus	0	0	0	0
Herminiimonas	0	0	0	0
Aestuariimicrobium	0	0	0	0
Antricoccus	0	0	0	0
Saccharopolyspora	0	0	0	0
Pseudomuriella	0	0	0	0
Thalassotalea	0,000008662	0	0	0
Laceyella	0	0	0	0
Neomicrococcus	0	0	0	0
Enteractinococcus	0	0	0	0
Renibacterium	0	0	0	0
Nakamurella	0	0	0	0
U29-B03	0	0	0	0
Dactylococcopsis PCC-8305	0	0	1,5792E-05	0
Daucus	0	0	1,5792E-05	0
Paralactobacillus	0	0	0	0
Magnetovibrio	0	0	0	0
Nevskia	0	0	0	0
OM75 clade	0	0	0	0
Crenobacter	0	0	0	0
Brachyconidiellopsis	0	0	0	0
Roseimarinus	0	0	0	0
Prevotellaceae Ga6A1 group	0	0	0	0
[Aquaspirillum] arcticum group	0	0	0	0
Siccibacter	0	0	0	0
Thorsellia	0	0	0	0
Chryseomicrobium	0	0	0	0
Geodermatophilus	0	0	0	0
Nonomuraea	0	0	0	0
Candidatus Uzinura	0	0	0	0
Alkalinema CENA528	0	0	0	0
Desulfatiglans	0	0	0	0
Candidatus Megaira	0	0	7,896E-06	0
Tanticharoenia	0	0	0	0
Anaerosphaera	0	0	0	0
Thermobrachium	0	0	0	0
Thermotunica	0	0	0	0
Acidipropionibacterium	0	0	0	0
Gloeocapsa	0	0	0	0
AKYG587	0	0	0	0
Pseudactinotalea	0	0	0	0
Macromonas	0	0	0	0
Ketobacter	0	0	0	0
Bryocella	0	0	0	0

Pedinomonas	0	0	0	0
Biecheleria	0	0	0	0
Sarcocystis	0	0	0	0
Heteromita	0	0	0	0
Leptolegnia	0	0	0	0
Trachydiscus	0	0	0	0
Rhodovastum	0	0	0	0
Christensenella	0	0	0	0
Savagea	0	0	0	0
Faecalicoccus	0	0	0	0
Petrimonas	0	0	0	0
Phyllonema CENA325	0	0	0	0
Annamia HOs24	0	0	0	0
Pir2 lineage	0	0	0	0
Candidatus Methanoplasma	0	0	0	0
RumEn M2	0	0	0	0
Candidatus Riegeria	0	0	0	0
Paraphysomonas	0	0	0	0
Marinoscillum	0	0	0	0
Sterolibacterium	0	0	0	0
Albidovulum	0	0	0	0
Swaminathania	0	0	0	0
Filifactor	0	0	0	0
Williamsia	0	0	0	0
Actinopolymorpha	0	0	0	0
Microbispora	0	0	0	0
Richelia HH01	0	0,000004344	0	0
Francisella	0	0	0	0
Amphisorus	0	0	0	0
Flavicella	0	0	0	0
Amphora	0	0	0	0
NS10 marine group	0	0,000004344	7,896E-06	0
Persicirhabdus	0	0	0	0
Prosthecomicrobium	0	0	0	0
Aplanochytrium	0	0	0	0
Salinicola	0	0	0	0
Candidatus Carsonella	0	0	0	0
Sneathiella	0	0	0	0
Propionibacterium	0	0	0	0
Catalinimonas	0	0	0	0
Limnothrix	0	0	0	0
Helvella	0	0	0	0
Olpidium	0	0	0	0
Coelomomyces	0	0	0	0
Mougeotia	0	0	0	0
Chiayiivirga	0	0	0	0
Holophaga	0	0	0	0
Viridibacillus	0	0	0	0
Desulfatitalea	0	0	0	0
Bowmanella	0	0	0	0
Lonsdalea	0	0	0	0
Carnimonas	0	0	0	0
Aestuariicella	0	0	0	0
Candidatus Berkiella	0	0	0	0
Halofilum	0	0	0	0

Candidatus Armantifilum	0	0	0	0
Echinicola	0	0	0	0
Parapedobacter	0	0	0	0
Methanohalobium	0	0	0	0
Dichelobacter	0	0	0	0
Hydrogenobacter	0	0	0	0
Thiocapsa	0	0	0	0
Candidatus Thiosymbion	0	0	0	0
Thioalbus	0	0	0	0
Candidatus Captivus	0	0	0	0
DNF00809	0	0	0	0
Chujaibacter	0	0	0	9,46E-06
Succinatimonas	0	0	0	9,46E-06
Orbus	0	0	0	9,46E-06
Saccharospirillum	0	0	0	9,46E-06
2013Ark19i	0	0	0	9,46E-06
Luteibacter	0	0	0	9,46E-06
Inquilinus	0	0	0	9,46E-06
[Anaerorhabdus] furcosa group	0	0	0	9,46E-06
Sebaldella	0	0	0	9,46E-06
Geobacter	0	0	0	9,46E-06
Endomicrobium	0	0	0	9,46E-06
Desulfuromusa	0	0	0	0
Caldibacillus	0	0	0	0
Candidatus Ruthia	0	0	0	0
Sphaerochaeta	0	0	0	0
Candidatus Purcelliella	0	0	0	0
Propionimicrobium	0	0	0	0
Herbihabitans	0	0	0	0
Pseudobacteroides	0	0	0	0
Olleya	0	0	0	0
Waddlia	0	0	0	0
Amphidiniella	0	0	0	0
Quercus	0	0	0	0
Nonlabens	0	0,000004344	0	0
Aliikangiella	0	0	0	0
Mitsuaria	0	0	0	0
Tepidiphilus	0	0	0	0
Chitinivorax	0	0	0	0
Oleibacter	0	0	0	0
Methylomarinovum	0	0	0	0
IheB2-23	0	0	0	0
Candidatus Hodgkinia	0	0	0	0
Haematobacter	0	0	0	0
Eremococcus	0	0	0	0
EMP-G18	0	0	0	0
Actinopolyspora	0	0	0	0
OLB12	0	0	0	0
Algoriphagus	0	0	0	0
Raineya	0	0	0	0
Parachlamydia	0	0	0	0
SCGC AAA164-E04	0	0	0	0
Singulisphaera	0	0	0	0
Chloroflexus	0	0	0	0
Synechocystis BDHKU-20401	0	0	0	0

Aminivibrio	0	0	0	0
Parengyodontium	0	0	0	0
Pycnococcus	0	0	0	0
Aquipuribacter	0	0,000008688	0	0
Alishewanella	0,000008662	0	0	0
Thermus	0,000008662	0	0	0
Chromohalobacter	0	0	0	0
Tepidamorphus	0	0	0	0
Lachnospiraceae UCG-003	0	0	0	0
Acholeplasma	0	0	0	0
Veillonellaceae UCG-001	0	0	0	0
Mailhella	0	0	0	0
Haloplanus	0	0	0	0
Pasteuria	0	0	0	0
HSB OF53-F07	0	0	0	0
Asteroleplasma	0	0	0	0
Histophilus	0	0	0	0
Thiopseudomonas	0	0	0	0
Oceanobacter	0	0	0	0
Vulcaniibacterium	0	0	0	0
Cocleimonas	0	0	0	0
Candidatus Jidaibacter	0	0	0	0
Eilatimonas	0	0	0	0
Granulicoccus	0	0	0	0
Coriobacterium	0	0	0	0
Catonella	0	0	0	0
possible genus Sk003-Sk004	0	0	0	0
Rummeliibacillus	0	0	0	0
Entomoplasma	0	0	0	0
TC1	0	0	0	0
Sulfobacillus	0	0	0	0
BacC-u-018	0	0	0	0
Coprobacter	0	0	0	0
Salinimicrobium	0	0	0	0
MAT-CR-P4-C12	0	0	0	0
Sphaerospermopsis BCCUSP55	0	0	0	0
Synechocystis PCC-6803	0	0	0	0
Phormidesmis ANT.LACV5.1	0	0	0	0
Phormidium MBIC10003	0	0	0	0
Sneathia	0	0	0	0
Propionigenium	0	0	0	0
Vulcanisaeta	0	0	0	0
Taonella	0	0	0	0
Cellulosibacter	0	0	0	0
Karlodinium	0	0	0	0
Myrionecta	0	0	0	0
Arcocellulus	0	0	0	0
Micromonas	0	0	0	0
Sulfurirhabdus	0	0	0	0
Murdochiella	0	0	0	0
Thiophaeococcus	0	0	0	0
Pleurocapsa PCC-7319	0	0	0	0
Methanimicrococcus	0	0	0	0
Euglena	0	0	7,896E-06	0
Natronolimnobius	0	0	7,896E-06	0

Candidatus Nitrosopumilus	0	0	0	0
Litoribacillus	0	0	0	0
Haloferula	0	0	0	0
Nitrosarchaeum	0	0	0	0
Methyloprofundus	0	0	0	0
Pontibacter	0	0	0	0
Raoultibacter	0	0	0	0
Hylemonella	0	0	0	0
Paralcaligenes	0	0	0	0
Paenalcaligenes	0	0	0	0
Terasakiispira	0	0	0	0
Candidatus Ecksteinia	0	0	0	0
Xylella	0	0	0	0
Thioalkalicoccus	0	0	0	0
Inmirania	0	0	0	0
Candidatus Liberibacter	0	0	0	0
Sphingomicrobium	0	0	0	0
Roseobacter clade CHAB-I-5 lineage	0	0	0	0
Paenirhodobacter	0	0	0	0
Anaplasma	0	0	0	0
Candidatus Xenohaliotis	0	0	0	0
Gluconacetobacter	0	0	0	0
Candidatus Paracaedibacter	0	0	0	0
Candidatus Arthromitus	0	0	0	0
Pontibacillus	0	0	0	0
Baia	0	0	0	0
Succinispira	0	0	0	0
Ornithinicoccus	0	0	0	0
Propioniciclava	0	0	0	0
Streptosporangium	0	0	0	0
Alkaliflexus	0	0	0	0
Subsaxibacter	0	0	0	0
NS2b marine group	0	0	0	0
Myroides	0	0	0	0
Hydrotalea	0	0	0	0
Parafilimonas	0	0	0	0
Cnuella	0	0	0	0
Leptolyngbya ANT.L52.2	0	0	0	0
Litorilinea	0	0	0	0
Phycisphaera	0	0	0	0
Polyangium	0	0	0	0
Sorangium	0	0	0	0
Halarsenatibacter	0	0	0	0
Halobacteroides	0	0	0	0
Funneliformis	0	0	0	0
Telotrochidium	0	0	0	0
Procabacter	0	0	0	0
Maribacter	0	0	0	0
Robertkochia	0	0	0	0
Owenweeksia	0	0	0	0
Flavimarina	0	0	0	0
Amphidiniopsis	0	0	0	0
Phascolodon	0	0	0	0
Haloarcula	0	0	0	0
R76-B128	0	0	0	0

Diplorickettsia	0	0	0	0
Allocatelliglobosispora	0	0	0	0
Hoppeia	0	0	0	0
Croceibacter	0	0	0	0
NS3a marine group	0	0	0	0
Candidatus Sulcia	0	0	0	0
Coraliomargarita	0	0	0	0
Balechina	0	0	0	0
Euglenaria	0	0	0	0
Phaeodactylibacter	0	0	0	0
Carboxylicivirga	0	0	0	0
Halobacteriovorax	0	0	0	0
Cristigera	0	0	0	0
pLW-20	0	0,000004344	0	0
Methylovulum	0	0,000004344	0	0
Defluviitalea	0	0,000004344	0	0
Limibacter	0	0,000004344	0	0
Aureispira	0	0,000004344	0	0
Planktothrix NIVA-CYA 15	0	0,000004344	0	0
Psychrilyobacter	0	0,000004344	0	0
NOR26	0	0,000004344	0	0
Maritimimonas	0	0	0	0
Dichanthelium	0	0	0	0
Amphidinium	0	0	0	0

Sample35	sample20	sample24	sample32	sample38	sample40	sample55
0,00044352	0,00769055	0,002035968	0,003899126	0,293958123	0,00234222	0,0100869
0,00044928	0,000801248	0,000135168	0,000094042	0,108003351	0,000207692	0,0005056
0,000597888	0,001334221	0,00071808	0,00933186	0,000086967	0,001794944	0,0010455
0,022278528	0,049645183	0,00912384	0,029464082	0,000260901	0,019148596	0,1312753
0,021174912	0,01319913	0,00494208	0,02448709	0,000057978	0,00403256	0,1037141
0,000114048	0	0	0	0	0	8,57E-06
0,000062208	0,007822899	0,00177408	0,006525068	0,000222249	0,00050786	0,0021425
0,000296064	0,000443548	0,185019648	0,000925952	0,000028989	0,000039416	0,0000857
0,000232704	0,002711366	0,00071808	0,001215312	0,170493972	0,000883828	0,002391
0,000398592	0,001155371	0,000557568	0,009035266	0,000009663	0,00050786	0,0041822
0,000131328	0,000890673	0,000439296	0,001150206	0,122604144	0,000442672	0,0009427
0,000050688	0,000490049	0,000202752	0,000383402	0,007653096	0,000207692	0,000977
0,000415872	0,001334221	0,0004224	0,008152718	0,007276239	0,000820156	0,0040879
0,00141696	0,005068609	0,001554432	0,017455642	0,002096871	0,00297894	0,011141
0,00002304	0,000042924	0,169771008	0,000629358	0	0,000051544	0
0,004853376	0,002357243	0,001579776	0,005360394	0,000019326	0,000650364	0,0252387
0,00004032	0,000132349	0,033597696	0,000752336	0,000009663	0,001314372	2,571E-05
0,000073728	0,000089425	0,112341504	0,00043404	0,000019326	0,000051544	0,0001114
0,003146112	0,006846378	0,001681152	0,006517834	0,000019326	0,002666644	0,0253586
0,00001728	0,000622398	0,16862208	0,00014468	0,000048315	0,000104604	0,0001286
0	0,004088511	0,000177408	0,00560635	0,000028989	0,000130376	0,0002143
0,00003456	0,000089425	0,00016896	0,000875314	0,000009663	0,000025772	0,0001286
0,00049536	0,024091095	0,004131072	0,015473526	0,000357531	0,005554624	0,0121351
0,000415872	0,009954791	0,005626368	0,022215614	0,000367194	0,002367992	0,0251272
0	0,000132349	0,000135168	0,00090425	0,000048315	0,000585176	0,0002228
0,002156544	0,006710452	0,001123584	0,009751432	0,000028989	0,001976864	0,0261899
0,00004032	0,00017885	0,000321024	0,00177233	0,034120053	0,000116732	0,0003771
0,00004608	0,000089425	0,000329472	0,010026324	0,017741268	0,000077316	0,0003857
0,002827008	0,000844172	0,000608256	0,002285944	0	0,000559404	0,0080644
0	0,00017885	0,000118272	0,000188084	0,031829922	0,000065188	0,0001543
0	0,000042924	0,000016896	0	0,000038652	0	0
0,002150784	0,000490049	0,000059136	0,00079574	0	0,000039416	0,0005571
0,000056448	0,001598919	0,000261888	0,003595298	0,000057978	0,00054576	0,0062218
0	0,000443548	0,000016896	0,008991862	0,000125619	0,001794944	0,0043793
0	0,017377066	0,004705536	0,005237416	0,000686073	0,00403256	0,0106439
0	0	0,000025344	0,000021702	0,010368399	0	1,714E-05
0,000205056	0,004310285	0,000430848	0,004709334	0,000048315	0,000976304	0,0024767
0,000631296	0,003065489	0,000371712	0,003240832	0,000028989	0,001079392	0,0066075
0,00002304	0,000042924	0,000092928	0,000245956	0,016639686	0	6,856E-05
0,00050112	0,000311199	0,000143616	0,001128504	0,014446185	0,000377484	0,0002057
0,00044928	0,000844172	0,000413952	0,0010851	0	0,000624592	0,0081758
0,0000288	0,002131892	0,00029568	0,009917814	0,000106293	0,001365916	0,0020225
0,00088704	0	0,000109824	0,000094042	0	0,000025772	0,0005485
0	0,000042924	0,00608256	0,00039787	0,000009663	0	0,0001971
0,001598976	0	0	0	0	0,003589888	0
0,000228096	0,000443548	0,00004224	0,001721692	0,000028989	0,000247108	0,001654
0	0,000089425	0,000016896	0,000050638	0	0,000051544	0,0375195
0,000699264	0,001423646	0,000895488	0,00423189	0,000028989	0,00078074	0,003548
0	0,000311199	0,000050688	0,000014468	0,017924865	0,000013644	8,57E-06
0,00096192	0,000042924	0,012350976	0,00003617	0,000009663	0,000025772	1,714E-05
0,000978048	0,000976521	0,000447744	0,001244248	0	0,0004548	0,0061104
0,00000576	0	0,000033792	0,003139556	0,000028989	0,000013644	1,714E-05
0,00050688	0,002443091	0,000642048	0,00332764	0,000009663	0,000976304	0,0098041
0,000557568	0,003777312	0,000996864	0,002285944	0,000028989	0,00141746	0,0112867

0	0,000132349	0,00004224	0,00025319	0,000009663	0,000104604	3,428E-05
0	0,000089425	0,000016896	0,000014468	0,013006398	0,000013644	7,713E-05
0	0,000890673	0,000574464	0,002126796	0,000106293	0,000221336	0,0059647
0,000307584	0,005290383	0,000481536	0,00184467	0,000077304	0,000832284	0,0065561
0,000648576	0,001198295	0,00038016	0,003696574	0	0,000494216	0,006796
0	0,010934889	0,000177408	0	0,00077304	0,00150842	0,0067874
0,0018432	0,000221774	0,00008448	0,000224254	0,000009663	0,0004548	0,0002485
0,000602496	0,00164542	0,000523776	0,00184467	0,000009663	0,000559404	0,0076787
0,000056448	0,000132349	0,0002112	0,000267658	0	0,00054576	0,0069674
0,00001728	0,002178393	0,00025344	0,001888074	0,000009663	0,000286524	0,0000857
0,001165824	0,001023022	0,000388608	0,004651462	0,000028989	0,000559404	0,0016626
0	0,000089425	0	0,000028936	0,008261865	0,000013644	5,999E-05
0,000551808	0,00146657	0,001638912	0,00495529	0,00009663	0,00192532	0,0047478
0,000102528	0,000089425	0,000067584	0,010619512	0	0,000039416	0,0021682
0,000528768	0,000622398	0,000447744	0,008521652	0,000038652	0,000403256	0,0051934
0,000119808	0	0,000076032	0,000101276	0,004280709	0,00009096	0,0001286
0	0,003512614	0,000143616	0,002669346	0,000028989	0,000664008	0,0011912
0	0	0,000016896	0,00007234	0,018379026	0,000013644	0
0,00000576	0,000532973	0,001039104	0,014019492	0,00028989	0,002589328	0,0006085
0,000756864	0,000354123	0,0002112	0,000781272	0,000009663	0,000247108	0,0021511
0,000733824	0,000268275	0,000185856	0,00097659	0,000019326	0,000988432	0,0058019
0	0,000490049	0,002593536	0,01027228	0,000028989	0,000051544	0,002751
0	0,000443548	0,000050688	0,000159148	0,006213309	0,000065188	2,571E-05
0,0000288	0,002579017	0,00025344	0,003638702	0,000019326	0,000676136	0,0019797
0,000091008	0,000443548	0,000016896	0,000622124	0	0,000130376	0,0010284
0,00000576	0,000221774	0,012241152	0,000057872	0,000009663	0,000013644	0
0	0	0,000076032	0,000014468	0,010609974	0	5,999E-05
0	0,000042924	0,000016896	0,001258716	0,000009663	0,000013644	8,57E-06
0,00001152	0,003287263	0,000523776	0,002488496	0,000048315	0,001274956	0,0015769
0,000369792	0,000844172	0,00008448	0,000745102	0	0,000142504	0,002631
0,00001152	0	0	0,000007234	0	0	8,57E-06
0	0	0	0	0	0	0
0,00001152	0,002890216	0,000304128	0,002741686	0,000009663	0,001105164	0,0013969
0	0	0	0,003682106	0	0,000142504	0,0005656
0,00047232	0,000042924	0	0,000665528	0	0,000013644	0,0002742
0,00044928	0,002088968	0,000692736	0,001598714	0,000009663	0,001170352	0,0055791
0	0,005200958	0,001233408	0,004130614	0,000463824	0,001431104	0,0030595
0	0,000042924	0,000016896	0,00003617	0	0	8,57E-06
0	0,000221774	0,000033792	0,005505074	0	0,000377484	0,0002314
0,00048384	0,000089425	0,000194304	0,000332764	0,000009663	0,000156148	5,999E-05
0,000228096	0,000490049	0,000261888	0,00647443	0,000028989	0,000195564	0,001774
0	0	0,01364352	0,000050638	0	0	0
0,000346752	0	0	0,000065106	0	0,000013644	5,999E-05
0	0,000132349	0,000016896	0,000260424	0	0	7,713E-05
0,00001152	0	0	0,000412338	0,000009663	0,000013644	3,428E-05
0,000699264	0	0	0,000303828	0	0	0,0022625
0,0000288	0,001065946	0,00135168	0,004680398	0,000154608	0,001027848	0,001594
0	0,000042924	0,000016896	0,000014468	0,00241575	0,000025772	3,428E-05
0,00000576	0,000354123	0,000109824	0,006452728	0,000106293	0,002692416	0,0000857
0,000056448	0,003555538	0,000489984	0,003985934	0,000009663	0,00063672	0,0005742
0,000733824	0	0,000033792	0,000065106	0	0,000403256	0
0,001177344	0,000532973	0,000109824	0,000137446	0,000009663	0,000573048	1,714E-05
0	0,000132349	0	0,00198935	0	0	0,0004114
0	0	0	0	0	0	0
0	0,004979184	0,001148928	0,000115744	0,000048315	0,001158224	0,0031109

0,00001728	0,000042924	0	0,000050638	0	0,000104604	0,0001028
0	0	0	0,007733146	0	0	0
0	0	0	0,00137446	0	0,000039416	5,999E-05
0,00047808	0,00017885	0,000177408	0,000853612	0,000009663	0,00009096	0,0013712
0,000193536	0,001423646	0,00016896	0,001743394	0,000048315	0,001482648	0,0026481
0,001757952	0	0	0,00047021	0	0,000039416	0,0010027
0	0,000042924	0,000059136	0,000094042	0,004193742	0,000013644	3,428E-05
0,000091008	0,000443548	0,000557568	0,001960414	0,000048315	0,000610948	0,0009856
0	0,000622398	0,000101376	0,000491912	0,000086967	0,000104604	0,0002314
0	0,00017885	0,000067584	0,002799558	0,000048315	0,0004548	0,0058876
0	0	0	0	0	0	0
0	0	0	0,002828494	0	0,000013644	0,001714
0,000364032	0,000221774	0,000059136	0,00122978	0,000009663	0,000077316	0,001037
0,000358272	0,001423646	0,000236544	0,000499146	0	0,000468444	0,0032995
0	0	0	0,000028936	0	0	0,0003257
0,00000576	0,000089425	0,011979264	0,00007234	0	0,000025772	1,714E-05
0	0	0,000025344	0	0,000009663	0,000013644	8,57E-06
0,000170496	0,000490049	0,000177408	0,00047021	0	0,000221336	0,0023568
0	0,000042924	0,000008448	0	0,000086967	0,000013644	8,57E-06
0,000164736	0,001244796	0,000177408	0,000962122	0	0,000233464	0,0027253
0	0	0	0	0	0	0
0	0,000490049	0,000135168	0,002394454	0,000028989	0,000624592	0,0001028
0	0	0	0	0	0	0
0,00001152	0	0	0,00007234	0	0,000051544	0,0002228
0,000125568	0,000089425	0,000025344	0,001750628	0,000038652	0,001196124	0,0016969
0	0	0,000016896	0,01132121	0	0,000013644	0
0	0	0	0	0	0	0
0	0,000089425	0,000025344	0,000028936	0,000009663	0,000013644	1,714E-05
0,00000576	0,000221774	0,000033792	0,000101276	0,000028989	0,00027288	7,713E-05
0	0	0	0	0	0	0
0	0	0	0	0	0	0
0	0,00017885	0,000025344	0,004514016	0,000048315	0,000156148	0,0001543
0	0,000089425	0,000025344	0,000636592	0,000019326	0,000169792	0,0001971
0,000381312	0,00017885	0,000059136	0,000238722	0	0,000051544	0,0017226
0,000096768	0	0,000008448	0,00137446	0	0	0
0,000079488	0,000268275	0,000050688	0,00343615	0,000009663	0,00018192	0,0008227
0	0,005555081	0,000287232	0,002148498	0,000202923	0,001626668	9,427E-05
0	0	0	0,002054456	0	0,000195564	1,714E-05
0,000346752	0,000754747	0,000059136	0,000549784	0,000009663	0,000338068	0,0019454
0	0,000532973	0	0,00014468	0	0,000039416	0,0002228
0,000523008	0	0,00046464	0,00423189	0,000009663	0,000013644	0,001157
0	0,000754747	0,000016896	0,003899126	0,000009663	0,000104604	0,0049192
0	0	0,000025344	0,000021702	0,003565647	0,000013644	0,0001371
0,0000288	0,000711823	0,000912384	0,000419572	0,000009663	0,000025772	0,00012
0,00001152	0,000042924	0,00008448	0,00936803	0,000048315	0,000039416	0,0001971
0,000085248	0,000443548	0,000135168	0,003081684	0,00019326	0,000156148	0,0016797
0	0	0	0,010351854	0	0	0
0	0,00604513	0,000059136	0,000571486	0,000009663	0,000025772	0,000677
0,00004032	0,000622398	0,000101376	0,00021702	0	0,000195564	0,001157
0,00004032	0,000221774	0,000067584	0,000094042	0	0,000065188	0,0009513
0,000193536	0,000443548	0,0004224	0,000390636	0	0,000104604	0,0021511
0	0	0,000008448	0,000701698	0,000009663	0,000025772	8,57E-06
0	0	0	0,000774038	0,000009663	0,000025772	0,0001971
0,000228096	0,000221774	0,00012672	0,000557018	0	0,000039416	0,0019968
0,0000288	0,000221774	0,005482752	0,001403396	0,000009663	0,000533632	0,0003599

0,000073728	0,000089425	0,000092928	0,000752336	0,000860007	0,000104604	0,0006256
0,000096768	0,000801248	0,000194304	0,00043404	0,000019326	0,00027288	0,001774
0,00000576	0,000622398	0	0,00010851	0	0	7,713E-05
0	0	0,00008448	0	0,009170187	0	0
0,000114048	0,000579474	0,000092928	0,000564252	0	0,000442672	0,0010027
0	0,000132349	0,000008448	0,000448508	0	0,000832284	8,57E-06
0,00000576	0,000132349	0,000025344	0,001403396	0,000019326	0,000142504	8,57E-06
0	0	0,005769984	0,000014468	0	0,000013644	8,57E-06
0,000096768	0,000844172	0,000101376	0,000448508	0	0,00018192	0,0011912
0,00001152	0,000268275	0,000016896	0,001041696	0	0,000247108	0,0004371
0,000255744	0	0,000016896	0,000694464	0	0,000142504	0,0001371
0,000141696	0,000132349	0,00012672	0,002842962	0	0,000104604	0,0012255
0	0,002310742	0,000101376	0,000057872	0	0,000298652	0,003548
0,000108288	0,00017885	0,000025344	0,000412338	0	0,000247108	0,000857
0,000096768	0,000042924	0,000016896	0,000151914	0	0,000013644	0,0007456
0,000141696	0,000354123	0,000135168	0,00054255	0,000028989	0,000389612	0,0018768
0	0	0	0,00086808	0,000009663	0,000207692	0
0,00001728	0,000042924	0,000008448	0,00148297	0	0,000013644	0,0037794
0	0	0,000008448	0,006684216	0	0	0
0	0	0	0	0	0	0,002751
0,00000576	0	0	0	0,000019326	0	0
0,000147456	0,000089425	0,000101376	0,000231488	0,000009663	0,000142504	0,0015512
0	0,000443548	0	0	0	0,000442672	1,714E-05
0	0,000490049	0,000143616	0,001208078	0,000028989	0,000767096	0,0014312
0,0000288	0,000844172	0,000244992	0,000426806	0,000009663	0,000298652	0,0028795
0	0	0,000008448	0,007132724	0	0	8,57E-06
0,000067968	0,000221774	0,000067584	0,000419572	0,000028989	0,000142504	0,000797
0,000085248	0,000579474	0,00008448	0,000672762	0	0,000142504	0,0012255
0	0	0	0	0	0	0,0031281
0,00000576	0,000221774	0	0,00184467	0	0	0,0001028
0	0	0	0,000303828	0	0	0
0,000296064	0,000042924	0,000016896	0,003248066	0,000009663	0,000130376	0,0000857
0	0	0,00004224	0,000267658	0,000019326	0,000065188	0,0002228
0	0	0	0,000007234	0	0	0
0	0	0,000008448	0,000007234	0	0,000585176	0,0064618
0	0,000221774	0,000059136	0,000311062	0,000019326	0,000207692	0,00018
0	0	0	0,006865066	0	0	0
0	0	0	0	0	0	0
0,001570176	0	0	0,00014468	0,000009663	0,000077316	0,0004371
0	0,000089425	0,00004224	0,00003617	0	0	0,0008913
0	0,000042924	0,000109824	0,000788506	0	0	0,0004971
0,00003456	0,000579474	0,0002112	0,000376168	0	0,000051544	0,001774
0,000073728	0,000089425	0,000033792	0,000151914	0	0	0,0003599
0,000131328	0,000268275	0,000016896	0,000115744	0	0,000013644	0,0008399
0,00001728	0	0,000016896	0,005924646	0,000009663	0	0
0,000056448	0,000268275	0,000135168	0,000593188	0,000009663	0,000065188	0,0016883
0,000563328	0	0,000185856	0,000086808	0	0	0,0002314
0	0	0,000016896	0,000028936	0,001372146	0	0
0,0000288	0	0,000008448	0,000021702	0	0	0,0001028
0,000067968	0,000400624	0,000152064	0,001439566	0,000019326	0,0004169	0,0005999
0,000091008	0,000221774	0,000033792	0,00018085	0	0,000130376	0,0006685
0	0,001112447	0	0,000173616	0,000338205	0,000013644	0,0027167
0	0	0	0	0	0	0
0	0	0	0,004579122	0,000086967	0	8,57E-06
0	0	0	0,00025319	0	0,00009096	0,000677

0,000523008	0,000354123	0,000008448	0,000159148	0	0,000233464	0,0002485
0	0,000622398	0,000076032	0,000860846	0,000125619	0,000338068	0,0005056
0,000056448	0,000042924	0,000016896	0,000065106	0	0,000025772	9,427E-05
0	0	0	0,000007234	0	0	8,57E-06
0	0	0,000312576	0,000028936	0,000347868	0	8,57E-06
0	0	0,002889216	0,000079574	0	0	0
0,000091008	0	0,00004224	0,000137446	0	0	0,0005142
0,000131328	0,000311199	0,000109824	0,000745102	0	0,000221336	0,0016026
0,000125568	0,000443548	0,00004224	0,000260424	0	0,000104604	0,0006513
0	0	0	0	0	0,000039416	0
0	0,000221774	0,00004224	0,00032553	0	0,000077316	0,0001714
0	0,005333307	0,000008448	0,000014468	0,000019326	0,000025772	8,57E-06
0,00000576	0,000042924	0,00367488	0,000028936	0	0,000013644	8,57E-06
0	0	0	0,000810208	0	0	0
0	0,000042924	0	0,003501256	0,000009663	0,000013644	0,00012
0	0	0	0	0	0	0
0,00001152	0	0	0,002741686	0	0	2,571E-05
0	0	0	0	0	0	0
0	0	0	0	0	0	0
0	0	0	0	0	0,000013644	0
0	0,000042924	0	0,001497438	0,000009663	0	0
0	0,000221774	0,000059136	0,00021702	0	0,000065188	0,0007199
0,00002304	0,00017885	0,000025344	0,00010851	0,000009663	0,000013644	0,0002571
0,0000288	0,000890673	0,000076032	0,00047021	0	0,000585176	0,0006256
0,000067968	0,000400624	0,000067584	0,000224254	0	0,00009096	0,000977
0	0	0	0,000007234	0	0	8,57E-06
0,00004032	0	0,000008448	0,00094042	0	0,000039416	0,0003685
0	0	0,000008448	0,000007234	0,001033941	0,000013644	2,571E-05
0,00000576	0	0	0	0	0	0
0,000114048	0,000400624	0,000016896	0,00086808	0	0,000221336	0,0007627
0	0	0	0,00007234	0	0	1,714E-05
0	0	0,000016896	0,000065106	0,000753714	0	0
0	0	0	0	0	0	0
0,000147456	0	0,000135168	0,000245956	0	0,000195564	0,0006342
0	0	0,0012672	0,000007234	0	0	0
0,00004032	0,000042924	0,000016896	0,000159148	0,001043604	0,000013644	0,0005056
0,000199296	0,000042924	0,000016896	0,00003617	0,000019326	0,000233464	8,57E-06
0,0000288	0	0,000185856	0,00021702	0,000154608	0,000025772	0,0002828
0,000284544	0,000443548	0,000067584	0,000050638	0	0,000533632	0
0	0	0	0	0	0	0
0	0,000042924	0,000016896	0,000057872	0	0	0
0,00000576	0	0	0,000007234	0	0	0,0001028
0,00003456	0,000089425	0	0,000173616	0,001043604	0,000065188	5,999E-05
0	0,000268275	0,000033792	0,001511906	0,000048315	0,000610948	0,0000857
0	0	0	0	0	0	0
0	0,000311199	0,000008448	0,000368934	0	0,000013644	0,0002057
0,0000288	0,000221774	0,000059136	0,00014468	0	0,00009096	0,0013198
0	0	0	0	0	0	1,714E-05
0	0,00017885	0,000059136	0,000260424	0	0,000039416	0,0003342
0	0	0	0,00245956	0	0	8,57E-06
0	0	0	0	0	0	0
0	0,00017885	0,000050688	0,00010851	0,000028989	0,000351712	0,0001628
0	0	0	0,00119361	0	0	0
0,00000576	0,000089425	0,00038016	0,000057872	0,000009663	0,000025772	0,0002143
0	0	0,000016896	0,000057872	0	0	8,57E-06

0,000131328	0,000132349	0,000059136	0,000209786	0	0,000013644	0,0005399
0,000335232	0,000311199	0,000016896	0,000296594	0	0,000051544	0,0007113
0	0	0,000050688	0,001909776	0	0	0
0	0	0,000329472	0,00021702	0	0	4,285E-05
0,000062208	0	0,000033792	0,000094042	0	0	0,0005742
0,00000576	0,000042924	0,000008448	0,00350849	0	0,000025772	0
0	0	0	0,003899126	0	0	0
0	0,000132349	0	0,000708932	0	0,000104604	0,0007456
0	0	0	0,000007234	0,000009663	0,00018192	8,57E-06
0,000056448	0,000443548	0,00004224	0,000173616	0,000019326	0,000065188	0,0005485
0,00004608	0	0,000059136	0,00007234	0	0,000065188	0,0008313
0	0,000042924	0,000016896	0,00043404	0,000019326	0,000013644	0,0005313
0	0	0	0,000050638	0	0	0
0	0	0	0	0	0	0
0	0,000042924	0,0006336	0,0018085	0,00009663	0,000077316	7,713E-05
0,00000576	0,000042924	0,000016896	0,000267658	0	0,000065188	0,0002571
0	0	0,000025344	0,002126796	0	0,000624592	1,714E-05
0	0,00017885	0,000109824	0,000086808	0,000067641	0,00059882	0,0000857
0,00002304	0	0	0,003284236	0	0	5,999E-05
0	0,000754747	0,000658944	0,000115744	0	0	1,714E-05
0,00004032	0,000400624	0,00008448	0,000130212	0	0,000142504	0,0009341
0,00004608	0	0,000059136	0,000817442	0,000028989	0,000013644	0,0004971
0	0	0	0,00021702	0	0	0,0001543
0	0	0	0,000050638	0	0,000025772	7,713E-05
0	0,002711366	0,000236544	0,000050638	0,000048315	0,000039416	7,713E-05
0	0	0	0	0	0	0,0033252
0	0,000443548	0,000025344	0,000477444	0,000009663	0,000207692	0,0001714
0	0	0,000008448	0,00245956	0	0	0
0,00001152	0,000311199	0,000059136	0,000188084	0	0,000233464	0,0004628
0	0,000132349	0,000025344	0,000238722	0	0,000664008	0,0001371
0	0	0	0	0	0	0
0,00001728	0,000042924	0,000033792	0,00007234	0	0	5,142E-05
0	0	0,000008448	0,000007234	0	0	0,0020568
0,00001728	0	0	0,000173616	0	0,000025772	5,999E-05
0,00002304	0,000089425	0,000101376	0	0	0	0,0004028
0,000153216	0	0	0,000115744	0	0,000013644	0,0002057
0	0,00017885	0	0,000028936	0	0,00009096	0,0005399
0	0	0	0,001982116	0	0	0
0,000091008	0,000268275	0,00004224	0,00010851	0	0,000077316	0,000797
0,00001152	0,000579474	0	0,002213604	0	0	0
0,00004032	0,00017885	0,000101376	0,000557018	0,000009663	0,000025772	0,0003685
0	0	0	0,001815734	0	0	0
0	0	0	0	0	0	0
0	0	0	0	0	0	0
0	0	0	0,002792324	0	0,000013644	0
0	0	0	0,000021702	0	0,000077316	1,714E-05
0	0	0,000025344	0,002806792	0	0	0
0,00001152	0,000311199	0,00008448	0,000079574	0	0,00018192	0,0004456
0	0,00128772	0	0,000209786	0	0,000221336	0,0005828
0,00000576	0	0,000008448	0,000021702	0	0,000051544	8,57E-06
0,000067968	0,000221774	0,000016896	0,000028936	0	0,000195564	0,0004371
0	0,000042924	0	0,000115744	0	0	0
0	0	0	0,000007234	0	0	0
0	0	0	0,000014468	0	0	0
0	0,000042924	0,000008448	0	0	0	2,571E-05

0	0	0	0,002416156	0	0	8,57E-06
0	0	0	0	0	0,000025772	0
0	0	0,002525952	0,000007234	0	0	0
0	0	0,00173184	0,000014468	0	0	0
0	0	0,001157376	0	0	0	0
0,000073728	0,000132349	0,00004224	0,000188084	0	0,000039416	0,0003342
0	0	0,000016896	0,000007234	0	0,000025772	8,57E-06
0	0,000665322	0,00016896	0,000173616	0,000106293	0,000494216	0,0001286
0,00001152	0,000754747	0,000033792	0,000094042	0	0,000130376	0,0005399
0	0	0	0,00047021	0	0,000013644	0
0	0	0	0	0	0	0,0004628
0	0	0	0,002488496	0	0	8,57E-06
0	0,000042924	0,000033792	0,000115744	0	0,000039416	0,0005828
0,00003456	0	0,000067584	0,000079574	0	0,000013644	0,0002828
0	0,001777769	0	0	0	0	0
0,0000288	0	0,000016896	0,000079574	0	0,000025772	0,0004628
0	0,000042924	0,000016896	0,00003617	0,000502476	0	1,714E-05
0	0	0	0,000007234	0	0,000729196	8,57E-06
0	0	0	0	0	0	0,0001286
0	0	0	0,000130212	0	0	0
0,00002304	0	0	0,0018085	0	0,000013644	0,0001286
0	0	0	0,000014468	0	0	0
0	0,000042924	0,000016896	0,000014468	0	0,000013644	7,713E-05
0	0	0	0	0	0	0
0	0	0	0,000159148	0	0	8,57E-06
0,000244224	0,000042924	0,000008448	0,000014468	0,000009663	0,000130376	0
0	0	0	0	0	0,000025772	0,0003171
0,0000288	0	0	0	0,000647421	0	0
0	0	0	0,00007234	0	0,000039416	2,571E-05
0	0	0	0,000173616	0	0,000065188	0,0001543
0,000056448	0,000089425	0,00025344	0,000209786	0	0,000077316	0,0003342
0,00001152	0,000221774	0,000059136	0,00043404	0,000009663	0,000039416	0,0003171
0	0	0	0,00039787	0	0,000013644	5,142E-05
0	0,000622398	0,00004224	0,000014468	0	0	0,0005228
0,000096768	0	0	0,000014468	0	0,000013644	0,00018
0	0	0	0	0	0	0
0	0	0	0,00007234	0	0,000077316	0,0001286
0	0,000042924	0,000008448	0	0,000028989	0	0
0,00000576	0,000042924	0	0,000166382	0	0,000013644	1,714E-05
0	0,000042924	0,00004224	0,00021702	0,000135282	0	0,0002742
0	0	0	0	0	0	8,57E-06
0	0,000042924	0,000008448	0,00003617	0	0	3,428E-05
0	0	0	0,000007234	0	0,000013644	7,713E-05
0	0	0	0,000137446	0	0,000025772	0,0003599
0	0,000042924	0	0,000166382	0,000019326	0	0,0002314
0	0	0,000059136	0,00007234	0	0	8,57E-06
0	0	0	0	0,000251238	0	0
0,00004608	0,000089425	0,000008448	0,000094042	0,000009663	0,000039416	2,571E-05
0	0	0	0	0	0	0
0	0	0	0,00162765	0	0	0
0	0,000089425	0	0,002054456	0	0	0
0	0	0	0,002126796	0	0	0
0,00000576	0,000579474	0,000033792	0,000115744	0	0,000051544	0,0004885
0,00000576	0	0,000016896	0,001801266	0,000009663	0	9,427E-05
0	0,000042924	0	0,000079574	0	0	1,714E-05

0	0,001598919	0,000025344	0,00003617	0,000048315	0,000039416	0
0	0	0	0,002119562	0	0	0
0	0	0	0,000130212	0	0,000013644	0
0	0	0	0,00202552	0	0	3,428E-05
0	0	0	0,000007234	0,000898659	0	1,714E-05
0	0	0	0,000057872	0	0	8,57E-06
0	0	0	0	0	0	0
0	0	0	0,000014468	0,000009663	0	8,57E-06
0,00001728	0,000132349	0,000008448	0,000065106	0	0	0,0004371
0	0	0	0,000477444	0	0,000013644	0,0015255
0	0,000400624	0,000025344	0,00028936	0	0,000156148	0,0004714
0	0	0	0,000021702	0	0	4,285E-05
0,000085248	0	0,000016896	0	0	0	0,0001028
0,00000576	0	0,000016896	0	0,000376857	0	0
0	0,000354123	0,000025344	0,000115744	0,000009663	0,000130376	0,0001457
0	0	0	0	0,000946974	0	0
0,00002304	0,000042924	0	0,000875314	0	0,000025772	0,0001714
0	0	0	0,001388928	0	0	8,57E-06
0,00001152	0,000132349	0	0	0,000154608	0	8,57E-06
0,00004608	0,000089425	0,000008448	0,000209786	0	0,000051544	0,0001971
0	0,000042924	0	0,001504672	0	0	0,0000857
0,00001152	0,00017885	0,000008448	0,000050638	0	0,000039416	0,0001114
0,000141696	0,000042924	0,000008448	0,000962122	0,000009663	0	3,428E-05
0,00043776	0,000042924	0	0	0	0,000051544	0
0	0	0,000059136	0,000079574	0,000009663	0,000156148	0,0008999
0	0	0	0	0,000009663	0	0
0	0	0	0	0	0	0
0	0	0	0	0	0,000013644	0
0	0	0	0	0	0	0
0	0,000089425	0,000016896	0,000007234	0,000144945	0,00123554	0
0,0000288	0	0,000016896	0,000007234	0,000299553	0	4,285E-05
0,000147456	0,000089425	0,000008448	0,00003617	0,000009663	0,000051544	8,57E-06
0	0,000354123	0,000177408	0,00007234	0,000212586	0	8,57E-06
0,00000576	0	0,000008448	0,000021702	0	0,000013644	0,0002228
0	0	0	0	0	0,000025772	0
0,00003456	0,000042924	0,000016896	0,000137446	0	0,000025772	0,0003257
0	0	0	0,001685522	0	0	0
0	0,000354123	0	0,000137446	0,000009663	0,000077316	0,0001628
0	0	0	0	0,000415509	0	0
0	0	0,000008448	0,000028936	0	0,000025772	8,57E-06
0	0	0	0	0	0	0,0001885
0	0,000042924	0,000008448	0,000245956	0	0,000039416	0,0003
0	0	0	0	0	0	1,714E-05
0	0	0	0,001642118	0	0,000013644	0
0	0,000089425	0,000016896	0,00003617	0	0,000025772	0,0006085
0	0	0	0,000716166	0	0	0,0002057
0	0,00017885	0,000016896	0,000376168	0	0,000104604	1,714E-05
0	0,00017885	0	0,00112127	0,000009663	0,000025772	1,714E-05
0	0,000042924	0,000244992	0,000166382	0,000009663	0,00054576	4,285E-05
0,000125568	0	0,000016896	0	0	0	0
0	0	0	0,000007234	0	0	0
0,00002304	0	0	0,001128504	0,000048315	0	0
0	0	0	0,000014468	0,000299553	0,000013644	1,714E-05
0	0	0	0,001005526	0	0	0,0005313
0	0,000089425	0	0,000101276	0	0,000025772	0,0001028

0	0,00146657	0	0,000007234	0	0	0
0	0	0	0	0	0	0
0	0	0	0	0	0	0
0	0	0,000092928	0,000933186	0,000019326	0,000039416	0
0,00002304	0,000042924	0,000008448	0,00007234	0	0	0,0003
0,00003456	0	0,000008448	0,000086808	0	0,000039416	0,0001371
0	0	0	0	0	0	0
0	0	0	0,001461268	0	0	0
0	0	0	0,000007234	0,000396183	0	8,57E-06
0	0	0	0	0	0	8,57E-06
0	0	0	0	0	0	0
0	0	0	0,0014468	0	0	0
0	0	0	0	0	0,000701908	0
0,00001152	0	0	0,000101276	0	0,000013644	0
0	0	0	0,000028936	0	0	1,714E-05
0	0,000089425	0,000025344	0,000014468	0	0,000013644	0,0004456
0,00002304	0	0,000008448	0,00003617	0	0,000065188	0,0001543
0,00001728	0	0	0,000028936	0	0	0,0001457
0	0	0	0,001381694	0,000009663	0	0
0	0	0	0,000028936	0	0	5,142E-05
0	0,000042924	0	0,00068723	0	0	8,57E-06
0,00000576	0,000042924	0,000016896	0,00018085	0	0,000051544	0,0003
0	0	0	0,000347232	0	0,000156148	4,285E-05
0,00001152	0	0,000016896	0,00003617	0	0,000025772	0,0001543
0	0	0	0,001345524	0	0	0
0	0,000042924	0,000008448	0,00025319	0,000009663	0,000051544	1,714E-05
0	0	0	0	0	0	0
0	0	0	0	0	0	0
0	0	0,000008448	0,000086808	0	0,000051544	3,428E-05
0	0,000042924	0,000836352	0,000014468	0,000009663	0,000025772	0
0,0000288	0,000042924	0,000008448	0,000021702	0	0,00009096	0,0002057
0	0,000042924	0	0,000007234	0	0,000013644	1,714E-05
0	0	0,000008448	0	0	0,000077316	3,428E-05
0	0	0	0	0	0,000025772	0
0,000238464	0,000089425	0	0	0	0,000039416	0
0	0	0	0,000021702	0	0	0
0	0	0	0	0	0	0
0,000085248	0	0	0,000065106	0	0	0,0001371
0	0,000042924	0	0	0,000309216	0	0
0,00001152	0	0,000008448	0,000101276	0,000009663	0,000025772	0,0002657
0,00000576	0	0	0	0	0	0
0,00003456	0,000490049	0,000008448	0,000115744	0	0,000013644	4,285E-05
0	0,000132349	0,000008448	0,000007234	0	0,000013644	2,571E-05
0	0	0	0,000021702	0	0	0
0	0,000042924	0,000008448	0,00007234	0	0,000039416	0,0002057
0	0	0	0	0,00028989	0	8,57E-06
0,000079488	0,000132349	0,00004224	0	0	0	0,0001028
0	0	0	0,00119361	0	0	0
0	0	0	0,000086808	0	0	0
0,00001152	0,000089425	0,000008448	0,000260424	0	0,000039416	2,571E-05
0	0	0	0	0	0	0
0,000193536	0	0,000008448	0,000658294	0,000009663	0	0
0	0	0	0,00122978	0	0	0
0	0	0	0	0	0	0
0	0	0	0	0,000212586	0	0

0,00000576	0	0,000008448	0,000549784	0	0	8,57E-06
0	0	0,000008448	0,001200844	0	0	0
0,00003456	0	0,000008448	0,000021702	0	0,000039416	0,0001371
0	0	0	0,000028936	0	0	0
0	0	0	0	0	0	0
0	0	0	0	0	0	8,57E-06
0,00001152	0,000268275	0	0,000101276	0	0,000104604	4,285E-05
0	0,000042924	0,000016896	0,000130212	0	0,000025772	0,0003685
0	0	0	0	0,000270564	0	1,714E-05
0	0	0	0,000065106	0	0,000013644	0,0001114
0	0	0,000050688	0	0	0	0,0004456
0	0	0,000270336	0,000007234	0	0,000013644	0,0008399
0	0,000221774	0	0	0	0	0
0,0000288	0,000042924	0,000050688	0,000065106	0	0,00009096	0,0002914
0	0	0	0	0	0	0
0	0	0	0,000766804	0	0	0
0,00000576	0	0,0002112	0,000585954	0	0	8,57E-06
0	0	0	0	0,000212586	0	0
0	0,000042924	0	0,000007234	0	0,00068978	0
0	0	0	0,000086808	0	0,000013644	0,0002228
0,000119808	0	0	0	0	0,000077316	0
0	0	0	0,00104893	0	0	8,57E-06
0	0	0	0,000101276	0	0	0
0	0	0	0	0	0	0
0	0	0,000008448	0	0	0	0,0001371
0	0	0	0,000094042	0	0	0
0	0	0	0,000101276	0	0,000025772	0,0003771
0	0,000042924	0,000033792	0,000086808	0	0,000077316	0,0003
0	0	0	0	0	0	0
0	0	0	0	0	0	0
0	0	0	0,000810208	0	0	0
0	0,000042924	0	0,000021702	0	0,00009096	3,428E-05
0	0	0	0,000159148	0,000009663	0,000116732	7,713E-05
0	0	0	0,000716166	0	0	0
0,0000288	0	0	0,000028936	0	0	5,999E-05
0	0	0	0,000513614	0	0	0,0004114
0	0	0	0	0,000028989	0	0
0	0	0	0,000007234	0	0,000013644	0,0009427
0	0,000089425	0	0,000007234	0,000009663	0,000013644	0,0001028
0	0	0,000016896	0,000007234	0,000222249	0	0
0,0000288	0,000042924	0,000016896	0,000057872	0,000009663	0,000025772	8,57E-06
0	0,000042924	0,000008448	0,000050638	0,000106293	0,000013644	0,0000857
0,00000576	0	0	0,00039787	0	0,000039416	5,999E-05
0	0	0	0	0	0,000988432	0
0	0	0	0	0	0	0
0,00001152	0,000042924	0,000016896	0,000057872	0	0,000013644	0,0001971
0	0	0	0,000925952	0	0	0
0,00002304	0,000132349	0,000008448	0,000079574	0	0,00009096	9,427E-05
0	0	0	0,000094042	0,000009663	0	0,0008484
0	0,000354123	0	0	0	0,000051544	8,57E-06
0,00001152	0,000311199	0	0,000079574	0	0,000025772	0,0001371
0	0	0	0,000050638	0,000338205	0	0
0,00002304	0	0	0	0	0	0
0	0	0	0,00032553	0	0,000039416	8,57E-06
0	0	0	0,00003617	0	0,000025772	4,285E-05

0	0,000622398	0,00008448	0,000151914	0	0,000013644	8,57E-06
0	0	0	0	0	0	0
0,00001728	0	0,000016896	0	0,000009663	0,000013644	0,0003171
0	0	0	0	0,000077304	0	0
0,00003456	0,000042924	0,000016896	0,000057872	0,000009663	0,000039416	0
0	0	0,000008448	0,000014468	0	0,000013644	7,713E-05
0,00001728	0	0,000016896	0	0,000231912	0	1,714E-05
0	0,000622398	0,00008448	0	0,000009663	0,000013644	0
0	0	0,000008448	0,000014468	0,000009663	0	0,0005742
0	0	0	0	0	0	0
0	0	0	0,000585954	0	0,000298652	0
0,000102528	0,000132349	0	0	0	0,000065188	8,57E-06
0,000193536	0	0,000025344	0,000014468	0,000009663	0,000494216	1,714E-05
0	0	0,000118272	0,000658294	0,000009663	0	0
0	0,000132349	0,000016896	0,000079574	0	0,000025772	0,0002228
0	0	0,000447744	0,000086808	0,000009663	0,000051544	3,428E-05
0	0	0	0,000007234	0	0,000741324	8,57E-06
0	0	0	0,000050638	0	0	0
0	0	0	0,000007234	0	0,000013644	0,0001028
0	0	0,000008448	0	0	0,000025772	8,57E-06
0,0000288	0	0	0,000021702	0	0,000039416	0,0001286
0	0	0	0,000817442	0	0	0
0	0	0	0	0,000212586	0,000013644	0
0	0,000132349	0,000008448	0,000094042	0	0,00009096	3,428E-05
0	0	0	0,00061489	0,000115956	0	0
0	0	0,00038016	0	0	0,000442672	0
0,000079488	0	0,000033792	0,000094042	0	0	5,999E-05
0	0	0,000768768	0	0	0	0
0	0	0	0	0,000241575	0	1,714E-05
0	0,00017885	0,000016896	0,00014468	0	0,000039416	0,0001714
0	0	0	0	0	0,000559404	8,57E-06
0,000056448	0	0	0,000528082	0	0	0
0,00001152	0,000042924	0,000008448	0,000028936	0	0,000051544	0,00012
0	0	0	0	0,000135282	0	0
0	0	0	0,000745102	0	0	0
0	0	0,000008448	0,000007234	0,000173934	0,000013644	0
0	0	0	0	0	0	0
0,000062208	0	0	0	0	0	1,714E-05
0	0	0	0	0	0	5,999E-05
0	0	0	0,000376168	0	0	0,0000857
0	0	0	0,000007234	0,000009663	0	5,999E-05
0	0	0	0	0,000241575	0	0
0	0	0	0	0	0	1,714E-05
0,00002304	0	0	0	0	0	0
0	0	0	0	0	0	0
0	0	0	0,000462976	0	0	8,57E-06
0	0	0	0,000007234	0	0	0
0	0	0	0	0	0	0
0,00002304	0	0	0,000101276	0	0	0,0000857
0	0,000042924	0,000008448	0,000007234	0	0,000025772	0,0001714
0	0	0	0,000643826	0	0	0
0,00000576	0	0	0,000094042	0	0	8,57E-06
0	0	0,000033792	0,000130212	0	0,000039416	7,713E-05
0	0,000042924	0,000016896	0,000311062	0,000009663	0,000039416	5,142E-05
0	0	0	0	0	0	0

0	0	0	0	0	0,000013644	0
0	0	0	0,000658294	0	0	0
0,00001152	0	0,000008448	0,00007234	0	0,000013644	0,0000857
0	0	0,000008448	0	0	0	1,714E-05
0	0,000089425	0,000016896	0,000209786	0	0	0,0001028
0	0	0	0,000028936	0	0	0
0	0	0	0	0	0,000650364	0
0	0	0	0,000007234	0	0	0
0	0	0	0,000007234	0	0	0
0	0	0	0,00003617	0	0	1,714E-05
0	0	0	0	0	0,00063672	0
0,00001728	0	0	0,000028936	0	0	8,57E-06
0	0	0	0,000007234	0	0	8,57E-06
0	0,000221774	0	0,000014468	0	0	0
0	0	0,000008448	0,000028936	0	0,000039416	0,0001028
0	0	0,00033792	0	0	0,000104604	0
0	0,000042924	0	0	0	0	0
0,00000576	0,000042924	0,000008448	0,000028936	0	0,000013644	0,0000857
0	0,000089425	0,000008448	0,000079574	0,000009663	0,000025772	2,571E-05
0	0	0	0,000007234	0	0	0
0	0	0	0,000332764	0	0	8,57E-06
0	0	0	0	0,000067641	0	3,428E-05
0	0	0	0	0	0	8,57E-06
0	0	0	0,000050638	0	0	0
0	0	0,000582912	0	0	0	0
0	0	0	0,000021702	0,000173934	0	8,57E-06
0	0	0,000016896	0,000057872	0	0	6,856E-05
0	0	0	0	0	0	0
0	0	0,00004224	0,000007234	0,000028989	0,000025772	5,999E-05
0	0	0	0	0	0	0
0	0	0	0	0	0	0
0	0	0	0	0	0,000025772	0,0001628
0	0	0	0	0	0	0
0	0	0	0,000028936	0,000009663	0	0
0	0	0	0,000057872	0	0	0,0002828
0	0	0	0	0	0	0
0	0,000042924	0,000456192	0,000007234	0	0	3,428E-05
0	0	0	0	0	0,000013644	8,57E-06
0	0	0	0,000050638	0	0	0
0	0	0	0	0	0	0
0	0	0	0,000014468	0	0	4,285E-05
0	0	0	0,000065106	0	0	0
0,00000576	0	0	0,000405104	0	0	0
0	0	0	0	0	0	0
0	0	0	0	0	0,00050786	0
0	0	0	0	0	0	0
0	0,000042924	0	0,000007234	0	0,000013644	2,571E-05
0	0	0	0,000014468	0	0	0
0	0	0	0,000007234	0	0,000013644	0
0	0	0	0	0	0	0
0,00001152	0	0,000016896	0,00007234	0	0	0
0,00001152	0	0	0,000014468	0	0	0
0	0	0	0	0	0	0
0,0004896	0	0	0	0	0	0
0	0	0	0	0	0,000077316	0

0	0,000042924	0	0,000021702	0,000009663	0	0,0001714
0	0	0	0,000050638	0	0,000013644	8,57E-06
0,00003456	0	0	0	0	0	4,285E-05
0	0	0	0,000050638	0	0	5,999E-05
0	0	0	0	0	0	0
0	0	0	0,000007234	0	0	4,285E-05
0	0	0	0,00003617	0	0	0
0	0	0	0	0	0,000403256	8,57E-06
0	0,000042924	0,000016896	0,000086808	0	0,000025772	5,999E-05
0	0	0	0	0	0	0
0	0	0	0	0	0	0
0	0	0	0,000007234	0	0	0
0,00000576	0	0	0,000021702	0	0	7,713E-05
0	0	0	0	0	0	0
0	0	0,000016896	0,000007234	0	0	8,57E-06
0,00001152	0	0	0	0	0	0
0	0	0	0,000455742	0	0	0
0	0	0	0,000007234	0	0	3,428E-05
0	0	0	0,000007234	0,000009663	0,000013644	5,999E-05
0	0	0,000016896	0,000050638	0	0,000013644	3,428E-05
0,00000576	0,000132349	0	0,00018085	0	0	0
0	0	0	0	0	0	0
0	0	0	0,000028936	0	0	0
0	0	0	0,000014468	0	0,000039416	0,0000857
0	0	0	0	0	0,000025772	8,57E-06
0	0	0,000008448	0,000007234	0	0	0
0	0,000132349	0,000016896	0,000021702	0	0	1,714E-05
0	0	0	0	0	0,000051544	3,428E-05
0	0,000089425	0	0,000014468	0	0,000013644	3,428E-05
0	0	0	0,000007234	0,000038652	0	0
0	0	0	0,000028936	0	0	0
0,00001152	0	0	0,000007234	0	0,000013644	4,285E-05
0	0	0	0,000007234	0	0	0
0,00003456	0	0	0	0	0,000013644	0
0,00001728	0	0	0	0	0	3,428E-05
0	0	0	0	0	0	0
0	0	0	0,000007234	0	0	0
0,00000576	0	0	0	0	0,000013644	2,571E-05
0	0,000042924	0	0	0	0	0
0	0,000089425	0	0,000021702	0,000009663	0	4,285E-05
0	0	0	0	0,000038652	0	0
0	0	0	0,000014468	0	0,000013644	2,571E-05
0	0	0,000405504	0	0	0	0
0	0	0	0,000028936	0	0	3,428E-05
0	0	0,000016896	0,000021702	0	0	7,713E-05
0	0	0,000135168	0,000014468	0	0	0
0,00004608	0	0,00004224	0,000014468	0	0	0
0	0	0	0	0	0	0
0	0,000042924	0	0	0	0,000260752	8,57E-06
0	0	0	0,000057872	0	0,000013644	3,428E-05
0	0	0	0,000021702	0	0,000013644	8,57E-06
0,00000576	0	0	0,000079574	0	0,000013644	1,714E-05
0	0	0	0,000007234	0	0	0
0	0,000042924	0,000033792	0,000021702	0	0,000025772	0,00018
0	0	0,000008448	0,000007234	0	0	3,428E-05

0	0	0	0	0	0	3,428E-05
0	0	0,00033792	0,000014468	0	0	0
0	0	0,0002112	0	0	0	0
0	0	0	0	0,000106293	0	8,57E-06
0	0	0	0	0	0,000298652	7,713E-05
0	0	0	0,000094042	0	0	0,0001714
0	0	0	0,000007234	0	0	0
0	0	0	0,000050638	0	0	1,714E-05
0	0,000042924	0,000008448	0,000028936	0	0	3,428E-05
0	0	0	0,000007234	0	0	8,57E-06
0	0	0,000008448	0,000014468	0	0	2,571E-05
0,000062208	0	0	0,000007234	0	0,000025772	0
0,00000576	0	0	0,000014468	0	0	2,571E-05
0	0,000089425	0	0,000007234	0	0,000013644	4,285E-05
0	0	0	0	0	0	0
0	0	0	0	0	0	0
0	0	0	0	0	0	0
0	0	0	0,00007234	0	0	0
0	0	0	0	0	0	0
0,00000576	0	0	0,000028936	0,000009663	0,000039416	5,142E-05
0	0	0,000008448	0,000021702	0	0	0
0	0,000042924	0	0	0	0	5,142E-05
0	0	0	0	0	0	0
0	0	0	0,000079574	0	0	4,285E-05
0	0	0	0	0	0	0
0	0	0	0,000050638	0	0	2,571E-05
0	0	0,000008448	0,000057872	0	0,000013644	1,714E-05
0	0	0	0	0	0	0
0	0	0	0,00003617	0	0	7,713E-05
0	0	0	0,000086808	0,000028989	0	8,57E-06
0	0	0,000008448	0	0	0	0,0000857
0,00004608	0	0	0	0	0,000025772	8,57E-06
0	0	0,000016896	0,000021702	0	0,000025772	1,714E-05
0	0	0	0,000014468	0	0	8,57E-06
0	0	0	0	0,000154608	0	0
0,00003456	0	0	0	0	0	0
0	0	0	0	0	0,000013644	0
0	0	0	0	0,000077304	0	0
0	0	0	0	0	0	0
0	0	0	0	0	0	0
0	0	0	0	0	0	0
0	0	0	0	0	0	0
0	0	0	0	0	0	0
0	0	0	0	0	0,000312296	0
0	0	0	0	0	0	0
0	0	0	0	0,000028989	0	0
0	0	0	0	0	0	0
0	0	0	0	0	0,000013644	0
0,00001152	0	0,00004224	0	0	0	0
0	0	0	0	0	0	0
0	0	0	0	0	0	0
0	0	0	0,000094042	0,000009663	0	0
0	0	0	0,000007234	0	0	0
0	0,000042924	0	0	0	0,000013644	8,57E-06
0	0	0	0	0	0	0

0	0	0	0	0	0,000025772	0
0	0	0	0	0,000048315	0	0
0	0	0	0,000267658	0	0	0
0	0	0	0,000014468	0	0,000025772	5,142E-05
0	0	0	0	0	0	0
0	0	0	0	0	0	0
0	0	0,000135168	0	0	0	0
0	0	0	0	0	0	0
0	0	0	0,000014468	0,000028989	0,000013644	8,57E-06
0	0	0	0	0,000038652	0	0
0	0	0	0,000021702	0	0	0
0	0	0	0	0	0	8,57E-06
0	0	0	0	0,000067641	0	0
0,00000576	0	0	0,00003617	0	0,000013644	2,571E-05
0	0	0	0	0,000038652	0,00009096	4,285E-05
0	0	0	0	0	0	0
0	0	0	0	0	0,000025772	0
0,00001728	0	0	0,000007234	0	0,000039416	0
0	0	0	0,000028936	0	0	0
0	0	0,000008448	0,000007234	0	0	8,57E-06
0,00004032	0	0	0	0	0	0
0,00000576	0,000042924	0	0,000007234	0	0,000013644	2,571E-05
0	0	0	0,000050638	0	0	9,427E-05
0	0,000042924	0	0	0	0	1,714E-05
0,0000288	0	0	0	0,000009663	0,000077316	0
0	0	0	0	0	0	0
0	0	0	0,000028936	0	0,000013644	1,714E-05
0	0	0	0	0,000144945	0,000013644	0
0	0	0	0,000007234	0	0	0
0	0	0	0	0	0	0
0	0	0	0,000014468	0,000019326	0	7,713E-05
0	0	0,000008448	0,00003617	0	0	8,57E-06
0	0	0	0	0	0	0
0	0	0,00008448	0	0	0	0
0	0,000042924	0	0,00003617	0	0,000013644	8,57E-06
0	0	0	0,000007234	0,000048315	0	3,428E-05
0	0	0	0,000007234	0	0	0
0	0	0	0,000079574	0	0	0
0	0	0	0	0	0	0
0	0,000089425	0,000008448	0,000028936	0	0	2,571E-05
0	0,000042924	0,000008448	0,00003617	0	0	0
0	0	0	0	0	0	0
0	0	0	0	0	0	3,428E-05
0,00000576	0,000042924	0,000008448	0	0	0	0,0001286
0	0	0	0	0	0	8,57E-06
0	0	0	0,000086808	0	0	8,57E-06
0	0	0	0	0	0	0
0	0	0	0	0	0	0
0	0	0	0	0	0	0
0	0	0	0	0	0	0
0	0	0	0	0	0	0
0,00000576	0	0	0	0	0	2,571E-05
0	0	0	0	0	0	8,57E-06
0	0	0	0	0	0,000025772	0
0	0	0	0	0	0	0

0	0,000042924	0	0,000057872	0	0	8,57E-06
0	0	0	0	0,000048315	0,000051544	2,571E-05
0	0	0	0	0,000009663	0	0
0	0	0	0	0	0	0
0	0	0	0	0	0	0
0	0,00017885	0,000016896	0	0	0	8,57E-06
0	0	0	0,000007234	0	0	0
0	0	0	0,000007234	0,000009663	0	0
0	0	0	0,000014468	0	0,00009096	8,57E-06
0	0	0	0	0	0	8,57E-06
0	0	0	0,000028936	0	0,000013644	4,285E-05
0	0,000042924	0,000008448	0	0	0	8,57E-06
0	0	0	0	0	0	0
0	0,000132349	0	0,000050638	0	0,000013644	1,714E-05
0	0	0	0,00003617	0	0	0
0	0	0,000008448	0,000007234	0	0,000025772	8,57E-06
0	0	0,000008448	0,00003617	0	0	0
0	0	0	0,000014468	0	0	1,714E-05
0	0	0	0	0	0	0
0	0	0	0,000057872	0	0	8,57E-06
0	0,000042924	0	0,000007234	0	0	8,57E-06
0,00002304	0	0	0	0	0	8,57E-06
0,00001728	0,000042924	0	0,000007234	0	0	3,428E-05
0	0	0	0	0	0	0
0	0	0	0	0	0,000013644	8,57E-06
0	0	0	0,000021702	0	0	0
0,00000576	0	0	0,000028936	0	0	5,142E-05
0	0	0	0,000021702	0,000009663	0,000013644	2,571E-05
0	0,000042924	0,000008448	0	0	0	0
0	0	0	0	0	0	0
0	0	0	0	0,000009663	0	1,714E-05
0,00000576	0	0	0	0	0	8,57E-06
0	0	0	0,000028936	0	0	0
0	0	0	0	0,000028989	0	0
0	0	0	0	0,000019326	0	8,57E-06
0,00000576	0	0,000008448	0	0	0	0
0	0	0	0	0	0	0
0	0	0	0,000007234	0	0	0
0	0	0	0	0	0	0
0	0,000042924	0	0	0	0	0
0	0	0	0	0	0,000025772	0
0	0	0	0,000007234	0	0,000013644	0
0	0	0	0	0	0	0
0	0	0	0	0	0	0
0	0	0	0	0,000009663	0	8,57E-06
0	0	0	0,00010851	0	0	0
0	0	0	0	0	0	0
0	0	0	0,00003617	0	0	1,714E-05
0	0	0	0,000050638	0	0,000013644	7,713E-05
0	0	0	0	0	0	0
0	0	0,000016896	0,00007234	0	0	3,428E-05
0	0	0	0	0	0	0
0	0	0	0	0	0	0
0	0	0,000008448	0	0	0	0
0	0	0,000008448	0	0	0	8,57E-06

0	0	0	0	0	0	0
0	0	0	0	0	0	0
0	0	0	0,000014468	0	0	8,57E-06
0,000073728	0	0	0	0	0	0
0	0,000042924	0	0,000014468	0	0	1,714E-05
0,00002304	0	0	0	0	0,000013644	0
0	0	0	0	0	0	0
0,00001152	0	0	0	0	0	7,713E-05
0	0	0	0,000021702	0	0	0
0	0	0	0,000014468	0	0	0
0	0	0	0,000028936	0	0	0
0	0	0,000016896	0	0	0	8,57E-06
0	0	0	0	0	0,000142504	8,57E-06
0,00000576	0	0	0,000007234	0	0	8,57E-06
0	0	0	0	0	0	0
0	0	0	0,000014468	0	0	5,142E-05
0,00001728	0,000042924	0	0	0	0	0
0	0,000042924	0,000008448	0	0	0	2,571E-05
0	0	0	0	0	0	3,428E-05
0,00001152	0	0	0	0	0	0
0	0	0	0,000021702	0	0	0
0,00002304	0	0	0	0	0	4,285E-05
0	0	0	0,000007234	0	0	8,57E-06
0	0,000042924	0,000008448	0,000014468	0	0	2,571E-05
0	0	0	0,000007234	0	0	0
0	0	0	0	0	0	6,856E-05
0	0,000042924	0	0,000007234	0,000009663	0,000025772	0
0	0	0	0	0	0	0
0	0	0	0,000007234	0	0,000013644	1,714E-05
0	0	0	0	0	0,000013644	8,57E-06
0	0	0	0	0,000028989	0	0
0	0	0	0	0	0	0
0	0	0	0,000159148	0	0	0
0	0	0	0	0	0	0
0	0	0	0	0	0	0
0	0	0,000118272	0,000007234	0	0,000013644	8,57E-06
0	0	0	0	0	0	0
0	0	0	0	0	0	0
0	0	0	0	0	0	0,0001543
0	0	0	0	0	0	0
0	0	0	0	0	0	3,428E-05
0,00003456	0	0	0	0	0	0
0	0	0	0	0	0	0
0	0	0	0,000050638	0	0	1,714E-05
0	0	0	0	0	0	0
0	0	0	0,000014468	0	0	0
0	0	0	0	0	0	0
0	0	0	0	0	0	0
0	0	0	0	0	0	0
0,00001152	0	0	0,000028936	0	0	1,714E-05
0	0	0	0	0	0	0
0	0	0,000008448	0,000007234	0	0	0
0	0	0	0,000014468	0	0	0
0	0	0	0,000057872	0	0	0
0	0,000042924	0	0,000014468	0	0	1,714E-05

0	0,000042924	0	0,00007234	0,000009663	0,000013644	8,57E-06
0	0	0	0	0,000009663	0	0
0,000125568	0	0	0	0	0	8,57E-06
0	0	0	0,000014468	0,000009663	0	8,57E-06
0	0	0	0	0	0	0
0	0	0	0	0	0	8,57E-06
0	0	0	0,000007234	0	0	8,57E-06
0	0	0	0	0	0	0
0	0	0	0,000007234	0	0	0
0	0,000042924	0	0,000007234	0	0	0
0	0	0	0	0	0,000142504	0
0	0	0	0,000021702	0	0	8,57E-06
0	0	0	0,000007234	0	0,000013644	8,57E-06
0	0	0	0	0	0	0
0	0	0	0,000007234	0,000009663	0	3,428E-05
0	0	0	0,000021702	0	0	5,142E-05
0	0	0	0	0,000048315	0	0
0	0	0	0,000007234	0,000019326	0,000013644	0
0	0	0	0,000137446	0	0	0
0	0	0	0	0	0	0
0	0	0	0	0	0	0
0	0	0	0	0	0,000013644	0
0	0	0	0,000007234	0	0	8,57E-06
0	0	0,000008448	0,000007234	0	0	8,57E-06
0	0	0	0,000028936	0	0	1,714E-05
0	0	0	0	0	0	0
0	0	0	0,000086808	0	0	8,57E-06
0	0	0	0,00003617	0	0	8,57E-06
0	0	0	0	0	0	0
0	0	0	0,000014468	0	0	3,428E-05
0	0	0	0	0	0,000130376	0
0	0	0	0	0,000019326	0	0
0	0	0	0,000101276	0	0	0
0	0	0	0	0	0	8,57E-06
0	0	0	0,000007234	0	0	5,142E-05
0	0	0	0	0	0	0
0	0	0	0,000007234	0	0	8,57E-06
0,00004032	0	0	0	0	0	0
0	0,000042924	0	0,000007234	0,000009663	0,000013644	8,57E-06
0	0	0	0	0	0	0
0	0	0	0	0	0	0
0	0	0	0	0	0	0
0	0	0	0	0	0,000013644	0
0	0,000089425	0	0,000007234	0	0	8,57E-06
0	0	0	0	0	0	0
0	0	0	0	0	0	0
0	0	0	0	0	0	0
0,00000576	0	0	0,000007234	0	0	0
0	0	0	0	0	0	0
0,00000576	0	0,000008448	0,000021702	0	0,000013644	8,57E-06
0	0	0	0	0	0	8,57E-06
0	0,000042924	0	0,000021702	0	0,000013644	0
0	0	0,000016896	0	0	0	8,57E-06
0	0	0	0	0	0	0
0	0	0	0,000007234	0	0,000013644	1,714E-05

0	0	0	0	0	0	0
0	0	0	0	0	0	0
0	0	0	0	0	0,000116732	0
0	0	0	0	0,000009663	0,000013644	0
0	0	0	0	0	0	0
0	0	0	0,000115744	0	0	0
0	0	0	0,000007234	0	0	0
0	0	0	0	0	0	0
0	0	0	0,000021702	0	0	0
0	0	0	0,000028936	0	0	0
0	0	0	0	0	0	0
0	0	0	0	0	0	0
0	0	0	0	0	0	0
0	0	0	0	0	0	0
0	0	0	0,000057872	0	0,000051544	0
0,00000576	0	0	0,000007234	0	0	0
0,00000576	0	0	0,000014468	0	0	0
0	0	0	0,00007234	0	0	0
0	0	0	0	0	0,000013644	0
0	0	0	0	0	0	0
0	0	0	0	0,000009663	0	0
0	0	0,000008448	0,000050638	0	0	8,57E-06
0,00000576	0	0	0	0	0	0
0	0	0	0	0,000086967	0	0
0	0	0	0	0	0	0
0	0	0	0	0	0	0
0	0	0,000016896	0,000028936	0,000009663	0	0
0	0	0	0	0	0	0
0	0	0	0,000021702	0	0	8,57E-06
0	0	0	0	0	0	0
0	0	0	0,000007234	0	0,000013644	8,57E-06
0	0	0	0	0	0	0
0	0	0,000101376	0	0	0	0
0	0	0	0	0,000086967	0	0
0	0	0	0	0	0	0
0	0	0	0	0	0	0
0	0,000042924	0	0	0	0	8,57E-06
0	0	0	0	0	0	0
0	0	0	0,000007234	0	0	1,714E-05
0	0	0	0	0	0	0
0	0	0	0,000028936	0	0	0
0	0	0,000016896	0,00007234	0	0	0
0	0	0	0,000007234	0	0	8,57E-06
0	0	0	0,000050638	0	0	0
0	0	0	0,000007234	0	0	4,285E-05
0	0	0	0	0,000009663	0	0
0	0	0	0	0,000028989	0	0
0	0	0	0	0	0	0
0	0	0,000033792	0	0,000009663	0,000013644	0
0	0	0	0	0	0	0
0	0	0	0	0,000009663	0	0
0	0	0,000008448	0	0	0	0
0	0	0	0	0	0,000013644	1,714E-05
0	0	0,000008448	0	0	0	1,714E-05
0	0	0	0,000094042	0	0	0

0	0	0	0,000028936	0	0	8,57E-06
0	0	0	0	0	0	4,285E-05
0	0	0	0,000014468	0	0	0
0	0	0	0	0	0	0
0	0	0	0	0	0	0
0	0	0	0,000014468	0	0	2,571E-05
0	0	0	0	0	0	0
0,000073728	0	0	0,000007234	0	0	0
0	0	0	0,000007234	0	0,000013644	0
0	0	0	0	0	0	0
0	0	0	0,000007234	0	0	0
0	0,000089425	0	0	0	0	0
0	0	0	0	0	0	0
0	0	0	0,000007234	0	0	0
0	0	0	0	0	0	0
0	0	0	0	0	0	0
0	0	0	0,000028936	0	0	0
0	0	0	0,000086808	0	0	0
0	0	0	0,000007234	0	0,000013644	1,714E-05
0	0	0	0	0	0	0,0000857
0	0	0,000008448	0,000021702	0	0	0
0	0	0	0,000028936	0	0	0
0	0	0	0	0	0	8,57E-06
0	0,000042924	0	0	0,000009663	0	0
0	0,000042924	0	0,000007234	0	0	8,57E-06
0	0	0	0	0	0	0
0	0	0	0	0	0	0
0	0	0	0	0	0	5,999E-05
0	0	0,000008448	0,00003617	0	0	0
0	0	0	0	0	0	0
0	0	0	0,000028936	0	0	0
0	0	0	0,000028936	0	0	0
0	0	0	0	0	0	0
0	0	0	0	0	0	0
0	0	0,000008448	0	0,000009663	0	8,57E-06
0	0,000042924	0	0	0,000009663	0	8,57E-06
0	0	0	0	0	0	0
0	0	0	0	0	0	0
0	0	0	0,000007234	0	0	0
0	0	0	0,000007234	0	0	8,57E-06
0,00001728	0	0	0,000021702	0	0	8,57E-06
0	0	0,000008448	0,000014468	0	0	0
0	0	0	0,000007234	0,000019326	0,000013644	0
0	0	0	0	0	0	0
0	0	0	0,000007234	0	0,000013644	0
0	0	0	0,000007234	0	0	0
0	0	0,000067584	0	0	0	0
0	0	0	0	0	0	0
0	0,000042924	0,000008448	0	0	0	0
0	0	0	0	0	0	0
0	0	0	0,000007234	0	0	0
0	0	0	0	0	0	0
0	0	0	0,000065106	0	0	0
0	0	0	0,000007234	0,000038652	0	8,57E-06
0	0	0	0	0	0	0

0	0	0	0	0	0	0
0	0	0	0	0,000009663	0,000013644	0
0	0	0	0	0	0	0
0	0	0	0,000007234	0	0	0
0	0	0	0,000014468	0	0	0
0	0	0	0	0	0	0
0,00001728	0	0	0,000007234	0,000009663	0,000013644	0
0	0	0	0	0	0,000013644	8,57E-06
0	0	0	0	0	0	0
0	0	0	0	0	0	0
0	0	0	0	0,000009663	0	0
0	0	0	0	0	0	0
0	0	0	0,000007234	0	0	0
0	0,000042924	0,000008448	0	0	0	8,57E-06
0	0,000042924	0	0	0	0	0
0	0	0	0	0	0	0
0	0	0	0,000057872	0,000009663	0	0
0	0	0	0	0	0	0
0,000062208	0	0	0	0	0	0
0	0	0	0	0,000028989	0,000013644	0
0	0	0	0	0	0,000025772	1,714E-05
0	0	0	0	0	0	0
0	0	0	0	0	0	0
0	0	0	0	0	0	0
0	0	0	0	0	0,000065188	0
0	0	0	0	0	0	0
0	0	0	0	0	0	4,285E-05
0	0	0	0,000007234	0	0	0
0,00001152	0	0	0,000007234	0	0	0
0	0	0	0	0	0	0
0	0	0	0	0	0	0
0	0	0	0,000007234	0	0,000013644	0
0	0	0	0,000007234	0	0	0
0	0	0	0,000007234	0	0	0
0	0,000042924	0	0	0,000019326	0	0
0	0	0	0	0,000009663	0	0
0	0	0	0,000007234	0	0	0
0	0	0	0,000007234	0	0	8,57E-06
0	0	0	0	0	0	0
0	0	0	0	0	0	5,999E-05
0	0	0	0	0	0	0
0	0	0	0	0	0	8,57E-06
0	0	0	0,000007234	0	0	3,428E-05
0	0	0	0	0	0	0
0	0	0	0,000021702	0	0	0
0	0	0	0	0	0	0
0	0	0	0	0	0	0
0	0	0	0,000007234	0	0	5,142E-05
0	0	0	0	0	0	0
0	0	0	0	0	0,000013644	1,714E-05
0	0	0	0	0	0	0
0	0	0	0,000007234	0	0,000013644	8,57E-06
0	0	0	0,000057872	0	0	0
0	0	0	0	0	0	0
0	0	0	0,000021702	0	0	1,714E-05

0	0	0	0	0	0	8,57E-06
0	0	0	0	0,000009663	0	0
0,00001152	0	0	0	0	0	0
0	0	0	0	0	0	1,714E-05
0	0	0,000008448	0	0,000009663	0	0
0	0	0	0	0	0	0
0	0	0,000008448	0	0	0	2,571E-05
0	0	0	0	0	0	0
0	0	0	0	0	0	8,57E-06
0	0	0,000008448	0	0	0	8,57E-06
0	0	0	0	0	0	0
0,00002304	0	0	0	0	0	0
0	0	0	0	0	0	0
0	0	0	0	0	0	0
0	0	0	0	0	0	0
0	0	0	0	0	0	0
0	0	0	0	0,000009663	0	0
0	0	0	0	0	0	0
0	0	0	0	0	0	0
0	0	0	0,000007234	0,000028989	0	0
0	0	0	0	0	0	0
0	0	0	0	0	0	0
0	0	0	0	0,000019326	0	0
0	0	0	0	0	0	0
0	0	0	0	0	0	0
0	0	0	0	0	0	0
0	0	0	0	0	0	0
0	0	0	0	0	0	0
0	0	0	0	0	0	3,428E-05
0	0	0	0,000028936	0	0,000013644	0
0,00001152	0	0,000008448	0	0	0,000013644	0
0	0	0	0,000028936	0	0	8,57E-06
0	0	0	0	0	0	0
0	0	0	0	0	0	5,142E-05
0	0	0	0,000014468	0	0	8,57E-06
0	0	0	0,000007234	0,000009663	0	8,57E-06
0	0	0,00004224	0	0	0	0
0	0	0	0,000014468	0	0	0
0	0	0	0	0	0	0
0	0	0	0,000050638	0	0	0
0	0	0	0,000050638	0	0	0
0	0	0	0	0	0	0
0	0	0	0	0	0	8,57E-06
0	0	0	0	0	0	0
0	0	0	0	0	0	0
0	0,000042924	0	0,000007234	0	0	0
0	0,000042924	0	0,000007234	0	0	0
0	0	0	0	0	0	0
0	0	0	0	0	0	0
0	0	0	0	0	0,000013644	0
0	0	0	0	0	0	0
0	0	0	0,000007234	0	0	0
0	0	0	0,000007234	0	0	0
0	0	0	0	0	0	0
0	0	0	0	0	0	8,57E-06

0	0	0	0	0	0	0
0	0	0	0	0	0	0
0	0	0,000008448	0	0	0	0
0	0	0	0	0	0	0
0	0	0	0	0	0	8,57E-06
0	0	0	0	0	0	0
0	0	0	0,000014468	0,000009663	0	8,57E-06
0	0	0	0	0	0	0
0	0	0	0	0	0	0
0	0	0	0	0	0	0
0,00004608	0	0	0	0	0	0
0,00004608	0	0	0	0	0	0
0	0	0,000008448	0	0	0,000013644	0
0	0	0	0	0	0	0
0	0	0	0	0	0,000013644	8,57E-06
0	0	0	0	0	0	0
0	0	0	0	0	0	0
0	0	0	0,000007234	0	0	1,714E-05
0	0	0	0	0,000009663	0	0
0	0	0	0	0,000009663	0	0
0	0	0	0	0	0	0
0	0	0	0	0	0	0
0	0	0	0	0,000009663	0	0
0	0	0,000008448	0	0	0	0
0	0	0	0	0	0	0
0	0,000042924	0	0	0	0	0
0	0,000042924	0	0	0	0	0
0	0,000042924	0	0	0	0	0
0	0,000042924	0	0	0	0	0
0	0,000042924	0	0	0	0	0
0	0	0	0	0	0	8,57E-06
0	0	0	0	0	0	0
0	0	0	0	0	0	0
0	0	0	0	0	0	0
0	0	0	0	0	0	0
0	0	0,000016896	0,000007234	0	0	8,57E-06
0	0	0	0	0	0	0
0	0	0	0	0	0	0
0	0	0	0	0	0	0
0	0	0	0	0	0,000013644	0
0,00000576	0	0	0,00003617	0	0	0
0	0	0	0	0	0	0
0	0	0	0	0	0	0
0	0	0	0	0	0	0
0	0	0	0	0	0	0
0	0	0	0	0	0	0
0	0	0	0,000021702	0	0	0
0	0	0	0,000007234	0	0	0
0	0	0	0	0	0	0
0	0	0	0	0	0	0
0	0	0	0	0	0,000013644	0
0	0	0	0	0	0,000039416	0
0	0	0	0	0	0	0
0	0	0	0	0	0	8,57E-06
0	0	0	0,000021702	0	0	1,714E-05

0	0	0	0	0	0	1,714E-05
0	0	0	0	0,000028989	0	0
0	0	0	0	0,000009663	0	0
0	0	0,000008448	0,000007234	0	0	0
0,00000576	0	0	0	0	0	0
0	0	0	0	0	0	8,57E-06
0	0	0	0	0,000009663	0	0
0	0	0	0	0	0	0
0	0	0	0	0,000019326	0	0
0	0	0	0	0	0	0
0	0	0	0	0,000009663	0	0
0	0	0	0	0	0	0
0	0	0	0	0	0	0
0	0	0	0	0	0	0
0	0	0	0	0	0	0
0	0	0	0	0	0	0
0	0	0	0	0,000009663	0	0
0	0	0	0	0	0	0
0	0	0	0,00003617	0	0	0
0	0	0	0,00003617	0	0	0
0,00001152	0	0	0	0	0	0
0,00000576	0	0	0,000021702	0	0	0
0	0	0	0,000007234	0	0	0
0	0	0	0,000007234	0	0	0
0	0	0	0,000007234	0	0	0
0	0	0	0	0	0	0
0	0	0	0	0	0	0
0	0	0	0	0	0	0
0,00000576	0	0	0	0	0	0
0	0	0,000008448	0,000007234	0	0	0
0	0	0	0	0	0	0
0	0	0	0,000021702	0	0	0
0	0	0	0	0	0	0
0	0	0	0,000007234	0	0	8,57E-06
0	0	0	0	0	0	0
0	0	0	0	0	0	0
0	0	0	0	0	0	0
0	0	0	0	0	0	0
0	0	0	0	0	0	3,428E-05
0	0	0	0	0	0	0
0	0	0	0,000007234	0	0	1,714E-05
0	0	0	0	0	0	0
0	0	0	0,000014468	0	0	0
0	0	0	0	0	0	0
0	0	0	0,000007234	0	0	0
0	0	0	0	0	0,000013644	0
0	0	0	0	0	0	0
0,00001152	0	0	0	0	0	0
0	0	0	0,000014468	0	0	0
0	0	0	0,000014468	0	0	0
0	0	0	0	0	0,000013644	0
0	0	0	0,000014468	0	0	8,57E-06
0	0	0,000008448	0	0	0	8,57E-06
0	0	0	0,000021702	0	0	0
0	0	0	0	0	0	0

0	0	0	0,000021702	0	0	0
0	0	0	0	0	0	8,57E-06
0	0	0	0	0	0	0
0	0	0	0,000014468	0	0	0
0,00001728	0	0	0	0	0	0
0	0	0	0	0	0	0
0	0	0	0	0	0	0
0	0	0	0	0	0	0
0	0	0	0	0	0	0
0	0	0	0	0	0	0
0	0	0	0	0	0	0
0	0	0	0	0	0	0
0	0	0	0	0	0	0
0	0	0	0	0	0	0
0	0	0	0	0,000028989	0	0
0	0	0	0	0,000028989	0	0
0	0	0	0,000028936	0	0	0
0	0	0	0,000028936	0	0	0
0	0	0	0,000028936	0	0	0
0	0	0	0,000028936	0	0	0
0	0	0	0,000028936	0	0	0
0	0	0	0,000028936	0	0	0
0	0	0	0,000028936	0	0	0
0	0	0	0,000028936	0	0	0
0	0	0	0,000028936	0	0	0
0,0000288	0	0	0	0	0	0
0	0	0	0	0	0	0
0	0	0	0	0,000009663	0	0
0	0	0	0	0	0	0
0	0	0	0	0	0	0
0	0	0	0	0	0	0
0	0	0	0	0	0	0
0	0	0	0	0	0	0
0	0	0	0	0	0	0
0	0	0	0	0	0	0
0	0	0	0	0	0	0
0	0	0	0	0	0	0
0	0	0	0	0	0	0
0	0	0	0	0	0	0
0	0	0	0	0	0	0
0	0	0	0	0	0	0
0	0	0	0	0	0	0
0	0	0	0	0	0	0
0	0	0	0	0	0	0
0	0	0	0	0	0	0
0	0	0	0	0	0	0
0	0	0	0	0	0	0
0	0	0	0	0	0	0
0	0	0	0	0	0	0
0	0	0	0	0	0	0
0	0	0	0	0,000009663	0	0
0	0	0	0	0	0	0
0	0	0	0	0	0	0
0	0	0	0	0	0	0
0	0	0	0	0,000009663	0	0

0	0	0	0	0,000009663	0	0
0	0	0	0	0	0	0
0	0	0	0	0	0	0
0	0	0	0	0	0	0
0	0	0	0	0	0	0
0	0	0,000008448	0	0	0	0
0	0	0	0	0	0	0
0	0	0	0	0	0	0
0	0	0	0	0	0	0
0	0	0	0	0	0	0
0	0	0	0	0	0	0
0	0	0	0	0	0	0
0	0	0	0,000014468	0	0	0
0	0	0	0	0	0	0
0	0	0	0	0	0	0
0	0	0	0	0	0	0
0	0	0	0,000007234	0	0	0
0	0	0	0,000007234	0,000009663	0	0
0	0	0	0	0	0	0
0	0	0	0	0	0	0
0	0	0	0	0	0,000025772	0
0	0	0	0	0	0,000025772	0
0	0	0	0	0	0	0
0	0	0	0	0	0	2,571E-05
0	0	0	0,000007234	0	0	0
0	0	0	0,000007234	0	0	0
0	0	0	0	0,000009663	0	0
0	0	0	0	0	0	0
0	0	0	0	0	0	0
0	0	0	0	0	0	0
0	0	0	0,000007234	0	0	0
0,00000576	0	0	0	0,000009663	0	0
0	0	0	0,000007234	0	0	8,57E-06
0	0	0	0,000007234	0	0	8,57E-06
0	0	0	0,000007234	0	0	8,57E-06
0	0	0	0,000007234	0	0	8,57E-06
0	0	0	0	0	0	0
0	0	0	0,000014468	0	0	0
0	0	0	0,000007234	0	0	0
0	0	0	0,000007234	0	0	8,57E-06
0	0	0	0,000007234	0	0	1,714E-05
0	0	0	0,000014468	0,000009663	0	0
0	0	0	0	0	0	0
0	0	0	0	0	0	0
0	0	0	0	0	0	0
0	0	0	0	0	0	0
0	0	0	0	0	0	0
0	0	0	0	0	0	0
0	0	0	0	0	0	0
0	0	0	0	0	0	0
0	0	0	0	0	0	0
0,00001152	0	0	0	0	0	0
0	0	0	0	0	0	0
0	0	0	0	0	0	0
0	0	0	0	0	0	0

0	0	0	0	0	0	0
0	0	0	0,000014468	0	0	0
0	0	0	0	0	0	0
0	0	0	0	0	0	0
0	0	0	0	0	0,000013644	0
0	0	0	0	0	0	8,57E-06
0	0	0	0	0	0	8,57E-06
0	0	0	0	0	0	8,57E-06
0	0	0	0	0	0	0
0	0	0	0	0	0,000013644	0
0	0	0	0	0	0,000013644	0
0	0	0	0	0	0,000013644	0
0	0	0	0	0	0	0
0	0	0,000008448	0	0	0	0
0	0	0	0	0	0	0
0	0	0,000008448	0	0	0,000013644	0
0	0	0	0	0	0	0
0,00001152	0	0	0	0	0	0
0	0	0	0,000021702	0	0	0
0	0	0	0,000021702	0	0	0
0	0	0	0,000021702	0	0	0
0	0	0	0,000021702	0	0	0
0	0	0	0,000021702	0	0	0
0	0	0	0,000021702	0	0	0
0	0	0	0	0	0	0
0	0	0	0	0	0	0
0	0	0	0	0	0	0
0	0	0	0	0	0	0
0	0	0	0	0	0	0
0	0	0	0	0	0	0
0	0	0	0	0	0	0
0	0	0	0	0	0	0
0	0	0	0	0	0	0
0	0	0	0	0	0	0
0	0	0	0	0	0	0
0	0	0	0	0	0	0
0	0	0	0	0	0	0
0	0	0	0	0	0	0
0	0	0	0	0	0	0
0	0	0	0	0,000009663	0	0
0	0	0	0	0	0	0
0	0	0	0	0	0	0
0	0	0	0	0	0	0
0	0	0	0	0,000019326	0	0
0	0	0	0	0,000019326	0	0
0	0	0	0	0	0	0
0	0	0	0	0	0	0
0	0	0	0	0	0	0
0	0	0	0	0	0	0
0	0	0	0	0	0	0
0	0	0	0	0	0	0
0	0	0	0	0	0	0
0	0	0	0	0,000009663	0	0
0	0	0	0	0,000009663	0	0
0	0	0	0	0	0	0

0	0	0	0	0	0	0
0	0	0	0	0	0	0
0	0	0	0	0	0	0
0	0	0	0	0	0	0
0	0	0	0	0	0	0
0	0	0	0	0	0	0
0	0	0	0	0,000009663	0	0
0	0	0	0	0	0	0
0	0	0	0	0	0	0
0	0	0	0	0	0	0
0	0	0	0	0	0	0
0	0	0	0	0	0	0
0	0	0	0	0	0	0
0	0	0	0	0	0	0
0	0	0	0	0	0	0
0	0	0	0	0	0	0
0	0	0	0	0	0	0
0	0	0	0	0	0	0
0	0	0	0	0	0	8,57E-06
0	0	0	0	0	0	0
0	0	0	0	0	0	0
0	0	0,000008448	0	0	0	0
0	0	0	0	0,000009663	0	0
0	0	0	0	0,000009663	0	8,57E-06
0	0	0	0	0	0	0
0	0	0	0	0	0	0
0	0	0	0	0	0	0
0	0	0	0	0	0	0
0	0	0	0	0	0	0
0	0	0	0	0	0	0
0	0	0	0	0	0	0
0	0	0	0	0	0	0
0	0	0,000008448	0	0,000009663	0	0
0,00000576	0	0	0	0	0	0
0	0	0	0	0	0	8,57E-06
0	0	0,000008448	0	0	0	0
0	0	0,000008448	0	0	0	0
0	0	0	0	0	0	8,57E-06
0	0	0	0	0	0	8,57E-06
0	0	0	0	0	0	8,57E-06
0	0	0	0	0	0	8,57E-06
0	0	0	0	0	0	0
0	0	0,000008448	0	0	0	0
0	0	0	0,000007234	0	0	0
0	0	0	0	0	0	0
0,00001728	0	0	0	0	0	0
0	0	0	0	0	0	0
0	0	0	0	0	0	0
0	0	0	0	0	0	8,57E-06
0	0	0	0	0	0	1,714E-05
0	0	0,000008448	0	0	0	0
0	0	0,000008448	0	0	0	8,57E-06
0	0	0	0	0	0	0
0	0	0	0	0	0	0
0	0	0	0	0	0	0

0	0	0	0	0	0	0
0	0	0,000016896	0	0	0	0
0	0	0,000016896	0	0	0	0
0	0	0	0,000007234	0	0	0
0	0	0	0,000007234	0	0	0
0	0	0	0,000007234	0	0	0
0	0	0	0	0	0	0
0	0	0	0,000007234	0	0	0
0	0	0	0,000007234	0	0	0
0	0	0	0,000007234	0	0	0
0	0	0	0	0	0	0
0	0	0	0	0	0	0
0	0	0	0,000007234	0	0	0
0	0	0	0,000007234	0	0	0
0	0	0	0,000007234	0	0	0
0	0	0	0,000007234	0	0	0
0	0	0	0	0	0	0
0	0	0	0,000007234	0	0	0
0	0	0	0,000007234	0	0	8,57E-06
0	0	0	0,000007234	0	0	8,57E-06
0	0	0	0,000007234	0	0	8,57E-06
0	0	0	0,000007234	0	0	8,57E-06
0	0	0	0,000007234	0	0	8,57E-06
0	0	0	0,000007234	0	0	8,57E-06
0	0	0	0	0	0	0
0	0	0	0	0	0	0
0	0	0,000008448	0,000007234	0	0	0
0	0	0	0	0	0	0
0	0	0	0	0	0	0
0	0	0	0	0	0	0
0	0	0	0	0	0	8,57E-06
0	0	0	0	0	0	0
0,00000576	0	0	0	0	0	0
0	0	0	0,000014468	0	0	0
0	0	0	0,000014468	0	0	0
0	0	0	0,000014468	0	0	0
0	0	0	0,000014468	0	0	0
0	0	0	0,000014468	0	0	0
0	0	0	0,000014468	0	0	0
0	0	0	0,000014468	0	0	0
0	0	0	0,000014468	0	0	0
0	0	0	0,000014468	0	0	0
0	0	0	0,000014468	0	0	0
0	0	0	0	0	0	0
0	0	0	0	0	0	0
0	0	0	0	0	0	0
0	0	0	0	0	0	0
0	0	0	0	0	0	0
0	0	0	0	0	0	0
0	0	0	0	0	0	0
0	0	0	0	0	0	0
0	0	0	0,000007234	0	0	0
0	0	0	0	0	0	0
0	0	0	0	0	0	0
0	0	0	0	0	0	0

0	0	0	0	0	0	0
0	0	0	0	0	0	0
0	0	0	0	0	0	0
0	0	0	0	0	0	0
0	0	0	0	0	0	0
0	0	0	0	0	0	0
0	0	0	0	0	0,000013644	0
0	0	0	0	0	0,000013644	0
0	0	0	0	0	0,000013644	0
0	0	0	0	0	0,000013644	0
0	0	0	0	0	0,000013644	0
0	0	0	0	0	0,000013644	0
0	0	0	0	0	0,000013644	0
0	0	0	0	0	0,000013644	0
0	0	0	0	0	0,000013644	0
0	0	0	0	0	0,000013644	0
0	0	0	0	0	0	0
0	0	0	0	0	0	0
0	0	0	0	0	0	0
0	0	0	0	0	0	0
0	0	0	0	0	0	0
0	0	0	0	0	0	0
0	0	0	0	0	0	0
0	0	0	0	0	0	0
0	0	0	0	0	0	0
0	0	0	0	0	0	0
0	0	0	0	0	0	8,57E-06
0	0	0	0	0	0	0
0	0	0	0	0	0	0
0	0	0	0	0	0	0
0	0	0	0	0	0	0
0	0	0	0	0	0	0
0,00000576	0	0	0	0	0	0
0	0	0	0	0	0	0
0	0	0	0	0	0	0
0	0	0	0	0	0	0
0	0	0	0	0	0	0
0	0	0	0	0	0	0
0	0	0	0	0	0	0
0	0	0	0	0	0	0
0	0	0	0	0	0	0
0	0	0	0	0	0	0
0	0	0	0	0	0	0
0	0	0	0	0	0	0
0	0	0	0	0	0	0
0	0	0	0	0	0	0
0	0	0	0	0	0	0
0	0	0	0	0	0	0
0	0	0	0	0	0	0
0	0	0	0	0,000009663	0	0
0	0	0	0	0,000009663	0	0
0	0	0	0	0,000009663	0	0
0	0	0	0	0,000009663	0	0
0	0	0	0	0,000009663	0	0
0	0	0	0	0,000009663	0	0

0	0	0	0	0,000009663	0	0
0	0	0	0	0,000009663	0	0
0	0	0	0	0,000009663	0	0
0	0	0	0	0,000009663	0	0
0	0	0	0	0	0	0
0	0	0	0	0	0	0
0	0	0	0	0	0	0
0	0	0	0	0	0	0
0	0	0	0	0	0	0
0	0	0	0	0	0	0
0	0	0	0	0	0	0
0	0	0	0	0	0	0
0	0	0	0	0	0	0
0	0	0	0	0	0	0
0	0	0	0	0	0	0
0	0	0	0	0	0	0
0	0	0	0	0	0	0
0	0	0	0	0	0	0
0	0	0	0	0	0	0
0	0	0	0	0	0	0
0	0	0	0	0	0	0
0	0	0	0	0	0	0
0	0	0	0	0	0	0
0	0	0	0	0	0	0
0	0	0	0	0	0	0
0	0	0	0	0	0	0
0	0	0	0	0	0	0
0	0	0	0	0	0	0
0	0	0	0	0	0	0
0	0	0	0	0	0	0
0	0	0	0	0	0	0
0	0	0	0	0	0	0
0	0	0	0	0	0	0
0	0	0	0	0	0	0
0	0	0	0	0	0	0
0	0	0	0	0	0	0
0	0	0	0	0	0	0
0	0	0	0	0	0	0
0	0	0	0	0	0	0
0	0	0	0	0	0	0
0	0	0	0	0	0	0
0	0	0	0	0	0	0
0	0	0	0	0	0	0
0	0	0	0	0	0	0
0	0	0	0	0	0	0
0	0	0	0	0	0	0
0	0	0	0	0	0	0
0	0	0	0	0	0	0
0	0	0	0	0	0	0
0	0	0	0	0	0	0
0	0	0	0	0	0	0
0	0	0	0	0	0	0
0	0	0	0	0	0	0
0	0	0	0	0	0	0
0	0	0	0	0	0	0

0	0	0	0	0	0	0
0	0	0	0	0	0	0
0	0	0	0	0	0	0
0	0	0	0	0	0	0
0	0	0	0	0	0	0
0	0	0	0	0	0	0
0	0	0	0	0	0	0
0	0	0	0	0	0	0
0	0	0	0	0	0	0
0	0	0	0	0	0	0
0	0	0	0	0	0	0
0	0	0	0	0	0	0
0	0	0	0	0	0	0
0	0	0	0	0	0	0
0	0	0	0	0	0	8,57E-06
0	0	0	0	0	0	8,57E-06
0	0	0	0	0	0	8,57E-06
0	0	0	0	0	0	8,57E-06
0	0	0	0	0	0	8,57E-06
0	0	0	0	0	0	8,57E-06
0	0	0	0	0	0	8,57E-06
0	0	0	0	0	0	8,57E-06
0	0	0	0	0	0	8,57E-06
0	0	0	0	0	0	8,57E-06
0	0	0	0	0	0	8,57E-06
0	0	0	0	0	0	8,57E-06
0	0	0	0	0	0	8,57E-06
0	0	0	0	0	0	8,57E-06
0	0	0	0	0	0	8,57E-06
0	0	0	0	0	0	8,57E-06
0	0	0	0	0	0	8,57E-06
0	0	0	0	0	0	8,57E-06
0	0	0	0	0	0	8,57E-06
0	0	0	0	0	0	8,57E-06
0	0	0	0	0	0	8,57E-06
0	0	0	0	0	0	8,57E-06
0	0	0	0	0	0	8,57E-06
0	0	0	0	0	0	8,57E-06
0	0	0	0	0	0	8,57E-06
0	0	0	0	0	0	8,57E-06
0	0	0	0	0	0	8,57E-06
0	0	0	0	0	0	8,57E-06
0	0	0	0	0	0	0
0	0	0	0	0	0	0
0	0	0	0	0	0	0
0	0	0	0	0	0	0
0	0	0	0	0	0	0
0	0	0	0	0	0	0
0	0	0,000008448	0	0	0	0
0	0	0,000008448	0	0	0	0
0	0	0,000008448	0	0	0	0
0	0	0,000008448	0	0	0	0
0	0	0,000008448	0	0	0	0
0	0	0	0	0	0	0
0	0	0	0	0	0	0

0	0	0	0	0	0	0
0	0	0	0	0	0	0
0	0	0	0	0	0	0
0	0	0	0	0	0	0
0	0	0	0,000007234	0	0	0
0	0	0	0,000007234	0	0	0
0	0	0	0,000007234	0	0	0
0	0	0	0,000007234	0	0	0
0	0	0	0,000007234	0	0	0
0	0	0	0,000007234	0	0	0
0	0	0	0,000007234	0	0	0
0	0	0	0,000007234	0	0	0
0	0	0	0,000007234	0	0	0
0	0	0	0,000007234	0	0	0
0	0	0	0,000007234	0	0	0
0	0	0	0,000007234	0	0	0
0	0	0	0,000007234	0	0	0
0	0	0	0,000007234	0	0	0
0	0	0	0,000007234	0	0	0
0	0	0	0,000007234	0	0	0
0	0	0	0,000007234	0	0	0
0	0	0	0,000007234	0	0	0
0	0	0	0,000007234	0	0	0
0	0	0	0,000007234	0	0	0
0	0	0	0,000007234	0	0	0
0	0	0	0,000007234	0	0	0
0	0	0	0,000007234	0	0	0
0	0	0	0,000007234	0	0	0
0	0	0	0,000007234	0	0	0
0	0	0	0,000007234	0	0	0
0	0	0	0,000007234	0	0	0
0	0	0	0,000007234	0	0	0
0	0	0	0,000007234	0	0	0
0	0	0	0,000007234	0	0	0
0	0	0	0,000007234	0	0	0
0	0	0	0,000007234	0	0	0
0	0	0	0,000007234	0	0	0
0	0	0	0,000007234	0	0	0
0	0	0	0,000007234	0	0	0
0	0	0	0,000007234	0	0	0
0	0	0	0,000007234	0	0	0
0	0	0	0,000007234	0	0	0
0	0	0	0,000007234	0	0	0
0	0	0	0,000007234	0	0	0
0	0	0	0,000007234	0	0	0
0	0	0	0,000007234	0	0	0
0	0	0	0	0	0	0
0	0	0	0	0	0	0
0	0	0	0	0	0	0
0	0	0	0	0	0	0
0	0	0	0	0	0	0
0	0	0	0	0	0	0
0	0	0	0	0	0	0
0	0	0	0	0	0	0
0	0	0	0	0	0	0

0,00000576	0	0	0	0	0	0
0,00000576	0	0	0	0	0	0
0,00000576	0	0	0	0	0	0
0,00000576	0	0	0	0	0	0
0,00000576	0	0	0	0	0	0
0,00000576	0	0	0	0	0	0
0,00000576	0	0	0	0	0	0
0,00000576	0	0	0	0	0	0
0,00000576	0	0	0	0	0	0
0	0	0	0	0	0	0
0	0	0	0	0	0	0
0	0	0	0	0	0	0
0	0	0	0	0	0	0
0	0	0	0	0	0	0
0	0	0	0	0	0	0
0	0	0	0	0	0	0
0	0	0	0	0	0	0
0	0	0	0	0	0	0
0	0	0	0	0	0	0
0	0	0	0	0	0	0
0	0	0	0	0	0	0
0	0	0	0	0	0	0
0	0	0	0	0	0	0
0	0	0	0	0	0	0

Sample59	sample60	sample62	sample9	Sample3	Sample29	Sample13
0,000104544	0,00171936	0,62355249	0,0009975	0,00057521	0,005905	0,0002382
0,000073689	0,000542592	0,02584857	0	0,00136285	0,0226781	0,0002465
0,000411279	0,000166752	0,00136851	0,000513	0,00096321	0,01483815	0,0003101
0,005109225	0,009146304	0,01405833	0,0283718	0,01652201	0,02877489	0,0078224
0,006577197	0,005215968	0,00485199	0,0183089	0,01916332	0,02893046	0,0114238
0,000030855	0	0,00000957	0	0,00050731	0,00999013	3,98E-05
0,000018513	0,002228256	0,00131109	0,0014963	0,00038315	0,00119762	0,0001034
0,000104544	0,000158976	0,00001914	0,0003016	0,00056454	0,00350336	0,0005245
0,000067518	0,00049248	0,04103616	0,0006033	0,00017654	0,00127434	9,563E-05
0,000294756	0,00025056	0,00022011	0,0020093	0,00060625	0,00606696	0,0005881
0,000037026	0,000517536	0,01236444	0,0003183	0,00025899	0,00098026	4,752E-05
0,000006171	0,000058752	0,00706266	0,0004679	0,00042971	0,00008524	7,722E-06
0,000165891	0,000625536	0,01611588	0,0045481	0,00088076	0,00154284	4,752E-05
0,000540507	0,001393632	0,00614394	0,0037478	0,00170429	0,003595	0,0007793
0	0,000100224	0,00008613	0,0007101	0,00004171	0,00047308	7,96E-05
0,001019304	0,001160352	0,00104313	0,0035958	0,00462108	0,00683838	0,0024485
0,000012342	0,000717984	0,00002871	0,0010878	0,00021243	0,0012786	0,0001669
0,000024684	0,00035856	0,00026796	0,0007553	0,00006693	0,00078208	0,000183
0,000601854	0,001210464	0,0018183	0,0031279	0,00291194	0,00452411	0,0013038
0,000006171	0,00013392	0,00010527	0,0002565	0	7,6716E-05	5,584E-05
0	0,00013392	0,00006699	0,000076	0	0	0
0,000030855	0,000033696	0,0017226	0	0,00016102	0,00074585	1,604E-05
0,000208725	0,004948992	0,00648846	0,005529	0,0011349	0	0,0003101
0,000190212	0,00281232	0,00161733	0,0038831	0,00023862	0,00019392	0
0	0,000141696	0,00676599	0,0003183	0	0	0
0,000442134	0,001377216	0,00163647	0,0014511	0,00100977	0,00319224	0,0011286
0	0,000100224	0,0216282	3,088E-05	0,0000776	0,00025359	7,722E-06
0	0,000033696	0,03173412	0,0001663	0,00001552	0,00029408	6,356E-05
0,000890439	0,000292032	0,00039237	0,001691	0,00074593	0,00461362	0,0009219
0	0,00012528	0,0072732	3,088E-05	0,00001067	0,0002749	0
0	0,00000864	0,00000957	1,425E-05	0	0	0
0,001043988	0,000150336	0	0,000437	0,00194291	0,00362909	0,0008031
0,000042834	0,001452384	0,00098571	0,0041396	0	1,0655E-05	0
0,000165891	0,001017792	0,00159819	0,0036266	0,00169459	0	0,0002228
0,000006171	0,001610496	0,00136851	0,0010284	0,00000485	0,00092912	3,98E-05
0,000024684	0,000025056	0,00067947	0	0	0,00002131	0
0	0,001268352	0,00050721	0,0026125	0,00009312	1,0655E-05	0
0,000110715	0,000208224	0,00080388	0,0007861	0,0005335	0,00148744	0,000493
0	0,000083808	0,01267068	4,513E-05	0,00002619	0,00010229	0
0,000564828	0,00037584	0,00760815	0,0003776	0,00049179	0,00036653	0,0005643
0,00023958	0,000350784	0,00022968	0,000589	0,00065281	0,00168562	0,0004847
0	0,000884736	0,00032538	0,0007244	0,00001067	0	0,000294
0,000632709	0	0	0,0001069	0,00107282	0,00091846	0,0008346
0	0	0,00012441	0,0021161	0,00003104	0,00017261	4,752E-05
0	0	0	0	0	0	0
0,000012342	0,000091584	0,00030624	0,0001069	0,00005141	0,00012786	7,128E-05
0	0,000041472	0,00001914	0,000076	0	0	0
0,000122694	0,00025056	0,00013398	0,000589	0,00010379	1,0655E-05	8,732E-05
0	0	0,00005742	3,088E-05	0	2,9834E-05	0
0,00033759	0,000066528	0,00006699	0,0001069	0,00003104	0,00245278	0,0001432
0,000153549	0,00012528	0,00034452	0,0008764	0,00109804	0,00137023	0,000294
0	0,000141696	0	0,001767	0,00035793	0	0,0001746
0,000178233	0,00049248	0,00089958	0,0017979	0,00037345	0,0007352	0,0002625
0,000110715	0,0007344	0,00101442	0,0017528	0,0004559	0,00055832	0,000183

0	0	0,00018183	1,425E-05	0,00010864	0,0004262	1,604E-05
0	0,000025056	0,00629706	3,088E-05	0	0,00014278	0
0,00007986	0,00048384	0,00022011	0,0003919	0,00007275	0,00010229	0
0,000184404	0,000400896	0,00087087	0,001254	0,0003104	0,00045603	0,000199
0,000128865	0,00023328	0,00031581	0,0010426	0,0004559	0,00092485	0,0002625
0,000006171	0,000100224	0,00111012	0,0020093	0	0,00093338	0
0,001719531	0,000793152	0,00006699	0,0009524	0,00123869	0,00111238	0,0021705
0,000135036	0,00048384	0,00043065	0,0011471	0,00058006	0,00068618	0,0002305
0,000006171	0,000959904	0,00021054	1,425E-05	0,00027936	0,00089928	0,0002067
0,000030855	0,000025056	0,00032538	9,025E-05	0	0,00038997	0
0,000957957	0,000768096	0,0000957	0,0016174	0,00073526	0,0008524	0,0012005
0	0	0,00109098	0,000076	0,00002619	0,00014278	0
0	0,000175392	0,00046893	0,0010878	0,00039867	0,0010655	0,0001354
0,000006171	0,000025056	0,00007656	0,000589	0,00004171	9,5895E-05	3,208E-05
0,000030855	0,00012528	0,00030624	0,0019024	0,00028518	0,00021736	0,0001354
0,000128865	0,000158976	0,00337821	3,088E-05	0,00019206	0,00017687	2,376E-05
0,000012342	0,0003672	0,00022968	0,0031113	0,00007275	0	0
0	0	0,00000957	0	0	3,6227E-05	0
0	0,000100224	0,00025839	0,0041539	0,00000485	0	0
0,000042834	0,000066528	0,0001914	0,0003016	0,0004559	0,00069044	0,0001592
0,000006171	0,000191808	0,00030624	0,0011186	0,00004171	0,00010229	0,0001354
0,000012342	0,000467424	0,00022968	0,0009524	0,00001067	0	0
0	0,000025056	0,00636405	4,513E-05	0	4,0489E-05	0
0,000012342	0,000451008	0,00024882	0,002356	0,00002037	0	0
0,000098373	0,000025056	0,00003828	9,025E-05	0,00006693	0,00031965	0,0002144
0,000006171	0,00000864	0	0,0001069	0,00001067	0,00010229	5,584E-05
0	0	0,00638319	0	0	1,4917E-05	0
0	0,00000864	0,00005742	0	0	5,5406E-05	0
0,000030855	0,000325728	0,0003828	0,0009524	0	4,262E-06	0
0,000128865	0,00023328	0,0001914	0,0002423	0,00022795	0,00035588	7,96E-05
0	0	0	0	0	0	0
0	0	0	0	0	0	0
0,000042834	0,000433728	0,00025839	0,0012849	0,00002037	0	0
0,000196383	0	0,00000957	0,000076	0,00001552	0,00002131	0
0,000435963	0,000016416	0	0	0,00052283	0,00067979	0,000493
0,000037026	0,000433728	0,00068904	0,0008004	0,00019206	0,00086732	6,356E-05
0	0,000951264	0,00016269	0,0024629	0,00000485	0	0
0	0	0,00005742	0	0,00005141	0,0002749	0
0	0,000216864	0,00001914	3,088E-05	0	0	0
0,000006171	0	0,0001914	0,0001663	0	0	0
0,000037026	0,000066528	0,00022011	0,002584	0,00006693	0,00011081	3,208E-05
0	0,000033696	0,00001914	3,088E-05	0	2,9834E-05	3,98E-05
0,000006171	0,00000864	0,00000957	0	0	0	6,356E-05
0,000006171	0	0,00023925	1,425E-05	0,00002619	0,00012147	0
0	0	0	0	0,00002037	1,0655E-05	6,356E-05
0	0	0,00029667	0	0	0	0,0010811
0,000018513	0,000526176	0,00008613	0,0014203	0,00002037	0	0
0	0,000016416	0,00103356	0	0,00001552	5,1144E-05	0
0	0,000433728	0,0016269	0,0022349	0,00003589	0	0
0	0,000158976	0	0,0019641	0,00015035	0	0
0,000405471	0,000292032	0	0,0008621	0,0034241	0,00071175	0,0013276
0,001001154	0,00061776	0,00001914	0,0005748	0,00125906	0,00088437	0,0009142
0	0,00000864	0	0,0012849	0	4,262E-06	0
0	0	0	0	0,00002619	4,262E-06	7,722E-06
0	0,000417312	0,00007656	0,0045766	0	6,6061E-05	0

0,000024684	0,00000864	0,00013398	1,425E-05	0,00001067	4,262E-06	3,98E-05
0	0	0	0	0	0	0
0	0	0,00013398	0	0	2,5572E-05	2,376E-05
0,000030855	0,000058752	0,00005742	0,0002423	0,00044523	0,00096961	0,0001111
0,000196383	0,000208224	0,00036366	0,0003183	0,00012416	0,00025359	9,563E-05
0	0,000058752	0,00012441	0	0,00016102	0,00063504	0
0	0	0,00125367	1,425E-05	0	5,1144E-05	0
0,000024684	0,000317088	0,00006699	0,0011186	0,00002037	0	3,98E-05
0	0,000166752	0,00008613	0,0026885	0	4,262E-06	0
0	0,001035072	0,00003828	0,0001805	0,00002037	0,0010655	0
0	0	0	0	0,00010864	0,00029408	0
0	0	0	0	0,00001067	0	0
0,000263901	0,000025056	0,0000957	0,0002256	0,0000776	0,00016835	1,604E-05
0,000067518	0,00012528	0,00017226	0,0003325	0,00010379	0,00039637	0,0001194
0	0	0	0	0	0	0
0,000012342	0,000025056	0	1,425E-05	0,00003104	4,0489E-05	3,208E-05
0,000024684	0,00000864	0,00000957	4,513E-05	0,00000485	0	0
0,000030855	0,000083808	0,00020097	0,0005296	0,0001552	0,00044112	0,0001271
0,00015972	0	0	0	0	0	0
0,000098373	0,000208224	0,00022011	0,0005581	0,0001455	0,00023867	7,96E-05
0	0	0	0	0	0	0
0	0,00049248	0,00005742	0,0003468	0	0	7,128E-05
0	0	0	0	0	0	0
0,000012342	0,00000864	0,00003828	3,088E-05	0,00001067	2,9834E-05	7,722E-06
0,000012342	0,000025056	0,00010527	4,513E-05	0,00002619	1,4917E-05	0
0	0,00000864	0	0	0	0	0
0	0	0	0	0	0	0
0	0,000058752	0,00007656	3,088E-05	0,00001067	4,262E-06	0
0,000006171	0,000225504	0,00007656	4,513E-05	0,00000485	1,0655E-05	7,722E-06
0	0,000308448	0	0	0	0	0,0005803
0	0	0	0	0	0	0
0	0,00023328	0,00002871	0,0001354	0	0	0
0	0,000150336	0,00033495	0,0001354	0	0	0
0,000086031	0,000283392	0,00010527	0,0002256	0,00017072	0,00045177	7,128E-05
0	0	0	0,0009809	0	0	0
0,000012342	0,000108864	0,00013398	0,0001971	0,00003589	0,00020245	1,604E-05
0,000012342	0,000458784	0,00091872	0,0003183	0	0	2,376E-05
0	0	0	0	0	0	0
0,000018513	0,000175392	0,00030624	0,0003183	0,00013968	0,00020884	3,208E-05
0	0,000041472	0,00030624	0,000076	0	0	0
0	0,000016416	0	0	0	0	0
0	0,000025056	0,00002871	0,0009975	0,00048694	0	0
0	0,000041472	0,0014355	9,025E-05	0	0,00002131	0
0	0,000100224	0,00040194	5,938E-05	0,00016587	6,1799E-05	0,0001669
0	0,000083808	0,00015312	0,0001805	0,00001552	0	0
0,000049005	0,00013392	0,00099528	0,000437	0,00008827	0,00021736	1,604E-05
0	0	0	0	0	0	0
0	0,000100224	0,00000957	0,0011186	0	0	0
0	0,000066528	0,0000957	0,000152	0,00003589	0,0002131	0
0,000018513	0,000033696	0,00023925	0,000152	0	7,0323E-05	0
0,000018513	0,000083808	0,00005742	0,0001805	0,00004171	0,00024293	0
0	0	0,00003828	0	0	0	0,0002703
0	0,000016416	0,00000957	0	0,00197395	2,5572E-05	0
0,000055176	0,000058752	0,00020097	0,0003183	0,00019206	0,00008524	6,356E-05
0	0,000659232	0,00006699	0,0001211	0	0	0

0	0,000075168	0,00060291	5,938E-05	0,00001067	6,1799E-05	1,604E-05
0	0,000050112	0,00026796	0,0004988	0,00004171	0,00017687	0,0001111
0	0	0,00006699	0	0,00002037	2,5572E-05	0
0	0,000025056	0,00003828	0	0	0	0
0	0,000150336	0,00006699	0,0002708	0,00026966	0,00021736	0,0001111
0,000037026	0,000016416	0,00010527	1,425E-05	0,00000485	6,6061E-05	0,0003659
0,000214896	0,000058752	0,00003828	4,513E-05	0,00001552	0	0
0	0,000016416	0	3,088E-05	0	0	0
0,000055176	0,000016416	0,00015312	0,0002874	0,00020758	0,0002131	7,128E-05
0,000030855	0,000150336	0,00000957	0,0003634	0	0	0
0	0	0,00002871	0	0	0	0
0,000018513	0,000091584	0,00014355	0,0001354	0,00003589	9,1633E-05	0
0	0	0	0	0	0	0
0,000012342	0,000083808	0,00006699	0,0024178	0,00006693	0,00018753	8,732E-05
0	0,000041472	0,00005742	3,088E-05	0,00008827	0,0001513	9,563E-05
0,000018513	0,000091584	0,00032538	0,0003468	0,00009312	0,00012147	7,128E-05
0	0	0	0	0	0	0,0003813
0,000012342	0,000025056	0	0	0	0	0
0	0	0	0	0	0	0
0	0,00000864	0	0	0	0	0
0	0	0,00000957	0	0	0	3,208E-05
0	0,000108864	0,00003828	0,0002423	0,00020758	0,00018753	0
0	0,00013392	0,00005742	0,0001069	0	0	5,584E-05
0	0,000108864	0,00046893	0,0005296	0	0	0
0,000006171	0,000091584	0,00026796	0,0005439	0,00002619	6,6061E-05	3,208E-05
0	0	0	0	0	0	0
0,000006171	0,000066528	0,00014355	0,0001069	0,0000776	0,00012786	2,376E-05
0	0,000050112	0,00008613	0,000076	0,00008827	0,00026424	5,584E-05
0,000006171	0	0,00000957	0	0	0	0
0	0,000016416	0,00000957	1,425E-05	0,00000485	1,4917E-05	0
0	0	0	0	0	0	0,0002305
0,000190212	0,000166752	0,00007656	0,0001211	0,00010864	0,00016835	0,0001509
0	0,000058752	0	3,088E-05	0,00001067	0	0
0	0	0	0	0	0,00010229	0
0	0,00000864	0	1,425E-05	0	0	0
0	0,000083808	0,00005742	0,0004085	0	0	0
0	0	0	0	0	0	0
0	0	0	0	0	0	0
0,000037026	0	0,00005742	0	0,00003589	0,00011721	0,0001111
0	0,000050112	0,00001914	0,0001663	0	0,00023867	0
0,000006171	0,000016416	0	1,425E-05	0	4,262E-06	0
0,000073689	0,000141696	0,00020097	0,0003919	0,00005141	0,00011721	0
0	0	0,00001914	0,000152	0	0,00013638	0
0,00007986	0,000016416	0,00002871	3,088E-05	0,00013483	0,00018753	0
0	0	0,00000957	0	0,00001067	1,4917E-05	0
0,000030855	0,000058752	0,00018183	0,0002256	0,00005141	6,6061E-05	0,0004449
0	0,000050112	0,00001914	0	0	0,0002749	0
0	0	0,00045936	0	0	4,0489E-05	0
0,000024684	0	0,00001914	3,088E-05	0,00001067	4,262E-06	0
0	0,000150336	0,0002871	0,0005439	0,00004171	0	0
0,000024684	0,000083808	0,00016269	0,0001663	0,00008245	0,00013638	6,356E-05
0	0,000200448	0,00024882	0,0004679	0	4,262E-06	0
0	0	0	0	0	0	0
0,000202554	0	0,00004785	1,425E-05	0	0	0
0	0	0	0,000152	0,00021243	0,00014278	0

0	0,000075168	0,00005742	1,425E-05	0,00003589	5,5406E-05	3,98E-05
0	0,000325728	0,00013398	0,000513	0,00000485	2,5572E-05	0
0,000012342	0	0	3,088E-05	0,00003104	3,6227E-05	0
0	0	0	0	0,00000485	0	0
0	0,00000864	0,00070818	0,0037763	0,00000485	0	0
0	0,00000864	0	0	0,00000485	0	0
0	0	0	9,025E-05	0	0,00018327	0
0	0,000091584	0,00007656	0,0002708	0,00002037	0,00010229	2,376E-05
0,000042834	0,000025056	0	0,0001069	0	0	0
0	0	0	0	0	0	0
0	0,000041472	0,0000957	0,0003183	0	0	0
0	0	0,00005742	1,425E-05	0	0	0
0	0	0,00000957	5,938E-05	0	4,262E-06	7,722E-06
0	0	0	0	0	0	0
0	0	0	0	0	0	0
0	0	0	0	0,00000485	0	0
0	0,00000864	0,00000957	0	0	0	1,604E-05
0	0	0	0	0,00003589	0,00016196	0
0	0	0,0002871	0	0	0	0
0	0,00000864	0	0	0	0,00075224	0
0	0	0,00000957	0	0	0	0,0004057
0	0	0,00014355	9,025E-05	0,00000485	6,1799E-05	1,604E-05
0	0,00000864	0	4,513E-05	0,00003104	5,1144E-05	2,376E-05
0,000006171	0,000050112	0,0001914	0,0002114	0	4,4751E-05	1,604E-05
0,000037026	0,000033696	0,00011484	0,0001971	0,00009797	0,00008524	2,376E-05
0	0	0	0	0	0	0
0,000012342	0,000041472	0,00004785	0	0	0,00002131	0
0	0	0,00194271	0	0	0	0
0	0	0	0	0,00000485	0	0
0	0,000058752	0,00007656	5,938E-05	0,00005723	3,6227E-05	7,722E-06
0	0	0,00000957	0	0	0	0
0	0	0,00127281	0	0	2,5572E-05	0
0	0	0	0	0	0	0
0	0,000058752	0,00015312	0,0002565	0,00008827	0,00014704	0
0	0,000016416	0	1,425E-05	0,00001552	2,9834E-05	0
0	0,000025056	0,00039237	0,000076	0,00000485	6,1799E-05	7,722E-06
0,000092202	0,000216864	0,00005742	9,025E-05	0,0003007	0,00017261	0,0005405
0	0,00000864	0,00015312	1,425E-05	0,00002619	0,00011081	0
0,000300927	0,000141696	0	0,0001971	0,00024832	0,00018753	0,0003338
0,000006171	0	0,00004785	0	0	0	0
0	0	0,00006699	0	0,00001067	4,0489E-05	0
0	0	0	0	0,00123287	0,00002131	0
0	0,000050112	0,00106227	1,425E-05	0,00004171	4,0489E-05	1,604E-05
0	0,000675648	0,00048807	0,0001211	0,00001552	4,262E-06	7,722E-06
0	0	0	0	0	4,262E-06	0
0	0,000417312	0,00007656	0,0004845	0	0	0
0,000024684	0,000033696	0,00018183	9,025E-05	0,00003589	4,4751E-05	7,722E-06
0	0,00000864	0	0	0	4,262E-06	0
0	0	0	0	0,00042001	0,00013638	0
0	0	0,00001914	0	0	0	0
0	0	0	0	0,00000485	0	0
0	0,000016416	0	0,0003776	0	0	0
0	0	0	0	0	0	0
0	0,000317088	0,00004785	0,001254	0,00001067	1,0655E-05	7,722E-06
0	0	0	0	0	1,4917E-05	0

0	0,000075168	0,00003828	0,0001069	0,00004171	0	9,563E-05
0,000012342	0,000041472	0,00016269	0,0001069	0,00004171	0,00011081	1,604E-05
0	0	0	0	0	0,00043046	0
0,000098373	0,000016416	0	3,088E-05	0	0	0
0	0	0,00003828	0,0001354	0	6,1799E-05	0
0	0	0	0	0	0	0
0	0	0,00000957	0	0	0	0
0	0,000033696	0	0,0004085	0	0	0
0	0	0	5,938E-05	0	0	0
0,000024684	0,000066528	0,00015312	0,0001069	0,00002037	6,1799E-05	3,208E-05
0	0	0,00001914	0	0	2,9834E-05	0
0	0,000025056	0,00001914	0,0002256	0	0	0
0	0	0	0,0037478	0	0	0
0	0	0	0	0	0	0
0	0,000016416	0,00042108	3,088E-05	0	0	0
0	0,00000864	0,00003828	0	0,00001552	0	0
0	0,000016416	0,00073689	0	0	0	0
0	0,00012528	0,00001914	9,025E-05	0	4,262E-06	0
0	0	0	0	0	1,4917E-05	0
0	0,00000864	0	0,0004845	0	0	0
0,000006171	0,00011664	0,00011484	0,0001971	0,00003589	6,1799E-05	1,604E-05
0,000024684	0,000016416	0,00002871	0,0001069	0	7,0323E-05	3,208E-05
0,000073689	0	0	0	0	0	0
0	0	0,00020097	0	0	0	0
0	0,000016416	0,00006699	0	0,00000485	0	0
0	0	0	0	0	0	0
0	0,000058752	0,00006699	0,0003016	0	0	0
0	0,000266976	0	1,425E-05	0	0	0
0,000006171	0,000050112	0,00008613	9,025E-05	0,00003104	4,0489E-05	3,98E-05
0	0,000050112	0,00003828	0,0011471	0	0	0
0	0	0	0	0	0	0
0	0	0	0	0	0,00002131	0
0	0	0,00011484	0,0009809	0	0	0
0	0,000208224	0,00000957	1,425E-05	0	2,5572E-05	0
0,000049005	0,000041472	0,00001914	0	0	8,0978E-05	0
0,000037026	0	0	3,088E-05	0,00003589	4,4751E-05	7,128E-05
0	0,000050112	0,00002871	0	0	0	0
0	0	0,00053592	0	0	0	0
0,000018513	0,000075168	0,0000957	5,938E-05	0,00005723	1,4917E-05	2,376E-05
0,000006171	0	0	0	0,00001067	4,262E-06	5,584E-05
0	0,000041472	0,00006699	0,0001069	0,00002619	5,1144E-05	1,604E-05
0	0	0,00000957	0	0	4,262E-06	0
0	0	0	0	0	0	0
0	0	0	0	0	0	0
0	0	0	0	0	0	0
0	0	0,00004785	0	0	0	0
0	0,00000864	0	0	0	0	0
0	0,000041472	0,00014355	0,0001069	0,00000485	5,5406E-05	1,604E-05
0	0	0,00016269	0	0	0	0
0	0,00000864	0	4,513E-05	0	1,0655E-05	0
0	0,000016416	0,00000957	4,513E-05	0,00003589	0,00002131	3,208E-05
0	0	0	0	0	0	0
0	0	0	0,0027645	0	0	0
0	0	0,00000957	0	0	0	0
0	0	0,00023925	0	0	0	0

0	0	0	0	0	0	0
0	0	0	0	0	0	0
0	0	0	0	0	4,262E-06	0
0	0	0	0	0	0	0
0	0	0	0	0	1,0655E-05	0
0,000037026	0,000033696	0,00003828	0	0,0001455	3,6227E-05	2,376E-05
0	0	0	0	0	0	0
0	0,000317088	0,00016269	0,0002423	0	0	0
0,000018513	0,000041472	0,0000957	0,0001211	0,00000485	6,1799E-05	0
0	0,000016416	0,00001914	0,0003919	0	0	0
0,000049005	0,000016416	0	0	0,00006693	0,00014704	0
0	0	0	0	0	0	0
0	0,00000864	0,00001914	3,088E-05	0	1,0655E-05	0
0,000006171	0,000016416	0,00002871	4,513E-05	0,00003104	4,0489E-05	2,376E-05
0	0,00000864	0,00000957	0	0	0	0
0	0	0	0	0	0	0
0	0,00000864	0,00054549	0	0	1,4917E-05	0
0	0	0	0	0,00034241	0	0
0	0	0	0	0	0	0
0	0	0,00000957	0	0	0	0
0	0	0	1,425E-05	0,00000485	4,262E-06	0
0	0	0	0	0	0,00002131	0
0,000024684	0,00000864	0,00001914	0	0,0000776	0	1,604E-05
0	0	0	0	0	0	0
0	0	0	0	0	0	0
0,000098373	0,000100224	0	0,0001211	0,00023862	0,00010229	0,000278
0	0	0	5,938E-05	0	0,00022802	0
0	0,000025056	0,00046893	4,513E-05	0,00000485	1,0655E-05	0
0	0,00195264	0	0	0	0	0
0	0	0	9,025E-05	0	0	0
0	0,000016416	0,00000957	3,088E-05	0,00002619	2,5572E-05	7,722E-06
0,000012342	0	0,00018183	4,513E-05	0,00001552	7,0323E-05	0
0	0	0	1,425E-05	0	0	0
0,000018513	0,000041472	0,0000957	0,0002708	0	0	0
0	0	0	0,000076	0	0	9,563E-05
0	0	0	0	0	0,00016196	0
0	0	0,00003828	0	0	1,4917E-05	0
0	0	0	0,0021898	0	0	0
0	0	0,00000957	0	0,00000485	1,4917E-05	0
0	0,00000864	0,00031581	9,025E-05	0,00002037	2,5572E-05	7,722E-06
0	0	0,00003828	0	0	0	0
0	0,00000864	0,00002871	0	0	0	0
0	0	0	0	0	0	0
0	0,000041472	0	0	0	0,00012786	0
0,000006171	0,000100224	0,00001914	4,513E-05	0	0	0
0	0	0	3,088E-05	0	0	0
0	0	0,0001914	0	0	1,0655E-05	0
0,000263901	0,000025056	0	0,0001211	0	0	0
0	0	0	0	0	0	0
0	0	0	0	0	0	7,722E-06
0,000006171	0	0	0	0	0	7,722E-06
0	0	0,00001914	0	0	0	7,722E-06
0	0,000066528	0,00012441	5,938E-05	0,00002037	1,0655E-05	0
0,000006171	0	0,00001914	0	0,00000485	4,262E-06	0
0	0	0,00002871	0	0,00002037	1,0655E-05	0

0	0,000041472	0,0000957	4,513E-05	0	0	0
0	0	0	0	0	0	0
0	0,000041472	0	0,0008146	0	0	0,0001669
0	0	0,00000957	0	0	1,0655E-05	0
0	0	0,0001914	0	0	1,0655E-05	0
0	0	0	0	0	0	0
0	0	0	0	0	0	3,208E-05
0	0,00000864	0,00048807	0,0014796	0	0	0
0,000006171	0,000066528	0,00002871	1,425E-05	0	5,5406E-05	7,722E-06
0	0	0	0	0	0	0
0	0	0	0,000076	0	0	0
0	0	0,00000957	1,425E-05	0,00000485	0	0
0,000049005	0	0,00001914	0	0,00002619	6,1799E-05	0
0	0	0,00023925	0	0	0,00002131	0
0	0,000091584	0,00002871	0,0001663	0	0	0
0	0	0,00021054	0,000076	0	0	0
0	0	0,00003828	1,425E-05	0,00000485	1,0655E-05	7,722E-06
0	0,00050112	0	0	0	0	0
0	0,000016416	0,00011484	0	0	0	0
0	0,000033696	0,00001914	4,513E-05	0	0,00002131	0
0	0,00000864	0	1,425E-05	0	1,0655E-05	0
0	0,000066528	0,00001914	4,513E-05	0,00003589	7,6716E-05	8,732E-05
0	0	0,00005742	1,425E-05	0	1,4917E-05	0
0,000037026	0,000058752	0	0,000076	0,00001552	0,00028342	0,0003261
0	0,000141696	0	0,0004536	0	0	0
0	0	0,00000957	0	0	0	0
0	0	0	0,0003183	0	0,00145121	0
0,000073689	0,000033696	0	0,0001211	0,00010379	4,0489E-05	1,604E-05
0	0	0	0	0	0	0
0	0,00000864	0	0	0	0	0
0,000012342	0	0,00035409	1,425E-05	0,00001067	0	2,376E-05
0,000018513	0,00000864	0,00000957	0,0012231	0	0	0
0	0	0	0,0003016	0	0	0
0	0	0,00002871	1,425E-05	0,00004656	2,9834E-05	0
0,000086031	0,000058752	0	0,0001211	0,00029488	0	0,0001271
0,000006171	0	0,00001914	4,513E-05	0,00001067	2,5572E-05	7,722E-06
0	0	0	0	0	0	0
0	0,000016416	0,00000957	0,0001354	0	0	0
0	0	0,00023925	0	0	0	0
0	0	0,00007656	0	0	0	0
0	0	0	0	0	0,00020245	0
0	0,00000864	0,00003828	0	0,00009312	1,0655E-05	0
0,00007986	0	0	0	0,00005141	4,4751E-05	0
0	0	0	0	0	0	0
0,000030855	0,000025056	0,00001914	0	0	0,00002131	3,98E-05
0	0	0	0	0	0	0
0	0,000016416	0	0,0007553	0	0	0
0	0,000041472	0	0	0	0	0
0	0,00000864	0,00000957	1,425E-05	0	1,0655E-05	0
0,000049005	0	0	0	0,00033659	6,6061E-05	0,0001111
0	0	0,00000957	0	0	0	0
0,000067518	0,00000864	0,00004785	0	0,00001067	0	0
0	0,00000864	0,00060291	1,425E-05	0	4,262E-06	0
0	0	0	0	0	0	0
0	0,000025056	0,00002871	0,0001354	0	0	0

0	0	0	0	0	0	0
0	0	0	0	0	0	0
0	0	0	0	0	5,5406E-05	0
0	0,000058752	0,00003828	0	0	4,262E-06	0
0	0	0,00003828	5,938E-05	0	1,4917E-05	0
0,000006171	0	0,00003828	3,088E-05	0,00001067	4,0489E-05	0
0	0	0	0	0	4,262E-06	0
0	0	0	0	0	0	0
0	0	0	0	0	1,0655E-05	0
0	0	0	0	0	0	0
0	0,000158976	0	0	0	0	0
0	0	0	0	0	0	0
0	0	0	0	0	0	0
0	0	0,00000957	0	0	9,5895E-05	0
0	0,00000864	0,00000957	3,088E-05	0	4,262E-06	0
0	0,00000864	0,00001914	0	0,00000485	0	0
0	0,000025056	0,00001914	4,513E-05	0,00002037	2,5572E-05	1,604E-05
0	0,00000864	0	0	0,00000485	2,9834E-05	0
0	0	0	0	0	0	0
0	0	0	0	0	0	0
0	0	0,00005742	0	0	0	0
0	0	0,00002871	4,513E-05	0,00001067	2,9834E-05	7,722E-06
0	0	0,00029667	0,0004228	0	0	0
0	0	0,00001914	0	0,00001552	1,0655E-05	7,722E-06
0	0	0	0	0	0	0
0	0,000091584	0,00000957	1,425E-05	0	0	0
0	0	0	0	0	0	0
0	0	0	0	0	0	0
0	0,000025056	0	4,513E-05	0	0	0
0	0,000025056	0,0001914	0	0	5,5406E-05	0
0	0,00000864	0,00001914	5,938E-05	0,00001067	1,4917E-05	7,722E-06
0	0,00035856	0,00058377	0	0	0	0,000294
0	0	0,00000957	0	0	4,262E-06	0
0	0,00000864	0,00001914	0	0	0	0
0,000116523	0,000033696	0	0	0,00005723	9,5895E-05	3,98E-05
0	0	0	0	0	0	0
0	0	0	0,0012683	0	0	0
0	0	0	3,088E-05	0,00005141	4,4751E-05	0
0	0,00000864	0,00050721	0	0	0	0
0	0,000025056	0,00003828	1,425E-05	0	6,6061E-05	0
0	0,000183168	0	0	0,00063729	4,262E-06	0
0	0,000016416	0,00013398	4,513E-05	0	4,262E-06	7,722E-06
0	0	0,00000957	0	0	0	0
0	0	0,00001914	0	0,00000485	0	0
0	0,00000864	0,00004785	0	0	0	0
0	0	0,00053592	1,425E-05	0	0	0
0	0	0	0	0,00008245	0	0
0	0	0	4,513E-05	0	0	0
0	0	0,00000957	0	0	0	2,376E-05
0	0,000066528	0	9,025E-05	0	0	0
0	0	0	0	0	0	0
0	0	0	0,000152	0	4,262E-06	0
0	0	0	0	0	0	0
0	0,000016416	0	0	0	0	0,0001034
0	0	0,00032538	0	0	1,0655E-05	0

0	0	0	0	0	0	7,722E-06
0	0	0	0	0	0	0
0	0,000033696	0,00001914	1,425E-05	0,00002619	3,6227E-05	7,722E-06
0	0	0	0	0	0	0
0	0	0	0	0	0	0
0	0,000033696	0	0,0001069	0	0	0
0	0,000041472	0,00000957	0	0,00001552	0	0
0	0,000033696	0,00002871	4,513E-05	0	0	0
0	0	0,00024882	0	0	0	0
0,000012342	0	0,00001914	0	0	0	0
0	0	0	0	0	0	0
0	0	0	0	0,00000485	0	0
0	0	0	0,0002708	0,00001067	0,00057963	0
0,000006171	0,000041472	0,00000957	0,0001069	0,00000485	3,6227E-05	0
0	0	0,00000957	0	0	0	0
0	0	0,00001914	0	0	0	0
0	0,000025056	0,00012441	0	0	0	0
0	0	0,00041151	0	0	4,262E-06	0
0	0,000016416	0	0	0	0	0
0	0,000025056	0,00001914	4,513E-05	0	2,5572E-05	0
0	0,00000864	0	0,000076	0,00005141	3,6227E-05	0,0001034
0	0	0	0	0	0	0
0	0	0,00002871	0,0001069	0	0	0
0	0	0	0	0	0	0
0	0,000025056	0	0	0,00001067	1,0655E-05	3,208E-05
0	0	0	0	0	0	0
0	0	0,00000957	0	0	1,0655E-05	0
0	0,00000864	0,00003828	1,425E-05	0	0	0
0	0	0	0	0	0	0
0	0	0	0	0	0	0
0	0,00024192	0	0	0	0	0
0	0,000050112	0,00005742	0,0002114	0	0	0
0	0	0,00000957	0	0	0	0
0	0	0,00001914	0	0	0	0
0	0,00000864	0	0,0001211	0	0	0
0	0,00000864	0,00006699	0	0	0	0
0	0	0,00000957	0	0	4,262E-06	0,000278
0	0	0,00001914	0	0	0	0
0	0,000058752	0,00000957	0,0002256	0	0	0
0	0	0,00024882	0	0	4,262E-06	0
0,000030855	0,000041472	0,00007656	0	0,00006693	1,0655E-05	7,96E-05
0	0	0,00015312	0	0,00001552	4,262E-06	7,722E-06
0	0,00000864	0,00001914	0	0,00000485	0	0,0001034
0	0	0	0	0	0	0
0	0	0	0	0	0	0
0	0	0,00001914	0	0	0,00002131	7,722E-06
0	0	0	0	0	0	0
0	0,000016416	0,00003828	1,425E-05	0	1,0655E-05	7,722E-06
0	0	0	0	0	0	0
0	0	0	0,0004988	0	0	0
0	0,000025056	0,00001914	3,088E-05	0	1,4917E-05	0
0	0	0,00011484	0	0	0	0
0,000006171	0	0,00000957	0	0,00026384	0,00014704	7,722E-06
0	0	0,00000957	0	0	0	0
0	0	0	0	0	0	0

0	0,00000864	0,00000957	1,425E-05	0	0	0
0	0	0	0	0	0	0
0	0	0,00001914	0	0	0	0
0	0	0,0002871	1,425E-05	0	1,0655E-05	0
0,000018513	0,000050112	0	0,0001069	0,0000776	4,262E-06	3,98E-05
0	0	0,00000957	0	0	4,262E-06	0
0	0,00000864	0,00010527	0	0	4,262E-06	0
0,000012342	0	0,00002871	0,0001211	0	0	0
0	0	0,00002871	0	0	0	0
0	0	0	0	0	0	0
0	0	0	0	0	0	0
0,000024684	0,000025056	0	0	0	0	5,584E-05
0	0,000016416	0,00000957	1,425E-05	0	4,262E-06	7,722E-06
0	0,000058752	0	0	0	0	0
0	0,00000864	0,00003828	1,425E-05	0,00000485	0	7,722E-06
0	0	0	3,088E-05	0	0	0
0	0	0	0	0	0	0
0	0	0	0	0	4,0489E-05	0
0,000006171	0	0	1,425E-05	0,00000485	0	0
0	0,000075168	0,00002871	0	0	0	0
0	0	0,00000957	3,088E-05	0,00002619	0,00002131	0
0	0	0	1,425E-05	0	0	0
0,000006171	0	0,00010527	0	0	0	0
0	0,000016416	0,00003828	0,000076	0	0	0
0	0	0,00001914	0	0	0	0
0	0	0	0	0	0	0
0,000030855	0	0	0	0	0	0
0	0	0	0	0	0	0
0	0	0,0000957	0	0	0	0
0	0	0	0	0	0	0
0	0	0	1,425E-05	0	4,262E-06	0
0,000024684	0	0	0	0,00002037	0,00002131	0
0,000006171	0,000016416	0,00002871	0	0,00000485	5,1144E-05	1,604E-05
0	0	0	0	0	0	0
0	0,00000864	0	0	0	0	0
0	0,00000864	0,00022011	0	0,00000485	4,262E-06	0
0	0	0	0	0	0	0
0	0,000025056	0	0	0	0	3,98E-05
0,000018513	0	0	0	0	0	4,752E-05
0	0	0	0	0	0,0002749	0
0	0	0,00005742	0	0,00001067	1,0655E-05	0
0	0	0	0	0	0	0
0	0	0,00001914	0	0,00004171	0	0
0,000018513	0	0	0	0,00010379	1,4917E-05	4,752E-05
0	0	0	0	0	0	0
0	0	0	1,425E-05	0,00000485	4,262E-06	0
0	0	0	0	0,00001067	0	0
0	0	0,00004785	0	0	0	0
0	0	0	0	0	2,5572E-05	0
0,000006171	0,000033696	0,00000957	1,425E-05	0,00000485	4,262E-06	0
0	0	0	0	0	0	0
0	0	0,00000957	0,0002708	0,00004656	0	0
0	0,00000864	0	0,000076	0	0	0
0	0,000033696	0,00004785	1,425E-05	0	0	0
0	0	0	0	0	1,4917E-05	0

0	0	0	0,0006341	0	0	0
0	0	0	0	0	4,262E-06	0
0	0	0,00000957	1,425E-05	0,00000485	2,9834E-05	7,722E-06
0	0	0	1,425E-05	0,00000485	1,0655E-05	0
0,000006171	0	0,00005742	1,425E-05	0,00000485	4,262E-06	0
0	0	0	0	0	0	0
0	0	0	0	0	0	0
0	0	0	0	0	0	0
0	0	0	0	0	0	0
0	0	0	0	0	0	1,604E-05
0	0	0	0	0	4,262E-06	0
0	0	0,00000957	0	0	0	0
0	0	0	0	0	0	0
0	0	0	0	0	0	0
0	0,000016416	0	0	0,00001067	1,0655E-05	0
0	0	0	0	0	0	6,356E-05
0	0	0	0	0	0	0
0	0	0	0	0	0	0
0	0,000016416	0	0	0	0	0
0	0	0,00000957	0	0	3,6227E-05	0
0	0,000016416	0	3,088E-05	0,00001552	0	0
0	0	0,00001914	0	0	1,0655E-05	0
0	0	0	0	0	0	0
0,000012342	0	0	0	0,00001552	0	3,98E-05
0	0	0	0	0	0	0
0	0	0,00000957	0	0	0	0
0	0	0	0	0,00002037	0	0
0	0	0	0	0	0	0
0,000018513	0,00000864	0,00006699	0	0	1,0655E-05	0
0	0	0	0	0	0	0
0	0	0	0	0	0	0
0	0,00011664	0,00002871	0	0	1,0655E-05	0
0	0	0	0	0	0	0
0	0,00048384	0	0	0	0	0
0	0,000016416	0,00000957	0	0	0	0
0	0	0	0	0	0	0
0	0	0	0	0	0	0
0	0	0,00001914	0	0	0	0
0	0	0,00000957	0	0	0	0
0	0	0	0	0	0	0
0	0,00000864	0,00002871	0	0,00001067	4,262E-06	1,604E-05
0	0	0	0	0,00002619	0	0
0,000110715	0	0	0	0	0	7,722E-06
0	0	0	0	0	0	0
0	0	0	0	0	0	0
0	0	0	0	0	0,00050718	0
0	0	0	0	0	0	0
0	0	0	0	0	0	0
0	0	0	0	0	0	0
0	0	0	0	0	4,262E-06	0
0	0	0,00000957	0	0	0	0
0	0	0	0	0	0	0
0	0	0	0	0	0	0
0	0	0	0	0	4,262E-06	0
0	0	0	0	0	0,00041555	0

0	0	0,00000957	0	0	0	0
0	0,00000864	0,00000957	0,0001663	0	0	0
0	0	0	3,088E-05	0	2,9834E-05	0
0	0	0,00000957	1,425E-05	0	0	7,722E-06
0	0	0	0	0	0	0
0	0	0,00000957	0	0,00001067	4,262E-06	0
0	0	0	0	0	0	0
0	0	0	0	0	0	0
0	0	0,00003828	1,425E-05	0	0	0
0	0	0	0	0	0	0
0	0	0	3,088E-05	0	0	0
0	0	0	0	0	0	0
0	0	0	0	0	4,262E-06	0
0	0	0	0	0	0	0
0	0,0003672	0	5,938E-05	0	0	0
0	0	0	0	0,00003104	1,4917E-05	3,98E-05
0	0	0	0	0	0	0
0	0	0	0	0	0	0
0	0	0,00008613	0	0,00000485	4,262E-06	0
0,000018513	0	0,00000957	3,088E-05	0	4,0489E-05	0
0,000006171	0	0	0	0,00000485	0	0
0	0	0	0	0	0	0
0	0	0	0	0	0	0
0	0,000016416	0,00000957	0	0	0,00002131	0
0	0	0	0	0	0	0
0	0	0	0	0	4,262E-06	0
0	0,000075168	0	5,938E-05	0	0	0
0	0	0,00000957	1,425E-05	0	0	0
0	0,00000864	0	4,513E-05	0	0	0
0	0	0	0	0	0	0
0	0	0	0	0	0	0
0,000012342	0	0	3,088E-05	0	1,0655E-05	7,722E-06
0	0	0,00000957	0	0	4,262E-06	0
0	0	0,00000957	0	0	4,262E-06	7,722E-06
0	0	0	1,425E-05	0,00005141	1,0655E-05	0
0	0,000025056	0	0	0	0	0
0	0	0	0	0	0	0
0	0,00000864	0,00003828	1,425E-05	0	4,262E-06	0
0	0	0,00000957	0	0	0	0
0	0,00000864	0,00000957	1,425E-05	0,00000485	0,00002131	7,722E-06
0	0	0,00012441	0	0	0	0
0,000006171	0,00000864	0,00001914	0	0,00000485	4,262E-06	0
0	0	0	0	0	0	0
0	0	0	0	0	0	0
0	0,000025056	0	1,425E-05	0,00001067	0	0
0	0	0	0,000076	0	0	0
0	0	0	0	0	0	0
0	0	0	0	0	0	0
0	0	0	0	0,00002619	0	0
0	0,000016416	0,00001914	0	0	1,4917E-05	0
0	0	0	0	0	0	0
0	0	0	1,425E-05	0	0	0
0	0	0,00000957	0	0	0	0
0	0	0	5,938E-05	0	0	0
0	0	0	0,0002114	0	0	0

0	0	0	0	0	0	0
0	0	0	0	0	0	0
0	0	0	0	0	0	0
0	0	0,00005742	0	0	0	0
0	0	0	0	0	0	0
0	0	0,00001914	0	0,00001552	0	0
0	0	0	0	0	4,262E-06	0
0	0	0	0	0	0	0
0	0	0	0	0	0	0,0001432
0	0	0	0	0	0	0
0	0,000016416	0	0	0	0	0
0	0	0	0	0,00005141	0	0
0	0	0,00001914	0	0	4,262E-06	0
0	0	0,00000957	0	0	0	0
0	0	0	0	0	0	0
0	0	0	0	0	0	0
0	0	0	0	0	0	0
0	0	0	0	0	0	0
0	0	0,00002871	0	0	0	0
0	0,000050112	0,00002871	4,513E-05	0	0	0
0,000006171	0	0	0	0	0	0
0	0	0	1,425E-05	0	0,00022376	0
0	0	0	0	0	0	0
0	0	0	1,425E-05	0,00000485	0	0
0	0	0	0	0	0	0
0	0	0	0	0	0	0
0	0,000016416	0	5,938E-05	0	0	0
0	0	0,00000957	0	0	0	0
0	0,000050112	0	0	0	0	0
0	0	0,00004785	0	0	0	0
0	0	0	0	0	0	0
0,000055176	0,000025056	0	0	0,00001067	1,4917E-05	3,98E-05
0	0,000016416	0,00000957	1,425E-05	0	0	0
0	0	0	0	0,00001552	0	0
0	0	0,00000957	0	0	0	0
0	0,000175392	0,00001914	0	0	0	0
0	0	0	0	0	4,262E-06	0
0	0	0,00004785	0	0,00000485	0	0
0	0	0,00000957	0	0	0	0
0	0	0	0	0	0	0
0	0	0,00003828	0	0	4,262E-06	0
0,000042834	0	0	0	0,00006693	0	0
0	0	0	0	0	0	0
0	0	0	0	0	0	0
0	0	0	0	0	0	0
0	0	0,00002871	0	0	1,0655E-05	0
0	0	0	0	0	0	0
0	0,00000864	0,00002871	0	0	0	0
0	0	0	3,088E-05	0	4,262E-06	0
0	0	0	0	0	0	0
0	0	0	0	0	0	0
0	0	0	0	0	0	0,0001271
0	0	0	0	0	0	0
0	0	0,00003828	0	0	0	0
0,000037026	0,000041472	0	0	0,0000776	0	0

0	0	0	0	0	0	0
0	0	0,00005742	1,425E-05	0	1,0655E-05	0
0,000006171	0	0,00000957	0	0	0	0
0	0	0	0	0	0	0
0	0	0	0	0	0	0
0	0	0	0	0	0	0
0	0,00000864	0	0	0	4,262E-06	0
0	0	0	0	0	0	0
0	0	0	0	0	0	0
0	0	0	0	0	0	0
0,000006171	0	0	0	0	0	0
0	0	0	0	0	0	0
0	0	0,00000957	0	0	0	0
0	0,000033696	0,00000957	0	0,00000485	0	0
0	0,000083808	0	0	0	0	0
0	0,000191808	0	0	0	0	0
0	0	0	0	0	0	0
0,000012342	0,000016416	0	1,425E-05	0,00001552	1,0655E-05	0
0	0	0	0	0	0	0
0	0	0	1,425E-05	0	0,00002131	0
0,000037026	0	0	0	0,00002037	1,0655E-05	2,376E-05
0,000018513	0,000016416	0,00000957	0	0,00000485	0	0
0	0	0	4,513E-05	0	0	0
0	0	0,00000957	0	0	0	0
0,000012342	0	0	0	0,00002037	0	2,376E-05
0	0	0,00001914	0	0	0	0
0	0,00000864	0	1,425E-05	0	4,262E-06	0
0	0	0,00000957	0	0	0	0
0	0	0,00000957	0	0	0	0
0	0	0	0	0	0	0
0	0	0,00008613	0	0	0	0
0	0,00000864	0	0,000076	0	0	0
0	0	0	0	0	0	0
0	0	0	0	0	0	0
0	0	0	0	0	0,00002131	0
0	0,00000864	0,00003828	0	0	4,262E-06	0
0,000006171	0	0	0	0,00005141	2,5572E-05	0
0	0,000033696	0	0	0	0	0
0	0	0,00000957	0	0	0	0
0	0,00000864	0	1,425E-05	0	0	0
0	0	0	1,425E-05	0	0	0
0	0	0	0	0	0	0
0	0	0	0	0	5,1144E-05	1,604E-05
0,000006171	0	0	3,088E-05	0	0	0
0	0	0	0	0	0	0
0	0	0	0	0	0	0
0	0	0	0	0	0	0
0	0	0	0	0	0	0
0	0	0	0	0	0	0,0002382
0	0	0	0	0	0	0
0	0	0	0	0	0	0
0	0	0,00000957	0	0	0	7,722E-06
0	0	0	0	0	0	0
0,000116523	0	0	0	0	0	0
0	0	0	0	0,00003104	2,5572E-05	1,604E-05

0	0	0,00002871	3,088E-05	0	0	0
0	0,000025056	0	0	0	0	0
0	0	0	0	0	0	0
0	0	0	0	0	0	0
0	0	0	0	0	0	0
0	0,000016416	0	0	0	0	0
0	0	0	0	0	0	0
0	0	0,00003828	0	0	0	0
0	0,000033696	0	3,088E-05	0	0	0
0	0	0	0	0	0	0
0	0	0,00000957	0	0	0	0
0	0,00000864	0	0	0	0	7,722E-06
0	0	0	0	0	0	0
0	0	0	0	0	0	0
0	0,000016416	0	0	0	0	0
0	0	0	0	0	0	0
0	0	0	0	0	0	0
0	0	0,00000957	1,425E-05	0	0	0
0	0	0	0	0	0	0
0,000006171	0	0	0	0	4,262E-06	0
0	0,00000864	0,00000957	0	0	0	0
0	0	0	0	0,00006693	0	0
0	0	0	0	0	0	0
0	0	0	0	0	0	0
0	0	0	0	0	4,262E-06	0
0	0	0	0	0	0	0
0	0	0	0	0,00000485	0	0
0	0	0,00000957	0	0	0	0
0	0	0,00000957	1,425E-05	0	0	7,722E-06
0	0,000050112	0	0	0	0	0
0	0	0,00002871	5,938E-05	0	0	0
0	0	0	0	0	0,00002131	0
0	0	0	0	0	0	0
0	0	0,00008613	0	0	0	0
0	0,00000864	0	0	0	0	0
0,000006171	0,000016416	0	0	0	0	7,722E-06
0	0	0	0	0	0	0
0	0	0	0	0	0	0
0	0	0	0	0	0	0
0	0	0,00001914	0	0	0	0
0,000006171	0,000033696	0	1,425E-05	0	4,262E-06	7,722E-06
0	0	0,00001914	1,425E-05	0	0	0
0,000037026	0	0	0	0,00012416	1,0655E-05	0
0	0	0,00000957	0	0	0	0
0	0	0,00005742	0	0	0	0
0	0	0	0	0	0	0
0	0	0	0	0	0	0
0	0	0	0	0	0	0
0	0	0	1,425E-05	0	0	0
0	0	0	0	0	0	0
0	0	0,00000957	0	0	0	0
0	0	0	0	0	0	0
0	0	0	0	0	0	0
0	0	0	0	0	0	0
0	0	0	0	0	0	0,0001034

0	0	0	0	0	0	0
0	0	0	0	0,00001552	0	0
0	0	0	0	0	0	0
0,000067518	0	0	0	0	0	0
0	0	0	0	0	0	0
0,000012342	0,000033696	0	0	0	1,4917E-05	7,96E-05
0	0	0	0	0	0	2,376E-05
0	0	0	0	0	0	0
0	0	0	0	0	0	0
0	0	0	0	0	0	0
0	0	0	0	0	0	0
0,000018513	0	0,00000957	0	0	0	7,722E-06
0	0	0	0	0	0	0
0	0	0,00000957	0	0	1,0655E-05	0
0	0	0	0	0,00000485	0	0
0	0,000033696	0	0	0,00003104	0	3,98E-05
0	0	0,00000957	0	0	0	0
0	0	0	0	0,00001067	0	0
0	0	0	0	0	0	0
0	0	0	0	0	0	0
0	0	0	0	0	0	0
0	0	0	0	0,00000485	0	0
0	0	0	4,513E-05	0	0	0
0	0,000016416	0	0	0	0	0
0	0	0	0	0	0	0
0	0	0	0	0	0	0
0	0	0	0	0	0	0
0	0	0	0	0	0	0
0	0,00000864	0	0	0	0	0
0	0	0	9,025E-05	0	0	0
0	0	0,00000957	0	0	0	0
0	0	0	0	0	0	0
0	0	0	0	0	0	0
0	0	0	0	0	0	0
0	0	0	0	0	0	0
0	0	0	0	0	0	0
0	0	0	0	0	0	0
0	0	0	0	0	0	1,604E-05
0	0	0	0	0	0	0
0	0	0	0	0,00002619	0	0
0	0	0	0	0,00000485	0	0
0	0	0	4,513E-05	0	1,4917E-05	0
0	0	0	0	0	0	0
0	0	0	0	0	0	0
0	0	0	0	0	0	0
0	0	0	0	0	0	0
0	0	0	0	0	0	0
0	0	0,00000957	0	0	0	0
0	0	0	0	0	0	0
0	0	0	0	0	0	0
0	0	0	0	0	0	0
0	0	0	0	0,00000485	0	0
0	0	0	0	0	0	0
0	0	0	0	0	0	0
0	0	0	0	0	0	0

0	0	0	0	0	0	0
0	0	0	0	0	0	0
0	0	0	0	0	4,262E-06	0
0	0,00000864	0	1,425E-05	0	0	0
0	0	0	0	0	0	0
0	0	0	0	0	0	0
0	0	0	0	0,00000485	4,262E-06	0
0	0	0	0	0	0	0
0	0	0	0	0	0	0
0	0,00000864	0	0	0	0	0
0	0	0	0	0	0	0
0	0	0	3,088E-05	0	0	0
0	0,00000864	0	0	0	0	0
0,000141207	0	0	0	0	0	0
0	0	0,00001914	0	0	0	0
0	0	0	0	0	0	0
0	0	0,00000957	0	0	0	0
0	0	0,00002871	0	0	4,262E-06	0
0	0	0	0	0	0	0
0	0	0	0	0	0	0
0	0	0	0	0	0	0
0	0,00000864	0	0	0	0	0
0	0	0,00001914	0	0,00000485	0	0
0	0	0	0	0	0	0
0	0	0	1,425E-05	0	0	0
0	0	0	0	0	0	0
0	0	0,00000957	0	0	0	0
0	0	0,00002871	0	0	0,00002131	0
0	0	0	0	0	0	0
0	0	0	3,088E-05	0	0	0
0	0	0	0	0	0	0
0	0	0	1,425E-05	0	0	0
0	0	0	0	0	0	0
0	0	0	0	0	0	0
0	0	0	0	0	0	0
0	0	0	0	0	0	0,0001271
0	0	0	0	0	0	0
0,000061347	0	0	0	0	0	1,604E-05
0,000018513	0,000016416	0	0	0	0	0
0	0	0	0	0	0	0
0	0	0	0	0	0	0
0	0	0	0	0	0	0
0	0	0	0	0	0	0
0	0,00000864	0	0	0	0	0
0,000104544	0	0	0	0	0	0
0	0	0	0	0	0	0
0	0	0	0	0	0,00012147	0
0	0,00000864	0	4,513E-05	0	0	0
0	0	0	0	0,00001067	2,9834E-05	0
0	0	0	0	0	0	0
0	0	0,00000957	0	0,00001067	0	0
0	0,000016416	0	0	0	0	0
0	0	0,00000957	0	0	0	0
0	0,000025056	0	0	0	0	0
0	0	0	1,425E-05	0	0	0

0	0	0	0	0	0	0
0	0	0	0	0	0	0
0	0	0	0	0	0	0
0	0,00000864	0	0	0	0	7,722E-06
0	0	0	0	0	0	7,722E-06
0	0	0	0	0	0	0
0	0	0	0	0	0	0
0	0	0	0	0	0	0
0	0	0	0	0	0	0
0	0	0	0	0	0	7,722E-06
0	0	0	0	0	0	0
0	0	0	0	0	0	0
0	0	0	0	0	0	0
0	0	0	0	0	0	0
0	0	0	0	0	0	0
0	0,00000864	0	0	0	0	7,722E-06
0	0	0	3,088E-05	0	0	0
0	0,00000864	0	0	0	0	0
0	0	0,00000957	0	0	0	1,604E-05
0	0	0	0	0	0	0
0	0	0	1,425E-05	0	0	0
0	0	0	1,425E-05	0	0	0
0	0	0	0	0	0	0
0	0	0	0	0	0	0
0,000067518	0	0,00001914	0	0	0	0
0	0	0	0	0	0	0
0	0	0,00000957	0	0,00000485	4,262E-06	0
0,000012342	0,000025056	0,00004785	0	0	0	0
0	0	0	0	0	0	0
0	0	0	0	0	0	0
0	0	0	0	0	0	0
0	0	0	0	0	3,6227E-05	0
0	0	0	0	0	0	0
0	0	0	1,425E-05	0	0	0
0	0	0	0	0	0	0
0	0	0	0	0	0	0
0	0	0	1,425E-05	0	0	0
0	0	0	0	0	0	0
0	0	0,00001914	0	0	0	0
0	0	0	0	0	0	0
0	0	0,00000957	0	0	0	0
0	0	0	0	0	0	0
0	0	0	0	0	0	0
0	0	0	0	0	0	0
0	0	0	0	0	0	0
0	0	0,00001914	0	0	0	0
0	0	0,00003828	0	0	0	0
0	0	0	0	0	0	0
0	0	0,00000957	0	0	0	0
0	0	0	1,425E-05	0	0	0
0	0	0,00002871	0	0	0	0
0	0	0	0	0	0	0
0	0	0	0	0	0	0
0	0	0	0	0	0	0
0	0	0	0	0	0	0

0	0	0	1,425E-05	0	0	0
0,000006171	0	0	0	0	0	0
0	0	0	0	0	0	0
0	0	0	0	0	0	0
0,000006171	0	0,00007656	0	0	0	0
0	0,00000864	0	0	0	0	0
0	0	0,00003828	0	0	0	0
0	0	0	0	0	0	0
0	0,00000864	0	0	0	0	0
0	0,00000864	0	0	0	0	0
0	0,000016416	0,00000957	0	0	0	0
0	0	0	0	0	0	0
0	0	0	0	0	0	0
0	0	0,00001914	0	0,00000485	0	0
0	0	0	0	0	0	0
0	0	0	0	0	0	0
0	0	0	0	0	0	0
0	0	0	0	0	0	0
0	0	0	0	0	4,262E-06	0
0	0	0	0	0	0	0
0	0	0	0	0	0	0
0	0	0	0	0	0	0
0	0,000075168	0	0	0	0	0
0	0,000016416	0	1,425E-05	0	0	0
0	0	0	1,425E-05	0	0	0
0	0	0	0	0	0	0
0	0	0	0	0	0	0
0	0	0	0	0	0	0
0	0	0,00001914	0	0	0	0
0	0	0	0	0	0	0
0	0	0	0	0	0	0
0	0	0	0	0	0	0
0	0	0	0	0	0	0
0,000006171	0,00000864	0	0	0,00000485	1,0655E-05	0
0	0	0,00000957	3,088E-05	0	0	0
0	0	0,00001914	0	0	0	0
0	0	0	0	0	4,262E-06	0
0	0	0	0	0	0	0
0	0	0	0	0	4,262E-06	0
0	0	0	0	0	0	0
0	0	0	0	0	0	0
0	0,00000864	0	0	0	0	0
0	0,00000864	0,00000957	0	0	0	0
0	0	0	0	0	0	0
0	0	0	0	0,00000485	0	0
0	0	0	0	0	0	0
0	0,00000864	0	0	0	0	0
0	0	0	0,000076	0	0	0
0	0	0	0	0	0	0
0	0	0	0	0	0	0
0	0,00000864	0	0	0	0	0
0	0	0	0	0	0	0
0	0	0	0	0	0	0
0	0	0	0	0	0	0
0,000006171	0	0	0	0,00000485	0	0

0	0	0	0	0	0	0
0	0	0	0	0	0	0
0	0	0	0	0	0	0
0	0	0	0	0	0	0
0	0	0	0	0	0	0
0	0	0	0	0	0	0
0	0	0	0	0,00000485	0	0
0	0	0	0	0	0	0
0	0	0	0	0	0	0
0	0	0	0	0	0	0
0	0	0	0	0	0	0
0	0	0	0	0	0	0
0	0	0	0	0	0	0
0	0	0,00000957	0	0	0	0
0	0	0	0	0	0	0
0	0	0	0	0	0	0
0	0	0	0	0	0	0
0	0	0	0	0	0	0
0	0	0	0	0	4,262E-06	0
0	0	0	1,425E-05	0	0	0
0	0	0	1,425E-05	0	0	0
0,000049005	0	0	0	0	0	0
0,000006171	0	0	1,425E-05	0,00001552	4,262E-06	7,722E-06
0	0	0	0	0	0	0
0	0	0	0	0	0	0
0	0	0	0	0	0	0
0	0	0	0	0	0	0
0	0	0	0	0,00004656	0	0
0	0	0	0	0	0	0
0	0	0,00003828	0	0	0	0
0	0	0	0	0	0	6,356E-05
0	0	0	0	0	0	0
0	0	0	0	0	0	0
0	0	0,00000957	0	0,00000485	0	0
0	0	0	0	0	0	0
0	0	0	0	0	0	0
0	0	0	0	0	0	0
0	0	0	0	0	4,262E-06	0
0	0	0	0	0	0	0
0	0	0	0	0	0	0
0	0	0	0	0	0	0
0	0	0	0	0,00000485	0	0
0	0	0	0	0	0	0
0	0	0	0	0	0	0
0	0	0	0	0	0	0
0	0	0	5,938E-05	0	0	0
0	0	0	0	0	0	0
0	0	0	0	0	0	0
0	0,000016416	0	0	0	0	0
0	0	0	0	0	0	0
0	0	0	0	0	0	0
0	0	0	0	0	0	0
0	0	0	0	0	0	0
0	0	0	0	0	2,5572E-05	3,208E-05
0	0	0	0	0	0	0

0	0	0	0	0	0	0
0	0	0,00003828	0	0	0	0
0	0	0	0	0	1,4917E-05	0
0	0	0,00000957	0	0	0	0
0	0	0	0	0	0	0
0	0	0	0	0	0	0
0	0	0	0	0	0	0
0	0	0	0	0	0	0
0	0	0,00004785	0	0	0	0
0	0	0	0	0	0	0
0	0	0	0	0	0	0
0,000006171	0	0	0	0	0	0
0	0	0	0	0	0	0
0	0	0	0	0	0	0
0	0	0	0	0	0	0
0	0	0	0	0	0	0
0	0	0,00001914	0	0	0	0
0	0	0	0	0	0	0
0	0	0	0	0	0	0
0	0	0	0	0	0	0
0	0	0,00000957	0	0	0	0
0	0	0	0	0	4,262E-06	0
0	0,000033696	0	0	0	0	0
0	0	0	0	0	0	0
0	0	0	0	0	0	0
0	0	0	0	0	0	0
0	0	0	0	0	0	0
0	0	0	0	0	0	0
0	0,00000864	0	0	0	0	0
0	0	0,00000957	0	0	0	0
0	0	0	0	0,00000485	0	0
0	0	0	1,425E-05	0	0	0
0	0	0	0	0	0	0
0	0	0	0	0	0	0
0	0	0	0	0	0	0
0	0	0	0	0	0	0
0	0	0	0	0	0	0
0	0	0	0	0	4,262E-06	0
0	0	0	1,425E-05	0	0	0
0	0	0	0	0	0	0
0	0	0	0	0	0	0
0	0	0	3,088E-05	0	0	0
0	0	0,00001914	0	0	0	0
0	0	0	0	0	0	0
0	0	0	0	0	0	0
0	0	0	0	0	0	0
0	0	0	0	0	0	0
0	0	0	0	0	0	0
0	0	0	0	0	0	0
0	0	0	0	0	0	0
0	0	0	0	0	0	0
0	0	0	0	0	0	0
0	0	0	0	0	0	0
0,000049005	0	0	0	0	0	0
0	0	0	0	0	1,0655E-05	0

0	0	0	0	0	0	0
0,000030855	0	0	0	0	0	0
0	0	0	0	0	0	0
0	0	0	0	0	0	4,752E-05
0	0	0	0	0	0	0
0	0	0	0	0	0	0
0	0	0	1,425E-05	0	0	0
0	0	0	0	0	0	0
0	0	0	0	0	0	0
0	0	0	0	0	0	0
0	0	0	0	0	0	0
0	0	0	0	0	0	0
0	0	0,00000957	1,425E-05	0	0	0
0	0	0	0	0	0	0
0	0	0	0	0	0	0
0	0	0	4,513E-05	0	0	0
0,000024684	0	0	0	0	0	0
0	0	0	0	0	0	0
0	0	0	0	0	0	0
0	0	0	0	0	0	0
0	0	0	0	0	0	0
0	0	0	1,425E-05	0	0	0
0	0	0	0	0	0	0
0	0	0	0	0	0	0
0	0	0	0	0	4,262E-06	0
0	0	0	0	0	0	0
0	0	0	0	0	0	0
0	0	0	0	0	0	0
0	0	0	0	0	0	0
0	0	0	0	0	0	0
0	0	0	0	0	0	0
0,000042834	0	0	0	0	0	0
0,000042834	0	0	0	0	0	0
0,000042834	0	0	0	0	0	0
0	0	0	0	0	0	0
0	0	0	0	0	0	0
0	0	0	0	0	0	0
0	0	0	0	0	0	0
0	0	0	0	0	0	0
0	0	0	0	0	0	0
0	0	0	0	0	0	0
0	0	0	0	0,00004171	0	0
0,000024684	0	0	0	0	0	0
0	0	0	1,425E-05	0	0	0
0	0	0	0	0	0	0
0	0	0	0	0	0	0
0	0	0,00001914	0	0	0	0
0	0	0	0	0	0	0
0,000030855	0	0	0	0	0	0
0	0	0	0	0	0	0
0	0	0	0	0	0	0
0	0	0	0	0	0	0
0	0	0	0	0	0	0
0	0	0	0	0	0	0
0	0	0	0	0	0	0

0	0	0	0	0	0	0
0	0	0,00000957	0	0	0	0
0	0	0,00000957	0	0	0	0
0	0	0	0	0	4,262E-06	0
0	0	0	0	0,00000485	4,262E-06	7,722E-06
0	0	0	0	0	0	0
0	0	0	0	0	0	0
0	0	0	1,425E-05	0	0	0
0	0,00000864	0	0	0	0	0
0	0	0	0	0	0	0
0	0	0,00001914	0	0	0	0
0	0	0	0	0	0	0
0	0	0	0	0	0	0
0	0	0	0	0	0	0
0	0,00000864	0	0	0	0	0
0	0	0	0	0	0	0
0	0	0,00000957	0	0	0	0
0	0	0	0	0	0	0
0	0	0	0	0	0	0
0	0	0	0	0	0	0
0	0	0	0	0	0	0
0	0	0	0	0	0	0
0	0	0,00002871	0	0	0	0
0	0	0	0	0	0	0
0	0	0	0	0	0	0
0	0	0	0	0	0	7,722E-06
0	0	0	0	0	0	0
0	0	0	0	0	0	0
0	0	0	0	0	0	0
0	0	0	0	0	0	0
0	0	0	0	0	0	0
0	0	0	0	0	0	0
0	0,00000864	0	0	0	0	0
0	0	0	0	0	0	0
0	0	0	0	0	0	0
0	0	0	0	0	0	0
0	0	0	0	0	0	0
0	0	0	0	0	0	0
0	0	0	0	0	0	0
0,000024684	0	0	0	0	0	0
0	0	0	0	0	0	0
0	0	0	0	0,00000485	0	0
0	0	0	0	0	0	0
0	0	0	1,425E-05	0	0	0
0	0,00000864	0	0	0	0	0
0	0	0	0	0	0	0
0	0	0	0	0,00001067	0	0
0,000006171	0	0	0	0,00001067	0	0
0	0	0	0	0	0	0
0	0,00000864	0	0	0	0	0
0	0	0	0	0	0	0
0	0	0	0	0	0	0
0	0	0	1,425E-05	0	0	0
0	0	0	0	0	0	0
0,000030855	0	0	0	0	0	0

0	0	0	0	0	0	0
0	0	0	0	0	0	0
0	0	0	0	0	0	0
0,000006171	0	0	0	0	0	0
0,000012342	0	0	0	0	0	0
0	0	0	0	0	0	0
0	0	0	0	0	0	0
0	0	0	0	0	0	0
0	0	0	0	0	0	0
0	0	0	0	0	0	0
0	0	0	0	0	0	0
0	0	0	0	0	0	0
0	0	0	0	0	0	0
0	0	0	0	0,00001552	0	0
0	0	0	0	0	0	0
0	0	0	0	0	0	0
0	0	0	0	0	0	0
0	0	0	0	0	0	0
0	0	0	0	0	0	0
0	0	0	0	0	0	0
0	0	0	0	0	0	0
0	0	0	0	0	0	0
0	0	0	0	0	0	0
0	0	0	0	0	0	0
0	0	0	0	0	0	0
0	0	0	0	0	0	0
0	0	0,00002871	0	0	0	0
0	0	0,00000957	0	0	0	0
0	0	0,00001914	0	0	0	0
0	0	0,00001914	0	0	0	0
0	0	0	0	0	0	0
0	0	0	0	0	0	0
0	0	0	0	0	0	0
0	0	0	0	0	0	0
0	0	0	0	0	0	0
0	0	0	0	0	0	0
0	0	0	0	0	0	0
0	0	0	0	0	0	0
0	0	0	0	0	0	0
0	0	0	0	0	0	0
0	0	0	0	0	0	0
0	0	0	0	0	0	0
0	0	0	0	0	0	0
0	0	0	0	0	0	0
0	0	0	0	0	0	0
0	0	0	0	0	0	0
0	0	0	0	0	0	0
0	0	0	0	0	0	0
0	0	0	0	0	0	0
0	0	0	0	0	0	0
0	0	0	0	0	0	0
0	0	0	0	0	0	0
0	0	0	0	0	0	0
0	0	0,00000957	0	0	0	0
0	0	0	0	0	0	0

0	0	0	0	0	0	0
0	0	0	0	0	0	0
0	0	0	0	0	0	0
0	0	0	0	0	0	0
0	0	0	0	0	0	0
0	0	0	0	0	0	0
0	0	0	0	0	1,4917E-05	0
0	0	0	0	0	0	0
0	0	0	0	0	0	0
0	0	0	0	0	0	0
0	0	0	0	0	0	0
0	0	0	0	0	0	0
0	0	0	0	0	0	0
0	0	0	0	0	0	0
0	0	0	0	0	0	0
0	0,000016416	0	0	0	0	0
0	0	0	0	0	0	0
0	0	0	0	0	0	0
0	0	0	0	0	0	0
0	0	0	0	0	0	0
0	0	0	0	0	0	0
0	0	0	0	0	0	0
0	0	0	0	0	0	0
0	0	0	0	0	0	0
0	0	0	0	0	0	0
0	0	0	0	0	0	0
0	0	0	0	0	0	0
0	0	0	0	0	0	0
0	0	0	0	0	0	0
0	0	0	0	0	0	0
0	0	0	0	0	0	0
0	0	0	0	0	0	0
0	0	0,00000957	0	0	0	0
0	0	0,00000957	0	0	0	0
0	0	0,00000957	0	0	0	0
0	0	0,00000957	0	0	0	0
0	0	0	0	0,00001552	0	0
0	0	0	0	0	0	0
0	0	0,00000957	0	0	0	7,722E-06
0	0	0	0	0	0	0
0	0	0	0	0	0	0
0	0	0	0	0	0	0
0	0	0	0	0	0	0
0	0	0	0	0	0	0
0	0	0	0	0	0	0
0	0	0	0	0	0	0
0	0	0	0	0	0	0
0	0	0	0	0	0	0
0	0	0	0	0	0	0
0	0	0	0	0	0	0
0	0	0	0	0	0	0
0,000012342	0	0	0	0	0	0
0	0	0,00000957	1,425E-05	0	0	0
0	0	0	0	0	0	2,376E-05
0	0	0	0	0	0	2,376E-05

0	0	0	0	0,00001067	0	0
0	0	0	0	0	0	0
0	0	0	1,425E-05	0	0	0
0	0	0	1,425E-05	0	0	0
0	0	0	0	0	0	0
0	0	0	1,425E-05	0	0	0
0	0	0	1,425E-05	0	0	0
0	0	0	1,425E-05	0	0	0
0	0	0	0	0	0	0
0	0	0	0	0	0	0
0	0	0	0	0	0	0
0	0,00000864	0	0	0	0	0
0	0	0	0	0	0	0
0	0	0	0	0	0	0
0	0	0	1,425E-05	0	0	0
0	0	0	0	0	0	0
0	0	0	0	0	0	0
0	0	0	0	0	0	0
0	0	0	0	0	0	0
0	0	0	0	0	0	0
0	0	0	0	0	0	0
0	0	0	0	0	0	0
0	0	0	0	0	0	0
0	0	0	0	0	0	0
0	0	0	0	0	0	0
0	0	0	0	0	0	0
0	0	0	0	0	0	0
0	0	0	0	0	0	0
0	0	0	0	0	0	0
0	0	0	0	0	0	0
0	0	0	0	0	4,262E-06	0
0	0	0	0	0	0	0
0	0	0	0	0	0	0
0	0	0	0	0	1,0655E-05	0
0	0	0	0	0	0	0
0	0	0,00000957	0	0	0	0
0	0	0	0	0	0	0
0	0	0	0	0	0	0
0	0	0	0	0	0	0
0	0	0	0	0	0	0
0	0	0	0	0	0	0
0	0	0	0	0	0	0
0	0	0	0	0	0	0
0	0	0	0	0	0	0
0	0	0	0	0	0	0
0	0	0	0	0	0	0
0	0	0	0	0	0	0
0	0	0	0	0	0	0
0	0	0	0	0	0	0
0	0	0	0	0	0	0
0	0	0,00001914	0	0	0	0
0	0	0	0	0	0	0
0	0	0	0	0	0	0
0	0	0	0	0	0	0

0	0	0,00000957	0	0	0	0
0	0	0	0	0	0	0
0	0	0,00000957	0	0	0	0
0,000012342	0	0	0	0	0	0
0	0	0	0	0	0	0
0	0	0	0	0	0	0
0	0	0	0	0	0	0
0	0	0,00000957	0	0	0	0
0	0	0	0	0	0	0
0	0	0	0	0	0	0
0	0	0	0	0	0	0
0	0	0	0	0	0	0
0	0	0	0	0	0	0
0	0	0	0	0	0	0
0	0	0	0	0	0	0
0	0	0	0	0	0	0
0	0	0	0	0	0	0
0,000018513	0	0	0	0	0	0
0	0	0	0	0	0	0
0	0	0	0	0	0	0
0	0	0	0	0	0	0
0	0	0	0	0	0	0
0	0	0	0	0	0	0
0	0	0	0	0	0	0
0	0	0,00000957	0	0	0	0
0	0	0	0	0	0	0
0	0	0	0	0	0	0
0	0	0	0	0	0	0
0	0	0	0	0	0	0
0	0	0	0	0	0	0
0	0	0	0	0	0	0
0	0	0	0	0	0	0
0	0	0	0	0	0	0
0,000012342	0	0	0	0	0	0
0	0	0	0	0	0	0
0	0	0,00000957	0	0	0	0
0	0	0	0	0	0	0
0	0	0	0	0	0	0
0	0	0	0	0	0	0
0	0	0	0	0	0	0
0	0	0	0	0	0	0
0	0	0	0	0	0	0
0	0	0	0	0	0	0
0	0	0	0	0	0	0
0	0	0	0	0	0	0
0	0	0	0	0	0	0
0	0	0	0	0	0	0
0	0	0	0	0	0	0
0	0	0	0	0	0	0
0	0	0	0	0	0	0
0	0	0	0	0	0	0
0	0	0	0	0	0	0
0	0	0	0	0	0	0
0	0	0	0	0	0	0
0	0	0	0	0	0	0

0	0	0	0	0	0	0
0	0	0	0	0	0	0
0	0	0	0	0	0	0
0	0	0,00000957	0	0	0	0
0	0	0,00000957	0	0	0	0
0	0	0,00000957	0	0	0	0
0	0	0	0	0	0	0
0	0	0	0	0	0	0
0	0	0	0	0	0	0
0	0	0	0	0	0	0
0	0,000016416	0	0	0	0	0
0	0,000016416	0	0	0	0	0
0	0	0	0	0	0	0
0	0	0	0	0	0	0
0	0	0	0	0	0	0
0	0	0	0	0	0	0
0	0	0	0	0	0	1,604E-05
0	0	0	0	0	0	0
0	0	0	0	0	0	0
0	0	0	0	0	0	0
0	0	0	0	0	0	0
0	0	0	0	0	0	0
0	0	0	0	0	0	0
0	0	0	0	0	0	0
0	0	0	0	0	0	0
0	0	0	0	0	0	0
0	0	0	0	0	0	0
0	0	0	0	0	0	0
0	0	0	0	0	0	0
0	0	0	0	0	0	0
0	0	0	0	0	0	0
0	0	0	0	0	1,4917E-05	0
0	0	0	0	0	0	0
0	0	0	0	0	0	0
0	0	0	0	0	0	0
0	0	0	0	0	0	0
0	0	0	0	0	0	0
0	0	0	0	0	0	0
0	0	0	0	0	0	0
0	0	0	0	0	0	0
0	0	0	0	0	0	0
0	0	0	0	0	0	0
0	0	0	0	0	0	0
0	0	0	0	0	0	0
0	0	0	1,425E-05	0	0	0
0	0	0	1,425E-05	0	0	0
0	0	0	1,425E-05	0	0	0
0	0	0	1,425E-05	0	0	0
0	0	0	1,425E-05	0	0	0
0	0	0	1,425E-05	0	0	0
0	0	0	1,425E-05	0	0	0
0	0	0	0	0	0	0
0	0	0	0	0	0	0
0	0	0	0	0	0	0
0	0	0	0	0	0	0

0	0	0	0	0	0	0
0	0	0	0	0	0	0
0	0	0	0	0	0	0
0	0	0	0	0	0	0
0	0	0	0	0	0	0
0	0	0	0	0	0	0
0	0	0	0	0	0	0
0	0	0	0	0	0	0
0	0	0	0	0	0	0
0	0	0	0	0	0	0
0	0	0	0	0	0	0
0	0	0	0	0	0	0
0	0	0	0	0	0	0
0	0	0	0	0	0	0
0	0	0	0	0	0	0
0	0	0	0	0	0	0
0	0	0	0	0	4,262E-06	0
0	0	0	0	0	0	0
0	0	0	0	0	0	0
0	0	0	0	0	0	0
0	0	0	0	0	0	0
0	0	0	0	0	0	0
0	0	0	0	0	0	0
0	0	0	0	0	0	0
0	0	0	0	0	0	0
0	0	0	0	0	0	0
0	0	0	0	0	0	0
0	0,00000864	0	0	0	4,262E-06	0
0	0	0	0	0	0	0
0,000012342	0	0	0	0	0	0
0,000012342	0	0	0	0	0	0
0	0	0	0	0	0	0
0,000006171	0	0	0	0	0	0
0	0	0	0	0	1,0655E-05	0
0	0	0	0	0	1,0655E-05	0
0	0	0	0	0	0	0
0	0	0	0	0	0	0
0	0	0	0	0	0	0
0	0	0	0	0	0	0
0	0	0	0	0	0	0
0	0	0	0	0	0	0
0	0	0	0	0	0	0
0	0	0	0	0	0	0
0	0	0	0	0	0	0
0	0	0	0	0	0	0
0	0	0	0	0	0	0
0	0	0	0	0	0	0
0	0	0	0	0	0	0
0	0	0	0	0	0	0
0	0	0	0	0	0	0
0	0	0	0	0	0	0
0	0	0	0	0	0	0
0	0	0	0	0	0	0
0	0	0	0	0	0	0
0	0	0	0	0	0	0

0	0	0	0	0	0	0
0	0	0	0	0	0	0
0	0	0	0	0	0	0
0	0	0	0	0	0	0
0	0	0,00000957	0	0	0	0
0	0	0,00000957	0	0	0	0
0	0	0,00000957	0	0	0	0
0	0	0,00000957	0	0	0	0
0	0	0,00000957	0	0	0	0
0	0	0,00000957	0	0	0	0
0	0	0,00000957	0	0	0	0
0	0	0	0	0	0	0
0	0	0	0	0	0	0
0	0	0	0	0	0	0
0	0	0	0	0	0	0
0	0	0	0	0	0	0
0	0	0	0	0	0	0
0	0	0	0	0	0	0
0	0	0	0	0	0	0
0	0	0	0	0	0	0
0	0	0	0	0	0	0
0	0	0	0	0	0	0
0	0	0	0	0	0	0
0	0	0	0	0	0	0
0	0	0	0	0	0	0
0	0	0	0	0	0	0
0	0	0	0	0	0	0
0	0	0	0	0	0	0
0	0	0	0	0	0	0
0	0	0	0	0	0	0
0	0	0	0	0	0	0
0	0	0	0	0	0	0
0	0	0	0	0	0	0
0	0	0	0	0	0	0
0	0	0	0	0,00000485	0	0
0	0	0	0	0	0	0
0	0	0	0	0	0	0
0	0	0	0	0	0	0
0	0	0	0	0	0	0
0	0	0	0	0	0	0
0	0	0	0	0	0	0
0	0	0	0	0	0	0
0	0	0	0	0	0	0
0	0	0	0	0	0	0
0	0	0	0	0	0	0
0	0	0	0	0	0	0
0	0	0	0	0	0	0
0	0	0	0	0	0	0
0	0	0	0	0	0	0
0	0	0	0	0	0	0
0	0	0	0	0	0	0
0	0	0	0	0	0	0
0	0	0	0	0	0	0
0	0	0	0	0	0	0
0	0	0	0	0	0	0

0	0	0	0	0	0	0
0	0	0	0	0	0	0
0	0	0	0	0	0	0
0	0	0	0	0	0	0
0	0	0	0	0	0	0
0	0	0	0	0	0	0
0	0,00000864	0	0	0	0	0
0	0,00000864	0	0	0	0	0
0	0,00000864	0	0	0	0	0
0	0,00000864	0	0	0	0	0
0	0,00000864	0	0	0	0	0
0	0,00000864	0	0	0	0	0
0	0,00000864	0	0	0	0	0
0	0	0	0	0	0	0
0	0	0	0	0	0	0
0	0	0	0	0	0	0
0	0	0	0	0	0	0
0	0	0	0	0	0	0
0	0	0	0	0	0	0
0	0	0	0	0	0	0
0	0	0	0	0	0	0
0	0	0	0	0	0	0
0	0	0	0	0	0	0
0	0	0	0	0	0	0
0	0	0	0	0	0	0
0	0	0	0	0	0	0
0	0	0	0	0	0	0
0	0	0	0	0	0	0
0	0	0	0	0	0	0
0	0	0	0	0	0	0
0	0	0	0	0	0	0
0	0	0	0	0	0	0
0	0	0	0	0	0	0
0	0	0	0	0	0	0
0	0	0	0	0	0	0
0	0	0	0	0	0	0
0	0	0	0	0	0	0
0	0	0	0	0	0	0
0	0	0	0	0	0	0
0	0	0	0	0	0	0
0	0	0	0	0	0	0
0	0	0	0	0	0	0
0	0	0	0	0	0	0
0	0	0	0	0	0	0
0	0	0	0	0	0	0
0	0	0	0	0	0	0
0	0	0	0	0	0	0
0	0	0	0	0	0	0
0	0	0	0	0	0	0
0	0	0	0	0	0	0
0	0	0	0	0	0	0
0	0	0	0	0	0	0
0	0	0	0	0	0	0
0	0	0	0	0	0	0
0	0	0	0	0	0	0

0	0	0	0	0	0	7,722E-06
0	0	0	0	0	0	7,722E-06
0	0	0	0	0	0	7,722E-06
0	0	0	0	0	0	7,722E-06
0	0	0	0	0	0	0
0	0	0	0	0	0	0
0	0	0	0	0	0	0
0	0	0	0	0	0	0
0	0	0	0	0	0	0
0	0	0	0	0	0	0
0	0	0	0	0	0	0
0	0	0	0	0	0	0
0	0	0	0	0	0	0
0	0	0	0	0	0	0
0	0	0	0	0	0	0
0	0	0	0	0	0	0
0	0	0	0	0	0	0
0	0	0	0	0	0	0
0	0	0	0	0	0	0
0	0	0	0	0	0	0
0	0	0	0	0	0	0
0	0	0	0	0	0	0
0	0	0	0	0	0	0
0	0	0	0	0	0	0
0	0	0	0	0	0	0
0	0	0	0	0	0	0
0	0	0	0	0	0	0
0	0	0	0	0	0	0
0	0	0	0	0	0	0
0	0	0	0	0	0	0
0	0	0	0	0	0	0
0	0	0	0	0	0	0
0	0	0	0	0	0	0
0	0	0	0	0	0	0
0	0	0	0	0	0	0
0	0	0	0	0	0	0
0	0	0	0	0	0	0
0	0	0	0	0	0	0
0	0	0	0	0	0	0
0	0	0	0	0	0	0
0	0	0	0	0	0	0
0	0	0	0	0	0	0
0	0	0	0	0	0	0
0	0	0	0	0	0	0
0	0	0	0	0	0	0
0	0	0	0	0	0	0
0	0	0	0	0	0	0
0	0	0	0	0	0	0
0	0	0	0	0	0	0
0	0	0	0	0	0	0
0	0	0	0	0	0	0
0	0	0	0	0	0	0
0	0	0	0	0	0	0
0	0	0	0	0	0	0
0,000006171	0	0	0	0	0	0

0	0	0	0	0	0	0
0	0	0	0	0	0	0
0	0	0	0	0	0	0
0	0	0	0	0	0	0
0	0	0	0	0	0	0
0	0	0	0	0	0	0
0	0	0	0	0	0	0
0	0	0	0	0	0	0
0	0	0	0	0	0	0
0	0	0	0	0,00000485	0	0
0	0	0	0	0,00000485	0	0
0	0	0	0	0,00000485	0	0
0	0	0	0	0,00000485	0	0
0	0	0	0	0	0	0
0	0	0	0	0	0	0
0	0	0	0	0	0	0
0	0	0	0	0	0	0
0	0	0	0	0	0	0
0	0	0	0	0	0	0
0	0	0	0	0	0	0
0	0	0	0	0	0	0
0	0	0	0	0	4,262E-06	0
0	0	0	0	0	4,262E-06	0
0	0	0	0	0	4,262E-06	0

Sample28	Samples10	TOTALE	Number of samples in which abundance is abo
0,000155	0,0002212	1,448883775	25
4,32E-05	0,0002336	0,266577771	14
0,061383	0,0002212	0,099970109	17
0,000605	0,0118707	0,400564433	27
0,000985	0,0115973	0,298142661	27
0,00095	3,262E-05	0,012066297	5
0,380192	0,0001103	0,41055356	16
0,00146	0,0001103	0,193905889	11
1,73E-05	9,725E-05	0,413825566	16
0,003792	0,0005135	0,031798474	14
0	3,914E-05	0,321134445	12
0,209551	1,957E-05	0,238135392	5
6,05E-05	0,0005912	0,101123741	21
0,000147	0,0006369	0,067837559	24
0,001088	0	0,173322933	9
0,000276	0,0024562	0,067468926	24
0,002677	1,305E-05	0,042444115	12
0,01483	0	0,131360323	9
0,00044	0,0010787	0,066958992	25
0,003187	0	0,173718941	5
8,64E-06	0	0,161245757	3
0,109465	4,566E-05	0,118916124	7
3,45E-05	0,0004353	0,083241953	17
0	2,609E-05	0,076453892	15
0	0	0,135810248	4
0,000164	0,0007472	0,060825674	22
0	1,957E-05	0,114029029	9
0	0	0,092391249	10
0,000233	0,0007472	0,026988789	13
0	0	0,068926161	8
0	0	0,081120632	1
0,000104	0,0012927	0,015577188	14
1,73E-05	1,957E-05	0,020542742	11
0	6,523E-06	0,024683647	12
3,45E-05	0	0,04854529	12
0	0	0,067264009	2
0	9,725E-05	0,021630305	13
8,64E-06	0,0005005	0,020467278	13
0	0	0,051083746	5
3,45E-05	0,0004548	0,044827664	8
2,59E-05	0,0003119	0,016623964	9
0	9,073E-05	0,018562543	9
0,000164	0,0016308	0,009955341	9
0,009846	0	0,019800823	8
0	0	0,005188864	4
0	0,0001168	0,005779838	10
0	0	0,037892157	2
5,18E-05	6,523E-05	0,013752114	8
0	0	0,036209861	2
0,003748	6,523E-06	0,020567581	8
0	0,0004744	0,016059961	8
0	0,0003706	0,006617392	7
1,73E-05	0,0003766	0,023305011	8
0	0,0001886	0,025801278	9

0,003576	0	0,005188962	5
0	0	0,031835766	5
0	0	0,011520195	7
0,00013	0,0002081	0,019935313	9
4,32E-05	0,0002989	0,017457361	7
0	0	0,024445082	7
0,00013	0,0021117	0,016860855	13
1,73E-05	0,0003315	0,017632167	7
2,59E-05	2,609E-05	0,010833501	4
0,001209	0	0,006827608	8
9,5E-05	0,0011178	0,018658419	10
8,64E-06	0	0,023493224	7
6,91E-05	6,523E-05	0,019774523	11
2,59E-05	3,914E-05	0,014171319	4
0,000199	3,262E-05	0,019363567	8
2,59E-05	0	0,015155904	8
0	9,725E-05	0,012284689	7
0	0	0,022246893	3
0	0	0,024661627	5
5,18E-05	0,0003054	0,007293905	5
0,00013	0,0001103	0,011344449	6
0	0	0,017924509	4
0	0	0,022514603	4
0	5,218E-05	0,014313204	10
0,000682	0,0001103	0,004240808	6
0,001287	0	0,014165098	6
0	1,957E-05	0,02231878	3
0,00418	0	0,005978171	6
0	1,957E-05	0,014653919	7
3,45E-05	0,0001233	0,006576373	5
0	0	0,000027324	2
0	0	0	3
8,64E-06	2,609E-05	0,012058817	7
7,77E-05	0	0,004786746	2
8,64E-05	0,0003961	0,004949316	5
0	0,0001233	0,014958879	4
0	0	0,019527299	6
0,0026	0	0,003173166	5
0	0	0,006782965	2
0	1,305E-05	0,004583185	2
8,64E-06	9,725E-05	0,012838037	6
0,002047	6,523E-06	0,016014513	2
2,59E-05	0	0,000804641	2
0,0128	6,523E-06	0,014982128	3
8,64E-06	5,218E-05	0,000710021	1
0	0	0,004643322	6
0	0	0,012228801	7
0	0	0,01727212	3
0	0	0,016232893	5
0	0	0,011741152	4
0,00032	0,0012862	0,01212979	6
6,91E-05	0,0012672	0,011310468	6
8,64E-06	0	0,003839473	4
0	0	0,000038174	1
1,73E-05	0	0,015787625	5

0	0,0001494	0,000804585	2
0	0	0,007733146	4
8,64E-06	0	0,001665815	8
0	2,609E-05	0,005328235	4
2,59E-05	0,0001299	0,009636077	6
0	0,0001299	0,004379355	6
0	0	0,013475306	3
5,18E-05	3,262E-05	0,00648931	4
0	0	0,004674901	4
0	0	0,012485343	4
0	0	0,000402718	4
0	0	0,004566808	3
8,64E-06	8,421E-05	0,004038736	4
8,64E-06	0,0001233	0,007971137	5
0	0	0,000354596	2
5,18E-05	0	0,012443071	1
0	0	0,000185536	2
4,32E-05	0,0002402	0,006023662	4
0	0	0,000320273	2
1,73E-05	0,0001168	0,007365712	5
0	0	0	1
0	0	0,00485713	3
0	0	0	2
0	0	0,000496587	2
0	1,957E-05	0,005235726	5
0,001114	0	0,012474563	2
0	0	0	2
0	0	0,001443711	2
0	6,523E-06	0,001197585	4
0	0	0,000888786	1
0	0	0	1
1,73E-05	0	0,005590844	3
0	1,957E-05	0,008160925	2
8,64E-06	0,0001951	0,004581058	4
0	0	0,002460551	3
1,73E-05	2,609E-05	0,005738557	3
0	0	0,011748098	3
0	0	0,00226716	2
0	0,0001038	0,005524501	4
0	0	0,001391183	1
0	0	0,006424873	3
0	0	0,01132038	2
0	0	0,009368043	3
0,000104	1,305E-05	0,003329879	1
8,64E-06	1,957E-05	0,010406575	1
2,59E-05	3,262E-05	0,008304429	2
0	0	0,010568404	1
0	0	0,008729242	3
0	0,0001168	0,003053805	4
0	0	0,002044348	1
8,64E-06	0,0003641	0,004923789	3
8,64E-05	0	0,001261793	2
0	0	0,003032091	5
0	0,000169	0,004439364	4
0	0	0,009028553	2

0	0	0,005125805	2
8,64E-06	3,914E-05	0,004888899	2
0,004854	0	0,006163468	2
0	0	0,009396759	1
1,73E-05	6,523E-05	0,004235231	2
6,91E-05	1,305E-05	0,002387424	1
0	0	0,002194857	2
0	0	0,005853957	2
8,64E-06	7,175E-05	0,004113068	3
0	0	0,002633324	2
0	0	0,001275438	3
8,64E-06	3,914E-05	0,005272213	2
0	0	0,006326082	3
8,64E-06	2,609E-05	0,004895357	1
2,59E-05	0,000156	0,00179983	2
0	2,609E-05	0,004667709	1
0	6,523E-06	0,001473306	3
1,73E-05	0	0,005408768	3
0,000328	0	0,00702087	1
0	0	0,00275961	2
0	0	0,000123492	1
0	3,262E-05	0,003138233	1
6,91E-05	0	0,001690953	1
0	0	0,005246557	2
0	6,523E-06	0,005822327	1
0	0	0,007149742	1
0	4,566E-05	0,00248621	1
2,59E-05	1,957E-05	0,003590128	1
8,64E-06	0	0,003178721	2
0	0	0,002242943	3
0	0	0,0005343	2
8,64E-06	0,0001886	0,005357169	1
0	0	0,000717529	2
0	0	0,000126355	3
0	0	0,007085528	1
0	0	0,001690451	3
0	0	0,006865066	1
0	0	0	2
8,64E-06	0	0,002687229	2
0	0	0,001838601	1
8,64E-06	0	0,001525092	1
0	2,609E-05	0,004145098	1
0	8,421E-05	0,001363503	2
0	0,0006885	0,002672853	1
0	3,914E-05	0,006051468	1
8,64E-06	3,914E-05	0,003997337	2
0	0	0,001411533	2
0	0	0,006182389	2
0	0	0,000263661	1
0	0	0,004320582	1
4,32E-05	1,957E-05	0,002237356	1
0,000147	0	0,006193072	2
0	0	0,000025521	1
8,64E-06	0	0,005037762	2
0	0	0,001528387	1

0	1,957E-05	0,001893682	1
0	6,523E-06	0,003840554	0
0	0,0006108	0,001047419	1
0	0	0,000020654	1
0	0	0,005794462	1
0,00038	0	0,003362308	2
0	0,0001103	0,001168708	2
8,64E-06	5,218E-05	0,003800137	1
0	0	0,001802469	1
0	0	0,000039416	4
0	0	0,001358332	1
0	0	0,005508345	1
9,5E-05	0	0,003968772	1
0	0	0,000810208	1
0	0	0,003687467	2
0	0	0,00000485	1
0	0	0,002813164	2
4,32E-05	0	0,000241031	3
0	0	0,0052063	1
0	0	0,000774527	1
1,73E-05	0,0004157	0,00283345	2
1,73E-05	0	0,001637936	1
0	6,523E-06	0,000801303	1
1,73E-05	6,523E-06	0,003248426	0
8,64E-06	5,871E-05	0,002545894	0
0	0	0,000015804	1
0	0	0,001520088	1
0	5,218E-05	0,004824511	3
0	0	0,00001061	1
1,73E-05	1,305E-05	0,00272214	0
0	0	0,00009905	1
0	0	0,00349461	1
0	0	0	1
0	3,262E-05	0,002159487	0
0,000225	6,523E-06	0,001581539	1
0	0	0,003619809	1
1,73E-05	0,0003119	0,002987947	0
0	0	0,00147951	0
5,18E-05	0,0002663	0,003586599	0
0	0	0,000054021	1
0,002721	6,523E-06	0,003222783	1
0	0	0,00137791	2
8,64E-06	0	0,003938479	2
0	0	0,004216885	1
0	0	0,000008606	1
0	0	0,002363905	0
0	2,609E-05	0,003057916	1
0	6,523E-06	0,000036565	1
0	0	0,00143791	0
0	0	0,002542113	2
0	0	0,00000485	1
0	0	0,001332734	1
8,64E-06	0	0,001244601	1
0	6,523E-06	0,00245633	1
0,001771	0	0,001949598	1

8,64E-06	0	0,001584656	0
0	2,609E-05	0,002305424	0
0	0	0,002409846	2
0	0	0,000744466	1
5,18E-05	0	0,001060968	0
0	0	0,003591394	1
0	0	0,003908696	1
0	2,609E-05	0,002256623	0
0	1,305E-05	0,000516308	2
8,64E-06	0	0,001876561	0
0	0	0,001123008	0
0	0	0,001355573	1
0	0	0,003798388	1
0	0	0	1
0	0	0,003520995	1
0	1,305E-05	0,000738908	1
0	0	0,003734782	1
0	0	0,001394157	1
0	0	0,003382183	1
0,000328	0	0,002386841	0
0	0	0,002311135	0
0	2,609E-05	0,00181274	0
0	0	0,000444969	2
0	0	0,00035451	1
8,64E-06	0	0,003335982	1
0	0	0,00332516	1
8,64E-06	0	0,001779757	0
0	0	0,002749234	1
0	1,305E-05	0,001627563	0
0	3,262E-05	0,002527063	1
0	0	0,000007896	1
8,64E-06	0	0,000247703	0
0	0	0,003168197	1
0	0	0,000665111	1
0	0	0,000895398	0
8,64E-06	3,914E-05	0,000873375	0
0	0	0,000917478	1
0,000155	0	0,002682962	1
8,64E-06	4,566E-05	0,00184565	0
1,73E-05	0	0,003008058	1
0	3,262E-05	0,001666207	0
0	0	0,001829566	2
0	0	0	1
0	0	0	1
0	0	0,002843808	1
8,64E-06	0	0,000182105	0
0	0	0,002840776	1
0	0	0,001486868	0
0	0	0,002472954	1
0	0	0,000169904	1
0	0	0,001149772	0
0	0	0,000158668	1
0	0	0,002771734	1
0,000354	0	0,000425455	1
0	0	0,002567812	1

0	0	0,002424726	1
0	0	0,000025772	1
0	0	0,002537448	1
0,000371	0	0,002146079	1
0	0	0,001168031	1
0	3,914E-05	0,001189195	0
2,59E-05	0	0,000151715	0
0	0	0,002612623	0
0	1,305E-05	0,001941277	0
6,05E-05	0	0,000990664	1
0	0,0001168	0,000957275	0
0	0	0,002497066	1
0	0	0,000908426	0
0	1,305E-05	0,000682929	1
0	0	0,001795979	1
1,73E-05	0	0,000647929	0
0	0	0,002025215	0
0	0	0,001091754	1
0	0	0,00012855	1
0	0	0,000139782	1
0	0	0,001997096	1
0	0	0,000035778	0
0	2,609E-05	0,000458711	0
0	0	0	0
0	0	0,000167718	0
1,73E-05	0,0001886	0,001892522	0
0	0	0,000630254	0
0	0	0,001909929	0
0	0	0,002090106	1
0	0	0,00056359	0
8,64E-06	0	0,001166012	0
0	6,523E-06	0,001440094	0
0	1,957E-05	0,000526075	1
0	0	0,001636973	0
2,59E-05	0	0,000561582	0
0	0	0,000161956	0
0	0	0,000340091	0
0	0	0,002270111	1
0	0	0,000287427	1
0	1,957E-05	0,001331281	0
0	0	0,00004685	0
0	0	0,000159172	1
0	0	0,000098008	1
0	0	0,00069249	0
0	0	0,00111941	0
0	0	0,000196442	1
0	0	0,001915691	1
0	0	0,000770166	0
0,001667	0	0,001685861	1
0	0	0,00188566	1
0	0	0,002157774	1
0	0	0,002153658	1
0	1,957E-05	0,001600191	0
0	0	0,001970966	1
0	0	0,000199373	0

2,59E-05	0	0,001982358	1
0	0	0,002119562	1
1,73E-05	0	0,001255817	0
0	0	0,002080025	1
0	0	0,001291378	0
0	0	0,00010658	1
0	0	0,000032076	1
0	0	0,002009036	1
0	6,523E-06	0,000894389	0
0	0	0,002016548	1
0	0	0,001418826	0
0	0	0,000093222	1
0	0	0,000437138	0
0	0	0,001296975	0
0	0	0,001104526	0
0	0	0,001839404	0
8,64E-06	0	0,00123074	0
0	0	0,001898618	1
0	0	0,001440019	0
0	3,914E-05	0,000765146	0
0	1,957E-05	0,00168641	1
0	4,566E-05	0,00080114	0
0	0	0,001360778	0
0	0	0,00160327	0
0	0	0,001808354	0
0	0	0,000028693	0
0	0	0,001769461	1
5,18E-05	0	0,000540726	0
0	0	0	1
9,5E-05	0	0,001626067	1
0	0	0,001103705	0
0	3,262E-05	0,001653199	1
0	0	0,001732092	0
0	0	0,000399624	0
4,32E-05	0,0001103	0,001306519	0
8,64E-06	1,957E-05	0,000725864	0
0	0	0,001685522	1
0	0	0,001062761	0
0,000354	0	0,001598806	0
0	0	0,001463226	1
0	0	0,000390985	0
0	0	0,000872009	0
0	0	0,000239316	0
0	0	0,001655762	1
0	0	0,000912892	0
0	0	0,000921846	0
0	0	0,001484244	0
0	0	0,001460387	1
0	1,305E-05	0,001174154	0
0	0,0002212	0,0012371	0
0,000276	0	0,000352246	1
0	0	0,001381837	1
0	0	0,001449817	0
0	0	0,001536866	1
0	0	0,000535238	0

8,64E-06	0	0,001491901	1
0	0	0	0
0,00032	0	0,000412985	0
0	0	0,001336712	0
0	0	0,000567936	0
0	1,305E-05	0,000462467	0
0,001106	0	0,001128718	1
0	0	0,001461268	1
0	0	0,001123416	0
0	0	0,00000857	1
0	0	0,000158976	1
0	0	0,0014468	1
0	0	0,000701908	0
0	0	0,000231905	0
0	0	0,000676483	0
0	0	0,000621151	0
0	1,305E-05	0,000460141	0
0	6,523E-06	0,000261889	0
0	0	0,001391357	1
0	0	0,000080356	1
0	0	0,000852866	0
0	1,305E-05	0,000757511	0
0	0	0,001358166	0
0	0	0,000297655	0
0	0	0,001345524	1
0	0	0,000519981	0
0	0	0,00134261	1
0	0	0	1
0	0	0,000260721	0
0	0	0,001245149	0
0	6,523E-06	0,000562336	0
0	0	0,001330308	0
0,000726	0	0,001020204	0
0	0	0,000649532	0
0	9,725E-05	0,001193758	0
0	0	0,000021702	0
0	0	0,00126825	1
0	0	0,000463336	0
0	0	0,001217212	0
0	1,305E-05	0,000622442	0
0	0	0,000834824	0
0	0	0,0009128	0
0	0	0,000196955	0
6,05E-05	0	0,000130497	0
0	0	0,000765858	0
0	0	0,00121107	0
0	0	0,000439367	0
0	0	0,001238735	1
0	0	0,000120138	0
0	0	0,000657941	0
0	0	0,00002838	1
0	1,957E-05	0,001045772	0
0	0	0,00122978	1
0	0	0,000119772	1
0	0	0,000885757	0

0	0	0,000580284	0
0	0	0,001209292	1
0	0	0,000407269	0
0	0	0,000028936	0
0	0	0	1
0	0	0,000149141	1
0	0	0,000791353	0
0	0	0,000728089	0
0	0	0,00090204	0
0	0	0,000221642	0
0	0	0,000496328	0
8,64E-06	0	0,001144561	0
8,64E-06	3,262E-05	0,001148424	0
0	6,523E-06	0,00078589	0
0	0	0,00000957	1
0	0	0,000811465	0
0	0	0,00096095	0
0	0	0,000997298	0
0	0	0,000756354	0
0	6,523E-06	0,000452584	0
0	3,914E-05	0,000574251	0
0	0	0,0010575	1
0	0	0,000236861	0
0	0	0	1
0	1,305E-05	0,000261551	0
0	0	0,000094042	0
2,59E-05	3,262E-05	0,000617527	0
0	0	0,000610648	0
0	0	0,001067934	1
0	0	0	0
0	0	0,001052128	0
0	0	0,000535557	0
0	0	0,000656043	0
0	0	0,000735306	0
8,64E-06	0	0,000279392	0
0	0	0,001000604	0
0	0	0,000339733	0
0	0	0,000992178	0
0	0	0,000526213	0
0	0	0,000759437	0
3,45E-05	1,305E-05	0,000699663	0
0	0	0,000619147	0
8,64E-06	0	0,000665015	0
0	0	0,000988432	0
0,000553	0	0,000552768	0
0	0	0,000388138	0
0	0	0,000935412	0
0	0	0,000528204	0
0	0	0,000952135	0
0	0	0,000922447	0
0	6,523E-06	0,000682598	0
0	0	0,000662109	0
1,73E-05	6,523E-06	0,000868911	0
0	0	0,000884466	0
0	0	0,000104792	0

0	0	0,000922128	0
0	0	0	0
0	0	0,000393713	0
0	0	0,000587171	0
8,64E-06	3,262E-05	0,000608954	0
0	6,523E-06	0,000134045	0
0	0	0,000658982	0
0	0	0,000892362	0
0	0	0,000673319	0
0	0	0	0
0	0	0,000884606	0
1,73E-05	9,725E-05	0,00062173	0
0	6,523E-06	0,000866678	0
0	0	0,000854441	0
0	6,523E-06	0,00056202	0
0	0	0,000670374	0
0	0	0,000833148	0
0,00038	0	0,000471155	0
0	0	0,000148989	0
0	0	0,000846708	0
0	4,566E-05	0,000367866	0
0	0	0,000831692	0
0	0	0,000758235	0
0	0	0,000623215	0
0	0	0,000794892	0
0	0	0,000822832	0
0	0	0,000298167	0
0	0	0,000768768	0
0	0	0,000637983	0
0	0	0,000551242	0
0	6,523E-06	0,000627657	0
0	1,305E-05	0,00067618	0
0	0	0,000395369	0
0	0	0,000135282	0
0	0	0,000753742	0
0	0	0,000612084	0
0	0	0	0
0	0	0,000165379	0
0	1,305E-05	0,000139069	0
0	0	0,000736767	0
0	0	0,000221054	0
0	0	0,000710919	0
0	0	0,000082334	0
0	4,566E-05	0,000439147	0
0	0	0	0
0	0	0,000494908	0
0	0	0,000027364	0
0	0	0,00070059	0
0	0	0,000235588	0
0	0	0,000336473	0
0	0	0,000643826	0
0	0	0,000539874	0
0	0	0,000389536	0
0	0	0,000585299	0
0	0	0,000019261	0

0	0	0,000657229	0
0	0	0,000670452	0
0	0	0,000257878	0
0	0	0,000055343	0
0	0	0,0005059	0
0	0	0,000028936	0
0	0	0,000650364	0
0	0	0,000585616	0
0	0	0,000007234	0
0	0	0,000069348	0
0	0	0,000640982	0
0	0	0,000357616	0
0	0	0,00003594	0
0	0	0,000236242	0
0	6,523E-06	0,00024404	0
0	0	0,000506082	0
0	0	0,000612752	0
0	0	0,000185412	0
0	0	0,000283388	0
0	0	0,000119251	0
0	0	0,000404145	0
0	0	0,000193148	0
0	0	0,00000857	0
0	1,957E-05	0,000162347	0
0	0	0,000582912	0
0	0	0,000481616	0
0	0	0,000212773	0
0	0	0	0
0	0	0,000330127	0
0	0	0	0
0	0	0	0
0	0	0,000373297	0
0	0	0	0
0	0	0,000560279	0
0	0	0,000366668	0
0	0	0	0
0	0	0,00054063	0
0	0	0,000467054	0
0	0	0,000542668	0
0	0	0	0
0	6,523E-06	0,000132161	0
0	0	0,000091296	0
0	0	0,000529301	0
0	0	0	0
0	0	0,00050786	0
0	0	0,000507178	0
0	0	0,000093856	0
0	0	0,000014468	0
0	0	0,000020878	0
0,000371	0	0,000394573	0
0,000276	0	0,00039617	0
0,000216	0	0,000241913	0
0	0	0	0
0	0	0,000493862	0
0	0	0,000492861	0

0	0	0,000271051	0
0	0	0,000304612	0
0	0	0,000138119	0
8,64E-06	0	0,000160267	0
0	0	0	0
0	0	0,000074586	0
0	0	0,000044832	0
0	0	0,000421286	0
0	6,523E-06	0,000299339	0
0	0	0	0
0	0	0,000030875	0
0	0	0,000007234	0
0	6,523E-06	0,000115377	0
0	0	0	0
0	0	0,000459275	0
8,64E-06	0	0,000131433	0
0	0	0,000455742	0
0	0	0,000041514	0
0	0	0,000232275	0
0	0	0,000214905	0
0	1,305E-05	0,000343026	0
0	0	0	0
0	0	0,000028936	0
0	0	0,00018688	0
0	0	0,000034342	0
0	0	0,000028632	0
0	0	0,00032263	0
0	1,305E-05	0,00028351	0
0	0	0,000347482	0
0	0	0,000418352	0
0	0	0,000028936	0
0	0	0,000136842	0
0,00013	0	0,000160081	0
0	0	0,000153334	0
0	1,305E-05	0,00015341	0
0	0	0,000025056	0
0	0	0,000425064	0
0	0	0,000167306	0
0	0	0,000421434	0
0	6,523E-06	0,000250309	0
0	0	0,000358414	0
0	6,523E-06	0,000103408	0
0	0	0,000414964	0
0	0	0,000063216	0
0	1,305E-05	0,00021968	0
0	0	0,000225636	0
0	0	0,00013211	0
0	0	0	0
0	0	0,000338436	0
0	0	0,000156269	0
0	0	0,000043916	0
0	0	0,000130368	0
9,5E-05	0	0,000121271	0
0	0	0,000363535	0
0	0	0,000261337	0

0	0	0,00003428	0
0	0	0,000371308	0
0	0	0,0002112	0
0	0	0,000339255	0
0	0	0,000375782	0
0	0	0,000337942	0
0	0	0,00037422	0
0	0	0,000067778	0
0	0	0,000265638	0
0	0	0,000290144	0
0	0	0,000065042	0
8,64E-06	5,871E-05	0,000361664	0
0	0	0,00010718	0
0	0	0,000172183	0
0	0	0	0
0	0	0	0
0	0	0	0
0	0	0,00007234	0
0	0	0,00002871	0
0	0	0,000276466	0
0	0	0,000036321	0
8,64E-06	0	0,000340986	0
0	0	0	0
0	0	0,000145868	0
0	0	0	0
0	0	0,000076348	0
0	0	0,000172895	0
0	6,523E-06	0,000271513	0
0	0	0,000163412	0
0	0	0,000210057	0
0	0	0,000094148	0
8,64E-06	1,305E-05	0,000301141	0
0	0	0,000131206	0
0	0	0,000038558	0
0	0	0,000264248	0
8,64E-06	0	0,000264513	0
0,00013	0	0,000204221	0
0	0	0,000292658	0
0	0	0,00008525	0
0	0	0	0
0	0	0,000316882	0
8,64E-06	1,957E-05	0,000258721	0
0,000285	0	0,000294481	0
0	0	0,000312296	0
0	0	0	0
0	0	0,000104598	0
0	0	0	0
0	0	0,000296954	0
0	0	0,000088897	0
0	0	0	0
0	0	0	0
0	0	0,000230821	0
0	0	0,000007234	0
0	0	0,000254778	0
0	4,566E-05	0,000295565	0

0	0	0,000025772	0
0	0	0,00016848	0
0	0	0,000283399	0
0	0	0,000100348	0
0	0	0	0
0	0	0	0
0	0	0,00015753	0
0	0	0	0
0	0	0,000084591	0
0	0	0,000247338	0
0	0	0,000027873	0
0	0	0,000034091	0
6,05E-05	0	0,000259736	0
0	0	0,0001294	0
0	0	0,000273103	0
0	0	0,000272084	0
0	0	0,000025772	0
0	1,957E-05	0,000227003	0
0	0	0,000028936	0
0,000138	0	0,000198004	0
0	1,305E-05	0,000169523	0
0	0	0,000144621	0
0	0	0,000208953	0
0	0	0,000069634	0
0	2,609E-05	0,000222823	0
0	0	0,00019888	0
0	0	0,000091216	0
0	0	0,000239849	0
0,000104	0	0,000139368	0
0	0	0,00025542	0
0	0	0,000215974	0
0	0	0,000137828	0
0	0	0	0
0	0	0,00008448	0
0	0	0,000130514	0
4,32E-05	0	0,000237764	0
8,64E-06	6,523E-06	0,000151702	0
0	0	0,00011327	0
0	0	0,00024607	0
0	1,305E-05	0,000188455	0
0	0	0,000111252	0
0,000164	0	0,000183023	0
0	0	0,000101462	0
0	0	0,000222728	0
0	0	0,00000857	0
0	0	0,000095378	0
0	0	0	0
0	0	0	0
0	0	0,000238194	0
0	0	0	0
0	0	0	0
0	0	0,000048762	0
0	0	0,00020723	0
0	0	0,000142295	0
8,64E-06	1,957E-05	0,000113096	0

0	0	0,000168951	0
0	0	0,000169545	0
0	0	0,000009663	0
0	0	0	0
0	0	0	0
0	0	0,000229394	0
0	0	0,000007234	0
0	0	0,000064637	0
0	0	0,000188029	0
0	0	0,00001803	0
0	0	0,000119346	0
8,64E-06	0	0,000119653	0
0	0	0	0
0	0	0,000213771	0
9,5E-05	0	0,000147593	0
0	0	0,000050024	0
0	0	0,000054078	0
0	0	0,000055428	0
0	0	0	0
0	0	0,000085563	0
0	0	0,000084834	0
0	1,957E-05	0,000134942	0
0	0	0,000101718	0
0	0	0	0
0	0	0,000187296	0
0,000112	0	0,000143443	0
0	0	0,00009531	0
0	0	0,000080289	0
8,64E-06	0	0,000141149	0
0	0	0,000130476	0
0	0	0,000152728	0
0	0	0,000043536	0
0	0	0,000028936	0
0	0	0,000171879	0
0	0	0,000201114	0
0	3,262E-05	0,000181192	0
0	0	0	0
0	0	0,000007234	0
0	0	0	0
0	0	0,000175584	0
0	1,957E-05	0,000168413	0
0	0	0,000186708	0
0	0	0,000171841	0
0	0	0,00000957	0
0	0	0,000113493	0
0	0	0,00010851	0
0	0	0	0
0	0	0,00005331	0
0	0	0,000155662	0
0	0	0	0
0	6,523E-06	0,000139609	0
0	0	0	0
0	0	0	0
0	0	0,000008448	0
0	0	0,00012827	0

0	0	0	0
0	0	0,00001552	0
0	0	0,000023038	0
0	0	0,000141246	0
0	0	0,000074532	0
0	0	0,000177235	0
0	0	0,00002376	0
0	0	0,00008865	0
0	0	0,000021702	0
0	0	0,000102977	0
0	0	0,000028936	0
0	6,523E-06	0,000140174	0
0	0	0,000151074	0
0	0	0,000041789	0
0,000147	0	0,000151679	0
0	0	0,000170422	0
0	0	0,000069774	0
0	0	0,000087752	0
0	1,305E-05	0,000047326	0
0	0	0,00001152	0
0	0	0,000021702	0
0	0	0,000075084	0
3,45E-05	0	0,000114397	0
0	0	0,000107966	0
0	0	0,000007234	0
0	0	0,00006856	0
0	0	0,000113973	0
0	0	0	0
0	0	0,000054554	0
0	0	0,000112464	0
0	0	0,000159827	0
0	0	0	0
0	0	0,000159148	0
0	0	0	0
0	0	0	0
0	0	0,000155616	0
0	0	0	0
0	1,305E-05	0,000029084	0
0	0	0,00015426	0
3,45E-05	0	0,000098748	0
0	0	0,000043474	0
0	0	0,000094602	0
0	0	0	0
0	0	0,000067778	0
0	0	0	0
0	0	0,000014468	0
0	0	0,00015204	0
0	0	0,000064734	0
0	0	0	0
0	0	0,000057596	0
0	0	0	0
0	6,523E-06	0,000031399	0
0	0	0,000014468	0
0	0	0,000057872	0
0	0	0,000074532	0

0	0	0,000147141	0
0	0	0,000009663	0
0	0	0,0001384	0
0	0	0,000055591	0
0	0	0	0
0	0	0,00000857	0
0	0	0,000024916	0
0	0	0	0
0	0	0,000007234	0
0	0	0,000058798	0
0	0	0,000142504	0
0	0	0,000061147	0
1,73E-05	0	0,000082944	0
0	0	0,000141207	0
0	0	0,000070317	0
0	0	0,000073122	0
0	0	0,000118519	0
0	0	0,000100758	0
0	0	0,000137446	0
0	0	0	0
0	0	0	0
0	0	0,000107424	0
0	0	0,000039794	0
5,18E-05	0	0,000076074	0
0	0	0,000060326	0
0	0	0	0
0	0	0,000104948	0
0	0	0,00009476	0
0	0	0	0
0	0	0,000079623	0
0	0	0,000130376	0
0	0	0,000109256	0
0	0	0,000101276	0
0	0	0,000045612	0
0	0	0,000058654	0
0	0	0,000127116	0
0	0	0,000015804	0
0	0	0,000117705	0
8,64E-06	0	0,000125601	0
2,59E-05	0	0,000025911	0
0	0	0	0
0	0	0	0
6,05E-05	0	0,000074103	0
0	0	0,000113869	0
0	0	0,000104544	0
0	0	0,00011352	0
0	0	0,000121467	0
0	2,609E-05	0,000092851	0
0	0	0,000080642	0
0	0	0,000058124	0
0	0	0,00002881	0
0	0	0,000094686	0
0	0	0,000035036	0
0	0	0,000025056	0
0	0	0,000052268	0

0	0	0	0
0	0	0	0
0	0	0,000116732	0
0	0	0,000077509	0
0	0	0,000012066	0
0	0	0,000115744	0
2,59E-05	0	0,000033145	0
0	0	0,000008688	0
0	0	0,000021702	0
0	0	0,000036658	0
0	0	0	0
0	0	0	0
0	0	0	0
0	0	0	0
0	0	0,000109416	0
0	0	0,000041596	0
0	0	0,000051103	0
0	0	0,00008098	0
0	0	0,000039252	0
0	0	0	0
0	0	0,000023913	0
0	0	0,000081906	0
0	0	0,00000576	0
0	0	0,000105887	0
0	0	0,000105578	0
0	0	0	0
0	0	0,000074177	0
0	0	0,000094708	0
0	0	0,000046064	0
0	0	0	0
0	0	0,000029448	0
0	2,609E-05	0,000062319	0
0	0	0,000101376	0
0	0	0,000101217	0
0	0	0	0
0	0	0	0
0	0	0,000070088	0
6,05E-05	0	0,000069919	0
0	0	0,000043514	0
0	0	0,00001892	0
0	0	0,000038506	0
0	0	0,000089236	0
0	0	0,000015804	0
0	0	0,000097938	0
0	0	0,000050084	0
0	0	0,000066643	0
0	0	0,000095649	0
0	0	0,000095282	0
0	0	0,000066669	0
0	0	0,00001425	0
0	0	0,000085673	0
8,64E-05	0	0,000094818	0
0	0	0,000030784	0
0	0	0,000025588	0
0	0	0,000094042	0

0	0	0,000051756	0
0	0	0,000049021	0
0	0	0,000014468	0
0	0	0	0
0	0	0,000082731	0
0	0	0,000048818	0
0	0	0,00004774	0
0	0	0,000080962	0
0	0	0,000038978	0
0	0	0,00000864	0
0	0	0,00008998	0
0	0	0,000089425	0
0	0	0	0
0	0	0,00006667	0
0	0	0	0
0	0	0	0
0	0	0,000028936	0
0	0	0,000086808	0
0	0	0,00004228	0
0	0	0,0000857	0
0	0	0,000056136	0
0	0	0,000028936	0
0	0	0,000083738	0
0	0	0,000083253	0
0	0	0,000082438	0
0	0	0	0
0	0	0	0
0	0	0,00005999	0
0	0	0,000081906	0
0	0	0	0
0	0	0,000028936	0
0	0	0,000028936	0
0	0	0	0
0	1,305E-05	0,000068091	0
0	0	0,000067126	0
0	0	0,000080297	0
0	0	0,000004262	0
0	0	0	0
0	0	0,000011496	0
0	0	0,000015804	0
0	0	0,000047552	0
1,73E-05	0	0,00004883	0
0	0	0,000058414	0
0	0	0	0
0	0	0,000025728	0
0	0	0,000007234	0
0	0	0,000076224	0
0	0	0,000076	0
0	0	0,000051372	0
0	0	0	0
0	5,871E-05	0,000074581	0
0	0	0	0
0	0	0,000065106	0
0	0	0,000063916	0
0	0	0,000060888	0

0	0	0	0
0	0	0,000023307	0
0	0	0	0
0	0	0,000007234	0
0	0	0,000033388	0
0	0	0	0
0	0	0,000052671	0
0	0	0,000022214	0
0	0	0	0
0	0	0	0
0	0	0,000019123	0
0	0	0	0
0	0	0,000026154	0
0	0	0,000069512	0
0	2,609E-05	0,000069016	0
0	0	0	0
0	0	0,000067535	0
0	0	0	0
0	0	0,00006647	0
0	0	0,000066343	0
0	0	0,000057162	0
0	0	0,000049005	0
0	0	0,000052269	0
0	0	0	0
0	0	0,000065188	0
0	0	0	0
0	0	0,00004285	0
0	0	0,000053794	0
0	0	0,000018754	0
0	0	0,0000572	0
0	0	0,000063558	0
0	0	0,000020878	0
0	0	0,000063164	0
0	0	0,000021654	0
0	0	0,00006225	0
0	0	0,000009663	0
0	0	0,000007234	0
0	0	0,000029526	0
0	0	0,000060634	0
0	0	0,00005999	0
0	0	0	0
0	0	0,00001342	0
0	0	0,000041514	0
0	0	0	0
0	0	0,000021702	0
0	0	0,000059375	0
0	0	0	0
0	0	0,000058654	0
0	0	0,000016416	0
0	0	0,000030784	0
0	0	0	0
0	0	0,000029448	0
0	0	0,000057872	0
0	0	0,000057648	0
0	0	0,000038842	0

0	0	0,00000857	0
0	0	0,000057403	0
0	0	0,000026437	0
0	0	0,00002671	0
0	0	0,000027571	0
0	0	0,00005676	0
0	6,523E-06	0,000050141	0
0	0	0	0
0	0	0,00005642	0
0	0	0,000017018	0
0	0	0	0
0	0	0,000029211	0
0	0	0	0
0	0	0	0
0	0	0	0
0	0	0	0
2,59E-05	0	0,000054714	0
0	0	0	0
0	0	0	0
0	0	0,000044885	0
0	0	0,000053678	0
0	0	0,000004262	0
0	0	0,000053022	0
0	0	0	0
0	0	0,00005277	0
0	0	0	0
0	0	0	0
0	0	0	0
0	0	0,00005238	0
0	0	0,00005215	0
0	0	0,000042806	0
0	0	0,000051756	0
0	0	0	0
0	0	0,00005142	0
0	0	0,000051418	0
2,59E-05	0	0,000051378	0
0	0	0,00004224	0
0	0	0,00001873	0
0	0	0,00001425	0
0	0	0,000050638	0
0	0	0,000050638	0
0	0	0,000040335	0
0	0	0,00002771	0
0	0	0	0
0	0	0	0
0	0	0,000050158	0
0	0	0,000050158	0
0	0	0	0
0	0	0	0
0	0	0,000013644	0
0	0	0	0
0	0	0,000007234	0
0	0	0,000007234	0
0	0	0,000049005	0
0	0	0,000048547	0

0	0	0	0
0	0	0,000030855	0
0	0	0,000008448	0
0	0	0,00004752	0
0	0	0,00000857	0
0	0	0,00000946	0
0	0	0,000046951	0
0	0	0	0
0	0	0	0
0	0	0	0
0	0	0,00004608	0
0	0	0,00004608	0
0	0	0,000045912	0
0	0	0	0
0	0	0,000022214	0
0	0	0,000045125	0
0	0	0,000024684	0
0	6,523E-06	0,000030897	0
0	0	0,000044311	0
3,45E-05	0	0,000044211	0
8,64E-06	0	0,00002547	0
0	0	0,00001425	0
0	0	0,000009663	0
0	0	0,000008448	0
0	0	0,000004262	0
0	0	0,000042924	0
0	0	0,000042924	0
0	0	0,000042924	0
0	0	0,000042924	0
0	0	0,000042924	0
0	0	0,00000857	0
0	0	0,000042834	0
0	0	0,000042834	0
0	0	0,000042834	0
0	0	0	0
0	0	0,00004216	0
0	0	0	0
0	0	0	0
0	0	0	0
0	0	0,000013644	0
0	0	0,00004193	0
0	0	0,00004171	0
0	0	0,000024684	0
0	0	0,000018594	0
0	0	0	0
0	0	0	0
0	0	0,000040842	0
0	0	0,000007234	0
0	0	0,000030855	0
0	0	0	0
2,59E-05	0	0,000039555	0
0	0	0,000039416	0
0	0	0	0
0	0	0,00000857	0
0	0	0,000038842	0

0	0	0,00001714	0
0	0	0,000038559	0
0	0	0,000019233	0
0	0	0,000019944	0
0	0	0,000022594	0
0	0	0,00000857	0
0	0	0,000038043	0
0	0	0,00001425	0
0	0	0,000037426	0
0	0	0	0
0	0	0,000028803	0
0	0	0	0
0	0	0	0
0	0	0	0
0	0	0,00000864	0
0	0	0	0
1,73E-05	0	0,000036507	0
2,59E-05	0	0,000025911	0
0	0	0,00003617	0
0	0	0,00003617	0
0	0	0,00001152	0
0	0	0,000027462	0
0	0	0,000035944	0
0	0	0,000007234	0
0	0	0,000007234	0
0	0	0,000007722	0
0	6,523E-06	0,000006523	0
0	6,523E-06	0,000006523	0
0	0	0,00000576	0
0	0	0,000015682	0
0	0	0,00001224	0
0	0	0,000021702	0
2,59E-05	0	0,000034551	0
0	0	0,000015804	0
0	0	0	0
0	0	0	0
0	0	0	0
0	0	0	0
0	0	0,00003428	0
0	0	0,000024684	0
0	0	0,000033834	0
0	0	0,00000485	0
0	0	0,000033388	0
8,64E-06	0	0,000022887	0
0	0	0,00002377	0
0	0	0,000013644	0
0	6,523E-06	0,000032985	0
0	0	0,000032705	0
0	0	0,000014468	0
0	0	0,000023108	0
0	0	0,000023104	0
0	0	0,000023038	0
0	0	0,000031268	0
0	0	0,000021702	0
0	0	0,000030855	0

0	0	0,000021702	0
0	0	0,00000857	0
0	6,523E-06	0,000006523	0
0	0	0,000020639	0
0	0	0,000029622	0
0	0	0	0
0	0	0	0
0	0	0	0
0	0	0	0
0	0	0	0
0	0	0	0
0	0	0	0
0	0	0	0
0	0	0,00001552	0
0	0	0,000028989	0
0	0	0,000028989	0
0	0	0,000028936	0
0	0	0,000028936	0
0	0	0,000028936	0
0	0	0,000028936	0
0	0	0,000028936	0
0	0	0,000028936	0
0	0	0,000028936	0
0	0	0,000028936	0
0	0	0,000028936	0
0	0	0,0000288	0
0	0	0,00002871	0
0	0	0,000028693	0
0	0	0,0000286	0
0	0	0,0000286	0
0	0	0	0
0	0	0	0
0	0	0	0
0	0	0	0
0	0	0	0
0	0	0	0
0	0	0	0
0	0	0	0
0	0	0	0
0	0	0	0
0	0	0	0
0	0	0	0
0	0	0	0
0	0	0	0
0	0	0	0
0	0	0	0
0	0	0	0
0	0	0	0
0	0	0	0
0	0	0	0
0	0	0,000028583	0
0	0	0	0
0	0	0	0
0	0	0,00002849	0
0	0	0,000009663	0

0	0	0,000009663	0
0	0	0	0
0	0	0	0
0	0	0	0
8,64E-06	0	0,000008637	0
0	0	0,000017908	0
0	0	0,000014917	0
0	0	0	0
0	0	0	0
0	0	0	0
0	0	0	0
0	0	0,00001892	0
0	0	0,000014468	0
0	0	0,000017324	0
0	0	0	0
0	0	0,000016416	0
0	0	0,000007234	0
0	0	0,000026357	0
0	2,609E-05	0,000026092	0
0	2,609E-05	0,000026092	0
0	0	0,000025772	0
0	0	0,000025772	0
0	0	0	0
0	0	0,00002571	0
0	0	0,000007234	0
0	0	0,000007234	0
0	6,523E-06	0,000025646	0
0	0	0	0
0	0	0	0
0	0	0,000025521	0
0	0	0,000007234	0
0	0	0,000015423	0
0	0	0,000025374	0
0	0	0,000025374	0
0	0	0,000025374	0
0	0	0,000025374	0
0	0	0,00001552	0
0	0	0,000014468	0
0	0	0,000024526	0
8,64E-06	0	0,000024441	0
0	0	0,000024374	0
0	0	0,000024131	0
0	0	0	0
0	0	0	0
0	0	0	0
0	0	0	0
0	0	0	0
0	0	0	0
0	0	0	0
0	0	0	0
0	0	0	0
0	0	0,000023862	0
0	0	0,00002382	0
0	0	0,00002376	0
0	0	0,00002376	0

0	0	0,00001067	0
0	0	0,000014468	0
0	0	0,00001425	0
0	0	0,00001425	0
0	0	0,000023104	0
0	0	0,00002282	0
0	0	0,00002282	0
0	0	0,00002282	0
0	0	0	0
0	0	0,000013644	0
0	0	0,000013644	0
0	0	0,000022284	0
0	0	0	0
0	0	0,000008448	0
0	0	0,000022146	0
0	0	0,000022092	0
0	0	0	0
0	0	0,00001152	0
0	0	0,000021702	0
0	0	0,000021702	0
0	0	0,000021702	0
0	0	0,000021702	0
0	0	0,000021702	0
0	0	0,000021702	0
0	0	0,000021177	0
0	0	0	0
0	0	0	0
0	0	0	0
0	0	0	0
0	0	0	0
0	0	0,000004262	0
0	0	0,000007896	0
0	0	0	0
0	0	0,000010655	0
0	0	0	0
0	0	0,00000957	0
0	0	0	0
0	0	0,00000946	0
0	0	0,00000946	0
0	0	0,000009663	0
0	0	0	0
0	1,957E-05	0,000019569	0
0	0	0	0
0	0	0,000019326	0
0	0	0,000019326	0
0	0	0	0
0	0	0	0
0	0	0	0
0	0	0	0
0	0	0	0
0	0	0	0
0	0	0,00001914	0
0	0	0,000019123	0
0	0	0,000019123	0
0	0	0	0

0	0	0,00001903	0
0	0	0,00001892	0
0	0	0,00000957	0
0	6,523E-06	0,000018865	0
0	0	0	0
0	0	0,00000946	0
0	0	0,000009663	0
0	0	0,00000957	0
0	0	0	0
0	0	0	0
0	0	0	0
0	0	0	0
0	0	0	0
0	0	0	0
0	0	0	0
0	0	0	0
0	0	0	0
0	0	0,000018513	0
0	0	0,00000857	0
0	0	0	0
0	0	0	0
0	0	0,000008448	0
0	0	0,000018325	0
0	0	0,000018233	0
8,64E-06	0	0,000018207	0
0	0	0	0
0	0	0	0
0	0	0	0
0	0	0	0
0	0	0	0
0	0	0	0
0	0	0,000007896	0
0	0	0,000018111	0
0	0	0,000018102	0
0	0	0,00001803	0
0	0	0,000018018	0
0	0	0,000017908	0
0	0	0,00000857	0
0	0	0,00000857	0
0	0	0,00000857	0
0	0	0,00000857	0
0	0	0	0
0	0	0,000008448	0
0	0	0,000007234	0
0	0	0,000017324	0
0	0	0,00001728	0
1,73E-05	0	0,000017274	0
1,73E-05	0	0,000017274	0
8,64E-06	0	0,000017207	0
0	0	0,00001714	0
8,64E-06	0	0,000017085	0
0	0	0,000017018	0
0	0	0	0
0	0	0	0
0	0	0	0

0	0	0	0
0	0	0,000016896	0
0	0	0,000016896	0
0	0	0,000016804	0
0	0	0,000016804	0
0	0	0,000016804	0
0	0	0	0
0	0	0,000016694	0
0	0	0,000007234	0
0	0	0,000007234	0
0	0	0,000016416	0
0	0	0,000016416	0
0	0	0,000007234	0
0	0	0,000007234	0
0	0	0,000007234	0
0	0	0,000007234	0
0	0	0,000016038	0
0	0	0,000015896	0
0	0	0,000015804	0
0	0	0,000015804	0
0	0	0,000015804	0
0	0	0,000015804	0
0	0	0,000015804	0
0	0	0,000015804	0
0	0	0,000015792	0
0	0	0,000015792	0
0	0	0,000015682	0
0	6,523E-06	0,000006523	0
0	0	0	0
0	0	0	0
0	0	0,00000857	0
0	0	0,000014917	0
0	0	0,00000576	0
0	0	0,000014468	0
0	0	0,000014468	0
0	0	0,000014468	0
0	0	0,000014468	0
0	0	0,000014468	0
0	0	0,000014468	0
0	0	0,000014468	0
0	0	0,000014468	0
0	0	0,000014468	0
0	0	0,000014468	0
0	6,523E-06	0,000014419	0
0	0	0,00001425	0
0	0	0,00001425	0
0	0	0,00001425	0
0	0	0,00001425	0
0	0	0,00001425	0
0	0	0,00001425	0
0	0	0,00001425	0
0	6,523E-06	0,000013757	0
0	0	0	0
0	0	0	0
0	0	0	0

0	0	0	0
0	0	0	0
0	0	0	0
0	0	0	0
0	0	0	0
0	0	0	0
0	0	0,000013644	0
0	0	0,000013644	0
0	0	0,000013644	0
0	0	0,000013644	0
0	0	0,000013644	0
0	0	0,000013644	0
0	0	0,000013644	0
0	0	0,000013644	0
0	0	0,000013644	0
0	0	0,000013644	0
0	0	0,000004262	0
0	1,305E-05	0,000013046	0
0	6,523E-06	0,000006523	0
0	0	0	0
0	0	0	0
0	0	0	0
0	0	0	0
0	0	0	0
0	0	0	0
0	0	0	0
0	0	0,000012914	0
0	0	0,000012902	0
0	0	0	0
0	0	0,000012342	0
0	0	0,000012342	0
0	0	0,00001224	0
0	0	0,000011931	0
0	0	0,000010655	0
0	0	0,000010655	0
0	0	0	0
0	0	0	0
0	0	0	0
0	0	0	0
0	0	0	0
0	0	0	0
0	0	0	0
0	0	0	0
0	0	0	0
0	0	0	0
0	0	0	0
0	0	0	0
0	0	0	0
0	0	0	0
0	0	0,000009663	0
0	0	0,000009663	0
0	0	0,000009663	0
0	0	0,000009663	0
0	0	0,000009663	0
0	0	0,000009663	0

0	0	0,000009663	0
0	0	0,000009663	0
0	0	0,000009663	0
0	0	0,000009663	0
0	0	0,00000957	0
0	0	0,00000957	0
0	0	0,00000957	0
0	0	0,00000957	0
0	0	0,00000957	0
0	0	0,00000957	0
0	0	0,00000957	0
0	0	0,00000946	0
0	0	0,00000946	0
0	0	0,00000946	0
0	0	0,00000946	0
0	0	0,00000946	0
0	0	0,00000946	0
0	0	0,00000946	0
0	0	0,00000946	0
0	0	0,00000946	0
0	0	0,00000946	0
0	0	0,00000946	0
0	0	0	0
0	0	0	0
0	0	0	0
0	0	0	0
0	0	0	0
0	0	0	0
0	0	0	0
0	0	0	0
0	0	0	0
0	0	0	0
0	0	0	0
0	0	0	0
0	0	0,000009194	0
0	0	0	0
0	0	0	0
0	0	0	0
0	0	0	0
0	0	0	0
0	0	0	0
0	0	0	0
0	0	0	0
0	0	0	0
0	0	0	0
0	0	0	0
0	0	0	0
0	0	0	0
0	0	0	0
0	0	0	0
0	0	0	0
0	0	0	0
0	0	0	0
0	0	0	0
0	0	0	0

0	0	0	0
0	0	0	0
0	0	0	0
0	0	0,000008688	0
0	0	0,000008662	0
0	0	0,000008662	0
0	0	0,00000864	0
0	0	0,00000864	0
0	0	0,00000864	0
0	0	0,00000864	0
0	0	0,00000864	0
0	0	0,00000864	0
0	0	0,00000864	0
8,64E-06	0	0,000008637	0
0	0	0,00000857	0
0	0	0,00000857	0
0	0	0,00000857	0
0	0	0,00000857	0
0	0	0,00000857	0
0	0	0,00000857	0
0	0	0,00000857	0
0	0	0,00000857	0
0	0	0,00000857	0
0	0	0,00000857	0
0	0	0,00000857	0
0	0	0,00000857	0
0	0	0,00000857	0
0	0	0,00000857	0
0	0	0,00000857	0
0	0	0,00000857	0
0	0	0,00000857	0
0	0	0,00000857	0
0	0	0,00000857	0
0	0	0,00000857	0
0	0	0,00000857	0
0	0	0,00000857	0
0	0	0,00000857	0
0	0	0,00000857	0
0	0	0,00000857	0
0	0	0,00000857	0
0	0	0,00000857	0
0	0	0	0
0	0	0	0
0	0	0	0
0	0	0	0
0	0	0	0
0	0	0	0
0	0	0,000008448	0
0	0	0,000008448	0
0	0	0,000008448	0
0	0	0,000008448	0
0	0	0,000008448	0
0	0	0,000007896	0
0	0	0,000007896	0

0	0	0,000007722	0
0	0	0,000007722	0
0	0	0,000007722	0
0	0	0,000007722	0
0	0	0,000007234	0
0	0	0,000007234	0
0	0	0,000007234	0
0	0	0,000007234	0
0	0	0,000007234	0
0	0	0,000007234	0
0	0	0,000007234	0
0	0	0,000007234	0
0	0	0,000007234	0
0	0	0,000007234	0
0	0	0,000007234	0
0	0	0,000007234	0
0	0	0,000007234	0
0	0	0,000007234	0
0	0	0,000007234	0
0	0	0,000007234	0
0	0	0,000007234	0
0	0	0,000007234	0
0	0	0,000007234	0
0	0	0,000007234	0
0	0	0,000007234	0
0	0	0,000007234	0
0	0	0,000007234	0
0	0	0,000007234	0
0	0	0,000007234	0
0	0	0,000007234	0
0	0	0,000007234	0
0	0	0,000007234	0
0	0	0,000007234	0
0	0	0,000007234	0
0	0	0,000007234	0
0	0	0,000007234	0
0	0	0,000007234	0
0	0	0,000007234	0
0	0	0,000007234	0
0	0	0,000007234	0
0	0	0,000007234	0
0	0	0,000007234	0
0	0	0,000007234	0
0	0	0,000007234	0
0	0	0,000007234	0
0	0	0,000007234	0
0	6,523E-06	0,000006523	0
0	6,523E-06	0,000006523	0
0	6,523E-06	0,000006523	0
0	0	0	0
0	0	0	0
0	0	0	0
0	0	0	0
0	0	0	0
0	0	0,000006171	0

0	0	0,00000576	0
0	0	0,00000576	0
0	0	0,00000576	0
0	0	0,00000576	0
0	0	0,00000576	0
0	0	0,00000576	0
0	0	0,00000576	0
0	0	0,00000576	0
0	0	0,00000576	0
0	0	0,00000485	0
0	0	0,00000485	0
0	0	0,00000485	0
0	0	0,00000485	0
0	0	0,000004344	0
0	0	0,000004344	0
0	0	0,000004344	0
0	0	0,000004344	0
0	0	0,000004344	0
0	0	0,000004344	0
0	0	0,000004344	0
0	0	0,000004344	0
0	0	0,000004262	0
0	0	0,000004262	0
0	0	0,000004262	0

ove 0.1%
